# The repertoire of mutational signatures in human cancer

**DOI:** 10.1038/s41586-020-1943-3

**Published:** 2020-02-05

**Authors:** Ludmil B. Alexandrov, Jaegil Kim, Nicholas J. Haradhvala, Mi Ni Huang, Alvin Wei Tian Ng, Yang Wu, Arnoud Boot, Kyle R. Covington, Dmitry A. Gordenin, Erik N. Bergstrom, S. M. Ashiqul Islam, Nuria Lopez-Bigas, Leszek J. Klimczak, John R. McPherson, Sandro Morganella, Radhakrishnan Sabarinathan, David A. Wheeler, Ville Mustonen, PCAWG Mutational Signatures Working Group, Ludmil B. Alexandrov, Erik N. Bergstrom, Arnoud Boot, Paul Boutros, Kin Chan, Kyle R. Covington, Akihiro Fujimoto, Gad Getz, Dmitry A. Gordenin, Nicholas J. Haradhvala, Mi Ni Huang, S. M. Ashiqul Islam, Marat Kazanov, Jaegil Kim, Leszek J. Klimczak, Nuria Lopez-Bigas, Michael Lawrence, Iñigo Martincorena, John R. McPherson, Sandro Morganella, Ville Mustonen, Hidewaki Nakagawa, Alvin Wei Tian Ng, Paz Polak, Stephenie Prokopec, Steven A. Roberts, Steven G. Rozen, Radhakrishnan Sabarinathan, Natalie Saini, Tatsuhiro Shibata, Yuichi Shiraishi, Michael R. Stratton, Bin Tean Teh, Ignacio Vázquez-García, David A. Wheeler, Yang Wu, Fouad Yousif, Willie Yu, Gad Getz, Steven G. Rozen, Michael R. Stratton, PCAWG Consortium, Lauri A. Aaltonen, Federico Abascal, Adam Abeshouse, Hiroyuki Aburatani, David J. Adams, Nishant Agrawal, Keun Soo Ahn, Sung-Min Ahn, Hiroshi Aikata, Rehan Akbani, Kadir C. Akdemir, Hikmat Al-Ahmadie, Sultan T. Al-Sedairy, Fatima Al-Shahrour, Malik Alawi, Monique Albert, Kenneth Aldape, Ludmil B. Alexandrov, Adrian Ally, Kathryn Alsop, Eva G. Alvarez, Fernanda Amary, Samirkumar B. Amin, Brice Aminou, Ole Ammerpohl, Matthew J. Anderson, Yeng Ang, Davide Antonello, Pavana Anur, Samuel Aparicio, Elizabeth L. Appelbaum, Yasuhito Arai, Axel Aretz, Koji Arihiro, Shun-ichi Ariizumi, Joshua Armenia, Laurent Arnould, Sylvia Asa, Yassen Assenov, Gurnit Atwal, Sietse Aukema, J. Todd Auman, Miriam R. R. Aure, Philip Awadalla, Marta Aymerich, Gary D. Bader, Adrian Baez-Ortega, Matthew H. Bailey, Peter J. Bailey, Miruna Balasundaram, Saianand Balu, Pratiti Bandopadhayay, Rosamonde E. Banks, Stefano Barbi, Andrew P. Barbour, Jonathan Barenboim, Jill Barnholtz-Sloan, Hugh Barr, Elisabet Barrera, John Bartlett, Javier Bartolome, Claudio Bassi, Oliver F. Bathe, Daniel Baumhoer, Prashant Bavi, Stephen B. Baylin, Wojciech Bazant, Duncan Beardsmore, Timothy A. Beck, Sam Behjati, Andreas Behren, Beifang Niu, Cindy Bell, Sergi Beltran, Christopher Benz, Andrew Berchuck, Anke K. Bergmann, Erik N. Bergstrom, Benjamin P. Berman, Daniel M. Berney, Stephan H. Bernhart, Rameen Beroukhim, Mario Berrios, Samantha Bersani, Johanna Bertl, Miguel Betancourt, Vinayak Bhandari, Shriram G. Bhosle, Andrew V. Biankin, Matthias Bieg, Darell Bigner, Hans Binder, Ewan Birney, Michael Birrer, Nidhan K. Biswas, Bodil Bjerkehagen, Tom Bodenheimer, Lori Boice, Giada Bonizzato, Johann S. De Bono, Arnoud Boot, Moiz S. Bootwalla, Ake Borg, Arndt Borkhardt, Keith A. Boroevich, Ivan Borozan, Christoph Borst, Marcus Bosenberg, Mattia Bosio, Jacqueline Boultwood, Guillaume Bourque, Paul C. Boutros, G. Steven Bova, David T. Bowen, Reanne Bowlby, David D. L. Bowtell, Sandrine Boyault, Rich Boyce, Jeffrey Boyd, Alvis Brazma, Paul Brennan, Daniel S. Brewer, Arie B. Brinkman, Robert G. Bristow, Russell R. Broaddus, Jane E. Brock, Malcolm Brock, Annegien Broeks, Angela N. Brooks, Denise Brooks, Benedikt Brors, Søren Brunak, Timothy J. C. Bruxner, Alicia L. Bruzos, Alex Buchanan, Ivo Buchhalter, Christiane Buchholz, Susan Bullman, Hazel Burke, Birgit Burkhardt, Kathleen H. Burns, John Busanovich, Carlos D. Bustamante, Adam P. Butler, Atul J. Butte, Niall J. Byrne, Anne-Lise Børresen-Dale, Samantha J. Caesar-Johnson, Andy Cafferkey, Declan Cahill, Claudia Calabrese, Carlos Caldas, Fabien Calvo, Niedzica Camacho, Peter J. Campbell, Elias Campo, Cinzia Cantù, Shaolong Cao, Thomas E. Carey, Joana Carlevaro-Fita, Rebecca Carlsen, Ivana Cataldo, Mario Cazzola, Jonathan Cebon, Robert Cerfolio, Dianne E. Chadwick, Dimple Chakravarty, Don Chalmers, Calvin Wing Yiu Chan, Kin Chan, Michelle Chan-Seng-Yue, Vishal S. Chandan, David K. Chang, Stephen J. Chanock, Lorraine A. Chantrill, Aurélien Chateigner, Nilanjan Chatterjee, Kazuaki Chayama, Hsiao-Wei Chen, Jieming Chen, Ken Chen, Yiwen Chen, Zhaohong Chen, Andrew D. Cherniack, Jeremy Chien, Yoke-Eng Chiew, Suet-Feung Chin, Juok Cho, Sunghoon Cho, Jung Kyoon Choi, Wan Choi, Christine Chomienne, Zechen Chong, Su Pin Choo, Angela Chou, Angelika N. Christ, Elizabeth L. Christie, Eric Chuah, Carrie Cibulskis, Kristian Cibulskis, Sara Cingarlini, Peter Clapham, Alexander Claviez, Sean Cleary, Nicole Cloonan, Marek Cmero, Colin C. Collins, Ashton A. Connor, Susanna L. Cooke, Colin S. Cooper, Leslie Cope, Vincenzo Corbo, Matthew G. Cordes, Stephen M. Cordner, Isidro Cortés-Ciriano, Kyle Covington, Prue A. Cowin, Brian Craft, David Craft, Chad J. Creighton, Yupeng Cun, Erin Curley, Ioana Cutcutache, Karolina Czajka, Bogdan Czerniak, Rebecca A. Dagg, Ludmila Danilova, Maria Vittoria Davi, Natalie R. Davidson, Helen Davies, Ian J. Davis, Brandi N. Davis-Dusenbery, Kevin J. Dawson, Francisco M. De La Vega, Ricardo De Paoli-Iseppi, Timothy Defreitas, Angelo P. Dei Tos, Olivier Delaneau, John A. Demchok, Jonas Demeulemeester, German M. Demidov, Deniz Demircioğlu, Nening M. Dennis, Robert E. Denroche, Stefan C. Dentro, Nikita Desai, Vikram Deshpande, Amit G. Deshwar, Christine Desmedt, Jordi Deu-Pons, Noreen Dhalla, Neesha C. Dhani, Priyanka Dhingra, Rajiv Dhir, Anthony DiBiase, Klev Diamanti, Li Ding, Shuai Ding, Huy Q. Dinh, Luc Dirix, HarshaVardhan Doddapaneni, Nilgun Donmez, Michelle T. Dow, Ronny Drapkin, Oliver Drechsel, Ruben M. Drews, Serge Serge, Tim Dudderidge, Ana Dueso-Barroso, Andrew J. Dunford, Michael Dunn, Lewis Jonathan Dursi, Fraser R. Duthie, Ken Dutton-Regester, Jenna Eagles, Douglas F. Easton, Stuart Edmonds, Paul A. Edwards, Sandra E. Edwards, Rosalind A. Eeles, Anna Ehinger, Juergen Eils, Roland Eils, Adel El-Naggar, Matthew Eldridge, Kyle Ellrott, Serap Erkek, Georgia Escaramis, Shadrielle M. G. Espiritu, Xavier Estivill, Dariush Etemadmoghadam, Jorunn E. Eyfjord, Bishoy M. Faltas, Daiming Fan, Yu Fan, William C. Faquin, Claudiu Farcas, Matteo Fassan, Aquila Fatima, Francesco Favero, Nodirjon Fayzullaev, Ina Felau, Sian Fereday, Martin L. Ferguson, Vincent Ferretti, Lars Feuerbach, Matthew A. Field, J. Lynn Fink, Gaetano Finocchiaro, Cyril Fisher, Matthew W. Fittall, Anna Fitzgerald, Rebecca C. Fitzgerald, Adrienne M. Flanagan, Neil E. Fleshner, Paul Flicek, John A. Foekens, Kwun M. Fong, Nuno A. Fonseca, Christopher S. Foster, Natalie S. Fox, Michael Fraser, Scott Frazer, Milana Frenkel-Morgenstern, William Friedman, Joan Frigola, Catrina C. Fronick, Akihiro Fujimoto, Masashi Fujita, Masashi Fukayama, Lucinda A. Fulton, Robert S. Fulton, Mayuko Furuta, P. Andrew Futreal, Anja Füllgrabe, Stacey B. Gabriel, Steven Gallinger, Carlo Gambacorti-Passerini, Jianjiong Gao, Shengjie Gao, Levi Garraway, Øystein Garred, Erik Garrison, Dale W. Garsed, Nils Gehlenborg, Josep L. L. Gelpi, Joshy George, Daniela S. Gerhard, Clarissa Gerhauser, Jeffrey E. Gershenwald, Mark Gerstein, Moritz Gerstung, Gad Getz, Mohammed Ghori, Ronald Ghossein, Nasra H. Giama, Richard A. Gibbs, Bob Gibson, Anthony J. Gill, Pelvender Gill, Dilip D. Giri, Dominik Glodzik, Vincent J. Gnanapragasam, Maria Elisabeth Goebler, Mary J. Goldman, Carmen Gomez, Santiago Gonzalez, Abel Gonzalez-Perez, Dmitry A. Gordenin, James Gossage, Kunihito Gotoh, Ramaswamy Govindan, Dorthe Grabau, Janet S. Graham, Robert C. Grant, Anthony R. Green, Eric Green, Liliana Greger, Nicola Grehan, Sonia Grimaldi, Sean M. Grimmond, Robert L. Grossman, Adam Grundhoff, Gunes Gundem, Qianyun Guo, Manaswi Gupta, Shailja Gupta, Ivo G. Gut, Marta Gut, Jonathan Göke, Gavin Ha, Andrea Haake, David Haan, Siegfried Haas, Kerstin Haase, James E. Haber, Nina Habermann, Faraz Hach, Syed Haider, Natsuko Hama, Freddie C. Hamdy, Anne Hamilton, Mark P. Hamilton, Leng Han, George B. Hanna, Martin Hansmann, Nicholas J. Haradhvala, Olivier Harismendy, Ivon Harliwong, Arif O. Harmanci, Eoghan Harrington, Takanori Hasegawa, David Haussler, Steve Hawkins, Shinya Hayami, Shuto Hayashi, D. Neil Hayes, Stephen J. Hayes, Nicholas K. Hayward, Steven Hazell, Yao He, Allison P. Heath, Simon C. Heath, David Hedley, Apurva M. Hegde, David I. Heiman, Michael C. Heinold, Zachary Heins, Lawrence E. Heisler, Eva Hellstrom-Lindberg, Mohamed Helmy, Seong Gu Heo, Austin J. Hepperla, José María Heredia-Genestar, Carl Herrmann, Peter Hersey, Julian M. Hess, Holmfridur Hilmarsdottir, Jonathan Hinton, Satoshi Hirano, Nobuyoshi Hiraoka, Katherine A. Hoadley, Asger Hobolth, Ermin Hodzic, Jessica I. Hoell, Steve Hoffmann, Oliver Hofmann, Andrea Holbrook, Aliaksei Z. Holik, Michael A. Hollingsworth, Oliver Holmes, Robert A. Holt, Chen Hong, Eun Pyo Hong, Jongwhi H. Hong, Gerrit K. Hooijer, Henrik Hornshøj, Fumie Hosoda, Yong Hou, Volker Hovestadt, William Howat, Alan P. Hoyle, Ralph H. Hruban, Jianhong Hu, Taobo Hu, Xing Hua, Kuan-lin Huang, Mei Huang, Mi Ni Huang, Vincent Huang, Yi Huang, Wolfgang Huber, Thomas J. Hudson, Michael Hummel, Jillian A. Hung, David Huntsman, Ted R. Hupp, Jason Huse, Matthew R. Huska, Barbara Hutter, Carolyn M. Hutter, Daniel Hübschmann, Christine A. Iacobuzio-Donahue, Charles David Imbusch, Marcin Imielinski, Seiya Imoto, William B. Isaacs, Keren Isaev, Shumpei Ishikawa, Murat Iskar, S. M. Ashiqul Islam, Michael Ittmann, Sinisa Ivkovic, Jose M. G. Izarzugaza, Jocelyne Jacquemier, Valerie Jakrot, Nigel B. Jamieson, Gun Ho Jang, Se Jin Jang, Joy C. Jayaseelan, Reyka Jayasinghe, Stuart R. Jefferys, Karine Jegalian, Jennifer L. Jennings, Seung-Hyup Jeon, Lara Jerman, Yuan Ji, Wei Jiao, Peter A. Johansson, Amber L. Johns, Jeremy Johns, Rory Johnson, Todd A. Johnson, Clemency Jolly, Yann Joly, Jon G. Jonasson, Corbin D. Jones, David R. Jones, David T. W. Jones, Nic Jones, Steven J. M. Jones, Jos Jonkers, Young Seok Ju, Hartmut Juhl, Jongsun Jung, Malene Juul, Randi Istrup Juul, Sissel Juul, Natalie Jäger, Rolf Kabbe, Andre Kahles, Abdullah Kahraman, Vera B. Kaiser, Hojabr Kakavand, Sangeetha Kalimuthu, Christof von Kalle, Koo Jeong Kang, Katalin Karaszi, Beth Karlan, Rosa Karlić, Dennis Karsch, Katayoon Kasaian, Karin S. Kassahn, Hitoshi Katai, Mamoru Kato, Hiroto Katoh, Yoshiiku Kawakami, Jonathan D. Kay, Stephen H. Kazakoff, Marat D. Kazanov, Maria Keays, Electron Kebebew, Richard F. Kefford, Manolis Kellis, James G. Kench, Catherine J. Kennedy, Jules N. A. Kerssemakers, David Khoo, Vincent Khoo, Narong Khuntikeo, Ekta Khurana, Helena Kilpinen, Hark Kyun Kim, Hyung-Lae Kim, Hyung-Yong Kim, Hyunghwan Kim, Jaegil Kim, Jihoon Kim, Jong K. Kim, Youngwook Kim, Tari A. King, Wolfram Klapper, Kortine Kleinheinz, Leszek J. Klimczak, Stian Knappskog, Michael Kneba, Bartha M. Knoppers, Youngil Koh, Jan Komorowski, Daisuke Komura, Mitsuhiro Komura, Gu Kong, Marcel Kool, Jan O. Korbel, Viktoriya Korchina, Andrey Korshunov, Michael Koscher, Roelof Koster, Zsofia Kote-Jarai, Antonios Koures, Milena Kovacevic, Barbara Kremeyer, Helene Kretzmer, Markus Kreuz, Savitri Krishnamurthy, Dieter Kube, Kiran Kumar, Pardeep Kumar, Sushant Kumar, Yogesh Kumar, Ritika Kundra, Kirsten Kübler, Ralf Küppers, Jesper Lagergren, Phillip H. Lai, Peter W. Laird, Sunil R. Lakhani, Christopher M. Lalansingh, Emilie Lalonde, Fabien C. Lamaze, Adam Lambert, Eric Lander, Pablo Landgraf, Luca Landoni, Anita Langerød, Andrés Lanzós, Denis Larsimont, Erik Larsson, Mark Lathrop, Loretta M. S. Lau, Chris Lawerenz, Rita T. Lawlor, Michael S. Lawrence, Alexander J. Lazar, Ana Mijalkovic Lazic, Xuan Le, Darlene Lee, Donghoon Lee, Eunjung Alice Lee, Hee Jin Lee, Jake June-Koo Lee, Jeong-Yeon Lee, Juhee Lee, Ming Ta Michael Lee, Henry Lee-Six, Kjong-Van Lehmann, Hans Lehrach, Dido Lenze, Conrad R. Leonard, Daniel A. Leongamornlert, Ignaty Leshchiner, Louis Letourneau, Ivica Letunic, Douglas A. Levine, Lora Lewis, Tim Ley, Chang Li, Constance H. Li, Haiyan Irene Li, Jun Li, Lin Li, Shantao Li, Siliang Li, Xiaobo Li, Xiaotong Li, Xinyue Li, Yilong Li, Han Liang, Sheng-Ben Liang, Peter Lichter, Pei Lin, Ziao Lin, W. M. Linehan, Ole Christian Lingjærde, Dongbing Liu, Eric Minwei Liu, Fei-Fei Fei Liu, Fenglin Liu, Jia Liu, Xingmin Liu, Julie Livingstone, Dimitri Livitz, Naomi Livni, Lucas Lochovsky, Markus Loeffler, Georgina V. Long, Armando Lopez-Guillermo, Shaoke Lou, David N. Louis, Laurence B. Lovat, Yiling Lu, Yong-Jie Lu, Youyong Lu, Claudio Luchini, Ilinca Lungu, Xuemei Luo, Hayley J. Luxton, Andy G. Lynch, Lisa Lype, Cristina López, Carlos López-Otín, Eric Z. Ma, Yussanne Ma, Gaetan MacGrogan, Shona MacRae, Geoff Macintyre, Tobias Madsen, Kazuhiro Maejima, Andrea Mafficini, Dennis T. Maglinte, Arindam Maitra, Partha P. Majumder, Luca Malcovati, Salem Malikic, Giuseppe Malleo, Graham J. Mann, Luisa Mantovani-Löffler, Kathleen Marchal, Giovanni Marchegiani, Elaine R. Mardis, Adam A. Margolin, Maximillian G. Marin, Florian Markowetz, Julia Markowski, Jeffrey Marks, Tomas Marques-Bonet, Marco A. Marra, Luke Marsden, John W. M. Martens, Sancha Martin, Jose I. Martin-Subero, Iñigo Martincorena, Alexander Martinez-Fundichely, Yosef E. Maruvka, R. Jay Mashl, Charlie E. Massie, Thomas J. Matthew, Lucy Matthews, Erik Mayer, Simon Mayes, Michael Mayo, Faridah Mbabaali, Karen McCune, Ultan McDermott, Patrick D. McGillivray, Michael D. McLellan, John D. McPherson, John R. McPherson, Treasa A. McPherson, Samuel R. Meier, Alice Meng, Shaowu Meng, Andrew Menzies, Neil D. Merrett, Sue Merson, Matthew Meyerson, William Meyerson, Piotr A. Mieczkowski, George L. Mihaiescu, Sanja Mijalkovic, Tom Mikkelsen, Michele Milella, Linda Mileshkin, Christopher A. Miller, David K. Miller, Jessica K. Miller, Gordon B. Mills, Ana Milovanovic, Sarah Minner, Marco Miotto, Gisela Mir Arnau, Lisa Mirabello, Chris Mitchell, Thomas J. Mitchell, Satoru Miyano, Naoki Miyoshi, Shinichi Mizuno, Fruzsina Molnár-Gábor, Malcolm J. Moore, Richard A. Moore, Sandro Morganella, Quaid D. Morris, Carl Morrison, Lisle E. Mose, Catherine D. Moser, Ferran Muiños, Loris Mularoni, Andrew J. Mungall, Karen Mungall, Elizabeth A. Musgrove, Ville Mustonen, David Mutch, Francesc Muyas, Donna M. Muzny, Alfonso Muñoz, Jerome Myers, Ola Myklebost, Peter Möller, Genta Nagae, Adnan M. Nagrial, Hardeep K. Nahal-Bose, Hitoshi Nakagama, Hidewaki Nakagawa, Hiromi Nakamura, Toru Nakamura, Kaoru Nakano, Tannistha Nandi, Jyoti Nangalia, Mia Nastic, Arcadi Navarro, Fabio C. P. Navarro, David E. Neal, Gerd Nettekoven, Felicity Newell, Steven J. Newhouse, Yulia Newton, Alvin Wei Tian Ng, Anthony Ng, Jonathan Nicholson, David Nicol, Yongzhan Nie, G. Petur Nielsen, Morten Muhlig Nielsen, Serena Nik-Zainal, Michael S. Noble, Katia Nones, Paul A. Northcott, Faiyaz Notta, Brian D. O’Connor, Peter O’Donnell, Maria O’Donovan, Sarah O’Meara, Brian Patrick O’Neill, J. Robert O’Neill, David Ocana, Angelica Ochoa, Layla Oesper, Christopher Ogden, Hideki Ohdan, Kazuhiro Ohi, Lucila Ohno-Machado, Karin A. Oien, Akinyemi I. Ojesina, Hidenori Ojima, Takuji Okusaka, Larsson Omberg, Choon Kiat Ong, Stephan Ossowski, German Ott, B. F. Francis Ouellette, Christine P’ng, Marta Paczkowska, Salvatore Paiella, Chawalit Pairojkul, Marina Pajic, Qiang Pan-Hammarström, Elli Papaemmanuil, Irene Papatheodorou, Nagarajan Paramasivam, Ji Wan Park, Joong-Won Park, Keunchil Park, Kiejung Park, Peter J. Park, Joel S. Parker, Simon L. Parsons, Harvey Pass, Danielle Pasternack, Alessandro Pastore, Ann-Marie Patch, Iris Pauporté, Antonio Pea, John V. Pearson, Chandra Sekhar Pedamallu, Jakob Skou Pedersen, Paolo Pederzoli, Martin Peifer, Nathan A. Pennell, Charles M. Perou, Marc D. Perry, Gloria M. Petersen, Myron Peto, Nicholas Petrelli, Robert Petryszak, Stefan M. Pfister, Mark Phillips, Oriol Pich, Hilda A. Pickett, Todd D. Pihl, Nischalan Pillay, Sarah Pinder, Mark Pinese, Andreia V. Pinho, Esa Pitkänen, Xavier Pivot, Elena Piñeiro-Yáñez, Laura Planko, Christoph Plass, Paz Polak, Tirso Pons, Irinel Popescu, Olga Potapova, Aparna Prasad, Shaun R. Preston, Manuel Prinz, Antonia L. Pritchard, Stephenie D. Prokopec, Elena Provenzano, Xose S. Puente, Sonia Puig, Montserrat Puiggròs, Sergio Pulido-Tamayo, Gulietta M. Pupo, Colin A. Purdie, Michael C. Quinn, Raquel Rabionet, Janet S. Rader, Bernhard Radlwimmer, Petar Radovic, Benjamin Raeder, Keiran M. Raine, Manasa Ramakrishna, Kamna Ramakrishnan, Suresh Ramalingam, Benjamin J. Raphael, W. Kimryn Rathmell, Tobias Rausch, Guido Reifenberger, Jüri Reimand, Jorge Reis-Filho, Victor Reuter, Iker Reyes-Salazar, Matthew A. Reyna, Sheila M. Reynolds, Esther Rheinbay, Yasser Riazalhosseini, Andrea L. Richardson, Julia Richter, Matthew Ringel, Markus Ringnér, Yasushi Rino, Karsten Rippe, Jeffrey Roach, Lewis R. Roberts, Nicola D. Roberts, Steven A. Roberts, A. Gordon Robertson, Alan J. Robertson, Javier Bartolomé Rodriguez, Bernardo Rodriguez-Martin, F. Germán Rodríguez-González, Michael H. A. Roehrl, Marius Rohde, Hirofumi Rokutan, Gilles Romieu, Ilse Rooman, Tom Roques, Daniel Rosebrock, Mara Rosenberg, Philip C. Rosenstiel, Andreas Rosenwald, Edward W. Rowe, Romina Royo, Steven G. Rozen, Yulia Rubanova, Mark A. Rubin, Carlota Rubio-Perez, Vasilisa A. Rudneva, Borislav C. Rusev, Andrea Ruzzenente, Gunnar Rätsch, Radhakrishnan Sabarinathan, Veronica Y. Sabelnykova, Sara Sadeghi, S. Cenk Sahinalp, Natalie Saini, Mihoko Saito-Adachi, Gordon Saksena, Adriana Salcedo, Roberto Salgado, Leonidas Salichos, Richard Sallari, Charles Saller, Roberto Salvia, Michelle Sam, Jaswinder S. Samra, Francisco Sanchez-Vega, Chris Sander, Grant Sanders, Rajiv Sarin, Iman Sarrafi, Aya Sasaki-Oku, Torill Sauer, Guido Sauter, Robyn P. M. Saw, Maria Scardoni, Christopher J. Scarlett, Aldo Scarpa, Ghislaine Scelo, Dirk Schadendorf, Jacqueline E. Schein, Markus B. Schilhabel, Matthias Schlesner, Thorsten Schlomm, Heather K. Schmidt, Sarah-Jane Schramm, Stefan Schreiber, Nikolaus Schultz, Steven E. Schumacher, Roland F. Schwarz, Richard A. Scolyer, David Scott, Ralph Scully, Raja Seethala, Ayellet V. Segre, Iris Selander, Colin A. Semple, Yasin Senbabaoglu, Subhajit Sengupta, Elisabetta Sereni, Stefano Serra, Dennis C. Sgroi, Mark Shackleton, Nimish C. Shah, Sagedeh Shahabi, Catherine A. Shang, Ping Shang, Ofer Shapira, Troy Shelton, Ciyue Shen, Hui Shen, Rebecca Shepherd, Ruian Shi, Yan Shi, Yu-Jia Shiah, Tatsuhiro Shibata, Juliann Shih, Eigo Shimizu, Kiyo Shimizu, Seung Jun Shin, Yuichi Shiraishi, Tal Shmaya, Ilya Shmulevich, Solomon I. Shorser, Charles Short, Raunak Shrestha, Suyash S. Shringarpure, Craig Shriver, Shimin Shuai, Nikos Sidiropoulos, Reiner Siebert, Anieta M. Sieuwerts, Lina Sieverling, Sabina Signoretti, Katarzyna O. Sikora, Michele Simbolo, Ronald Simon, Janae V. Simons, Jared T. Simpson, Peter T. Simpson, Samuel Singer, Nasa Sinnott-Armstrong, Payal Sipahimalani, Tara J. Skelly, Marcel Smid, Jaclyn Smith, Karen Smith-McCune, Nicholas D. Socci, Heidi J. Sofia, Matthew G. Soloway, Lei Song, Anil K. Sood, Sharmila Sothi, Christos Sotiriou, Cameron M. Soulette, Paul N. Span, Paul T. Spellman, Nicola Sperandio, Andrew J. Spillane, Oliver Spiro, Jonathan Spring, Johan Staaf, Peter F. Stadler, Peter Staib, Stefan G. Stark, Lucy Stebbings, Ólafur Andri Stefánsson, Oliver Stegle, Lincoln D. Stein, Alasdair Stenhouse, Chip Stewart, Stephan Stilgenbauer, Miranda D. Stobbe, Michael R. Stratton, Jonathan R. Stretch, Adam J. Struck, Joshua M. Stuart, Henk G. Stunnenberg, Hong Su, Xiaoping Su, Ren X. Sun, Stephanie Sungalee, Hana Susak, Akihiro Suzuki, Fred Sweep, Monika Szczepanowski, Holger Sültmann, Takashi Yugawa, Angela Tam, David Tamborero, Benita Kiat Tee Tan, Donghui Tan, Patrick Tan, Hiroko Tanaka, Hirokazu Taniguchi, Tomas J. Tanskanen, Maxime Tarabichi, Roy Tarnuzzer, Patrick Tarpey, Morgan L. Taschuk, Kenji Tatsuno, Simon Tavaré, Darrin F. Taylor, Amaro Taylor-Weiner, Jon W. Teague, Bin Tean Teh, Varsha Tembe, Javier Temes, Kevin Thai, Sarah P. Thayer, Nina Thiessen, Gilles Thomas, Sarah Thomas, Alan Thompson, Alastair M. Thompson, John F. F. Thompson, R. Houston Thompson, Heather Thorne, Leigh B. Thorne, Adrian Thorogood, Grace Tiao, Nebojsa Tijanic, Lee E. Timms, Roberto Tirabosco, Marta Tojo, Stefania Tommasi, Christopher W. Toon, Umut H. Toprak, David Torrents, Giampaolo Tortora, Jörg Tost, Yasushi Totoki, David Townend, Nadia Traficante, Isabelle Treilleux, Jean-Rémi Trotta, Lorenz H. P. Trümper, Ming Tsao, Tatsuhiko Tsunoda, Jose M. C. Tubio, Olga Tucker, Richard Turkington, Daniel J. Turner, Andrew Tutt, Masaki Ueno, Naoto T. Ueno, Christopher Umbricht, Husen M. Umer, Timothy J. Underwood, Lara Urban, Tomoko Urushidate, Tetsuo Ushiku, Liis Uusküla-Reimand, Alfonso Valencia, David J. Van Den Berg, Steven Van Laere, Peter Van Loo, Erwin G. Van Meir, Gert G. Van den Eynden, Theodorus Van der Kwast, Naveen Vasudev, Miguel Vazquez, Ravikiran Vedururu, Umadevi Veluvolu, Shankar Vembu, Lieven P. C. Verbeke, Peter Vermeulen, Clare Verrill, Alain Viari, David Vicente, Caterina Vicentini, K. VijayRaghavan, Juris Viksna, Ricardo E. Vilain, Izar Villasante, Anne Vincent-Salomon, Tapio Visakorpi, Douglas Voet, Paresh Vyas, Ignacio Vázquez-García, Nick M. Waddell, Nicola Waddell, Claes Wadelius, Lina Wadi, Rabea Wagener, Jeremiah A. Wala, Jian Wang, Jiayin Wang, Linghua Wang, Qi Wang, Wenyi Wang, Yumeng Wang, Zhining Wang, Paul M. Waring, Hans-Jörg Warnatz, Jonathan Warrell, Anne Y. Warren, Sebastian M. Waszak, David C. Wedge, Dieter Weichenhan, Paul Weinberger, John N. Weinstein, Joachim Weischenfeldt, Daniel J. Weisenberger, Ian Welch, Michael C. Wendl, Johannes Werner, Justin P. Whalley, David A. Wheeler, Hayley C. Whitaker, Dennis Wigle, Matthew D. Wilkerson, Ashley Williams, James S. Wilmott, Gavin W. Wilson, Julie M. Wilson, Richard K. Wilson, Boris Winterhoff, Jeffrey A. Wintersinger, Maciej Wiznerowicz, Stephan Wolf, Bernice H. Wong, Tina Wong, Winghing Wong, Youngchoon Woo, Scott Wood, Bradly G. Wouters, Adam J. Wright, Derek W. Wright, Mark H. Wright, Chin-Lee Wu, Dai-Ying Wu, Guanming Wu, Jianmin Wu, Kui Wu, Yang Wu, Zhenggang Wu, Liu Xi, Tian Xia, Qian Xiang, Xiao Xiao, Rui Xing, Heng Xiong, Qinying Xu, Yanxun Xu, Hong Xue, Shinichi Yachida, Sergei Yakneen, Rui Yamaguchi, Takafumi N. Yamaguchi, Masakazu Yamamoto, Shogo Yamamoto, Hiroki Yamaue, Fan Yang, Huanming Yang, Jean Y. Yang, Liming Yang, Lixing Yang, Shanlin Yang, Tsun-Po Yang, Yang Yang, Xiaotong Yao, Marie-Laure Yaspo, Lucy Yates, Christina Yau, Chen Ye, Kai Ye, Venkata D. Yellapantula, Christopher J. Yoon, Sung-Soo Yoon, Fouad Yousif, Jun Yu, Kaixian Yu, Willie Yu, Yingyan Yu, Ke Yuan, Yuan Yuan, Denis Yuen, Christina K. Yung, Olga Zaikova, Jorge Zamora, Marc Zapatka, Jean C. Zenklusen, Thorsten Zenz, Nikolajs Zeps, Cheng-Zhong Zhang, Fan Zhang, Hailei Zhang, Hongwei Zhang, Hongxin Zhang, Jiashan Zhang, Jing Zhang, Junjun Zhang, Xiuqing Zhang, Xuanping Zhang, Yan Zhang, Zemin Zhang, Zhongming Zhao, Liangtao Zheng, Xiuqing Zheng, Wanding Zhou, Yong Zhou, Bin Zhu, Hongtu Zhu, Jingchun Zhu, Shida Zhu, Lihua Zou, Xueqing Zou, Anna deFazio, Nicholas van As, Carolien H. M. van Deurzen, Marc J. van de Vijver, L. van’t Veer, Christian von Mering

**Affiliations:** 1 Department of Cellular and Molecular Medicine, Department of Bioengineering, Moores Cancer Center, University of California, San Diego, CA USA; 2 Broad Institute of MIT and Harvard, Cambridge, MA USA; 3 Center for Cancer Research, Massachusetts General Hospital, Boston, MA USA; 4 Programme in Cancer & Stem Cell Biology, Duke-NUS Medical School, Singapore, Singapore; 5 Centre for Computational Biology, Duke-NUS Medical School, Singapore, Singapore; 6 Human Genome Sequencing Center, Baylor College of Medicine, Houston, TX USA; 7 Dan L. Duncan Cancer Center, Baylor College of Medicine, Houston, TX USA; 8 Genome Integrity and Structural Biology Laboratory, National Institute of Environmental Health Sciences (NIEHS), Durham, NC USA; 9 Institute for Research in Biomedicine (IRB Barcelona), The Barcelona Institute of Science and Technology, Barcelona, Spain; 10 Research Program on Biomedical Informatics, Universitat Pompeu Fabra, Barcelona, Spain; 11 Institució Catalana de Recerca i Estudis Avançats (ICREA), Barcelona, Spain; 12 Integrative Bioinformatics Support Group, National Institute of Environmental Health Sciences (NIEHS), Durham, NC USA; 13 Wellcome Sanger Institute, Hinxton, UK; 14 National Centre for Biological Sciences, Tata Institute of Fundamental Research, Bangalore, India; 15 Institute for Research in Biomedicine (IRB Barcelona), The Barcelona Institute of Science and Technology, Barcelona, Spain; 16 Department of Molecular and Human Genetics, Baylor College of Medicine, Houston, TX USA; 17 Department of Computer Science, University of Helsinki, Helsinki, Finland; 18 Organismal and Evolutionary Biology Research Programme, University of Helsinki, Helsinki, Finland; 19 Institute of Biotechnology, University of Helsinki, Helsinki, Finland; 21 Department of Pathology, Massachusetts General Hospital, Boston, MA USA; 22 Harvard Medical School, Boston, MA USA; 23 SingHealth, Duke-NUS Institute of Precision Medicine, National Heart Centre Singapore, Singapore, Singapore; 25 Department of Medical Biophysics, University of Toronto, Toronto, Ontario Canada; 26 University of California Los Angeles, Los Angeles, CA USA; 27 Computational Biology Program, Ontario Institute for Cancer Research, Toronto, Ontario Canada; 28 Department of Pharmacology, University of Toronto, Toronto, Ontario Canada; 29 Department of Biochemistry, Microbiology and Immunology, Faculty of Medicine, University of Ottawa, Ottawa, Ontario Canada; 30 RIKEN Center for Integrative Medical Sciences, Yokohama, Japan; 31 A. A. Kharkevich Institute of Information Transmission Problems, Moscow, Russia; 32 Dmitry Rogachev National Research Center of Pediatric Hematology, Oncology and Immunology, Moscow, Russia; 33 Skolkovo Institute of Science and Technology, Moscow, Russia; 34 Laboratory for Medical Science Mathematics, RIKEN Center for Integrative Medical Sciences, Yokohama, Japan; 35 Massachusetts General Hospital, Boston, MA USA; 36 School of Molecular Biosciences, Washington State University, Pullman, WA USA; 37 Center for Reproductive Biology, Washington State University, Pullman, WA USA; 38 Laboratory of Molecular Medicine, Human Genome Center, The Institute of Medical Science, The University of Tokyo, Tokyo, Japan; 39 Division of Cancer Genomics, National Cancer Center Research Institute, Tokyo, Japan; 40 The Institute of Medical Science, The University of Tokyo, Tokyo, Japan; 41 Cancer Science Institute of Singapore, National University of Singapore, Singapore, Singapore; 42 Institute of Molecular and Cell Biology, Singapore, Singapore; 43 Laboratory of Cancer Epigenome, Division of Medical Science, National Cancer Centre Singapore, Singapore, Singapore; 44 Department of Epidemiology and Biostatistics, Memorial Sloan Kettering Cancer Center, New York, NY USA; 45 Department of Statistics, Columbia University, New York, NY USA; 46 Department of Applied Mathematics and Theoretical Physics, Centre for Mathematical Sciences, University of Cambridge, Cambridge, UK; 200 Applied Tumor Genomics Research Program, Research Programs Unit, University of Helsinki, Helsinki, Finland; 201 Wellcome Sanger Institute, Wellcome Genome Campus, Hinxton, UK; 202 Memorial Sloan Kettering Cancer Center, New York, NY USA; 203 Genome Science Division, Research Center for Advanced Science and Technology, University of Tokyo, Tokyo, Japan; 204 Department of Surgery, University of Chicago, Chicago, IL USA; 205 Department of Surgery, Division of Hepatobiliary and Pancreatic Surgery, School of Medicine, Keimyung University Dongsan Medical Center, Daegu, South Korea; 206 Department of Oncology, Gil Medical Center, Gachon University, Incheon, South Korea; 207 Hiroshima University, Hiroshima, Japan; 208 Department of Bioinformatics and Computational Biology, The University of Texas MD Anderson Cancer Center, Houston, TX USA; 209 University of Texas MD Anderson Cancer Center, Houston, TX USA; 210 King Faisal Specialist Hospital and Research Centre, Al Maather, Riyadh, Saudi Arabia; 211 Bioinformatics Unit, Spanish National Cancer Research Centre (CNIO), Madrid, Spain; 212 Bioinformatics Core Facility, University Medical Center Hamburg, Hamburg, Germany; 213 Heinrich Pette Institute, Leibniz Institute for Experimental Virology, Hamburg, Germany; 214 Ontario Tumour Bank, Ontario Institute for Cancer Research, Toronto, ON Canada; 215 Department of Pathology, The University of Texas MD Anderson Cancer Center, Houston, TX USA; 216 Laboratory of Pathology, Center for Cancer Research, National Cancer Institute, Bethesda, MD USA; 217 Department of Cellular and Molecular Medicine and Department of Bioengineering, University of California San Diego, La Jolla, CA USA; 218 UC San Diego Moores Cancer Center, San Diego, CA USA; 219 Canada’s Michael Smith Genome Sciences Centre, BC Cancer, Vancouver, BC Canada; 220 Sir Peter MacCallum Department of Oncology, Peter MacCallum Cancer Centre, University of Melbourne, Melbourne, VIC Australia; 221 Centre for Research in Molecular Medicine and Chronic Diseases (CiMUS), Universidade de Santiago de Compostela, Santiago de Compostela, Spain; 222 Department of Zoology, Genetics and Physical Anthropology, (CiMUS), Universidade de Santiago de Compostela, Santiago de Compostela, Spain; 223 The Biomedical Research Centre (CINBIO), Universidade de Vigo, Vigo, Spain; 224 Royal National Orthopaedic Hospital - Bolsover, London, UK; 225 Department of Genomic Medicine, The University of Texas MD Anderson Cancer Center, Houston, TX USA; 226 Quantitative and Computational Biosciences Graduate Program, Baylor College of Medicine, Houston, TX USA; 227 The Jackson Laboratory for Genomic Medicine, Farmington, CT USA; 228 Genome Informatics Program, Ontario Institute for Cancer Research, Toronto, ON Canada; 229 Institute of Human Genetics, Christian-Albrechts-University, Kiel, Germany; 230 Institute of Human Genetics, Ulm University and Ulm University Medical Center, Ulm, Germany; 231 Queensland Centre for Medical Genomics, Institute for Molecular Bioscience, University of Queensland, St. Lucia, Brisbane, QLD Australia; 232 Salford Royal NHS Foundation Trust, Salford, UK; 233 Department of Surgery, Pancreas Institute, University and Hospital Trust of Verona, Verona, Italy; 234 Molecular and Medical Genetics, OHSU Knight Cancer Institute, Oregon Health and Science University, Portland, OR USA; 235 Department of Molecular Oncology, BC Cancer Research Centre, Vancouver, BC Canada; 236 The McDonnell Genome Institute at Washington University, St. Louis, MO USA; 237 University College London, London, UK; 238 Division of Cancer Genomics, National Cancer Center Research Institute, National Cancer Center, Tokyo, Japan; 239 DLR Project Management Agency, Bonn, Germany; 240 Tokyo Women’s Medical University, Tokyo, Japan; 241 Center for Molecular Oncology, Memorial Sloan Kettering Cancer Center, New York, NY USA; 242 Los Alamos National Laboratory, Los Alamos, NM USA; 243 Department of Pathology, University Health Network, Toronto General Hospital, Toronto, ON Canada; 244 Nottingham University Hospitals NHS Trust, Nottingham, UK; 245 Epigenomics and Cancer Risk Factors, German Cancer Research Center (DKFZ), Heidelberg, Germany; 246 Computational Biology Program, Ontario Institute for Cancer Research, Toronto, ON Canada; 247 Department of Molecular Genetics, University of Toronto, Toronto, ON Canada; 248 Vector Institute, Toronto, ON Canada; 249 Hematopathology Section, Institute of Pathology, Christian-Albrechts-University, Kiel, Germany; 250 Department of Pathology and Laboratory Medicine, School of Medicine, University of North Carolina at Chapel Hill, Chapel Hill, NC USA; 251 Department of Cancer Genetics, Institute for Cancer Research, Oslo University Hospital, The Norwegian Radium Hospital, Oslo, Norway; 252 Pathology, Hospital Clinic, Institut d’Investigacions Biomèdiques August Pi i Sunyer (IDIBAPS), University of Barcelona, Barcelona, Spain; 253 Department of Veterinary Medicine, Transmissible Cancer Group, University of Cambridge, Cambridge, UK; 254 Alvin J. Siteman Cancer Center, Washington University School of Medicine, St. Louis, MO USA; 255 Wolfson Wohl Cancer Research Centre, Institute of Cancer Sciences, University of Glasgow, Glasgow, UK; 256 Lineberger Comprehensive Cancer Center, University of North Carolina at Chapel Hill, Chapel Hill, NC USA; 257 Broad Institute of MIT and Harvard, Cambridge, MA USA; 258 Dana-Farber/Boston Children’s Cancer and Blood Disorders Center, Boston, MA USA; 259 Department of Pediatrics, Harvard Medical School, Boston, MA USA; 260 Leeds Institute of Medical Research @ St. James’s, University of Leeds, St. James’s University Hospital, Leeds, UK; 261 Department of Pathology and Diagnostics, University and Hospital Trust of Verona, Verona, Italy; 262 Department of Surgery, Princess Alexandra Hospital, Brisbane, QLD Australia; 263 Surgical Oncology Group, Diamantina Institute, University of Queensland, Brisbane, QLD Australia; 264 Department of Population and Quantitative Health Sciences, Case Western Reserve University School of Medicine, Cleveland, OH USA; 265 Research Health Analytics and Informatics, University Hospitals Cleveland Medical Center, Cleveland, OH USA; 266 Gloucester Royal Hospital, Gloucester, UK; 267 European Molecular Biology Laboratory, European Bioinformatics Institute (EMBL-EBI), Cambridge, UK; 268 Diagnostic Development, Ontario Institute for Cancer Research, Toronto, ON Canada; 269 Barcelona Supercomputing Center (BSC), Barcelona, Spain; 270 Arnie Charbonneau Cancer Institute, University of Calgary, Calgary, AB Canada; 271 Departments of Surgery and Oncology, University of Calgary, Calgary, AB Canada; 272 Department of Pathology, Oslo University Hospital, The Norwegian Radium Hospital, Oslo, Norway; 273 PanCuRx Translational Research Initiative, Ontario Institute for Cancer Research, Toronto, ON Canada; 274 Department of Oncology, Sidney Kimmel Comprehensive Cancer Center at Johns Hopkins University School of Medicine, Baltimore, MD USA; 275 University Hospital Southampton NHS Foundation Trust, Southampton, UK; 276 Royal Stoke University Hospital, Stoke-on-Trent, UK; 277 Genome Sequence Informatics, Ontario Institute for Cancer Research, Toronto, ON Canada; 278 Human Longevity Inc, San Diego, CA USA; 279 Olivia Newton-John Cancer Research Institute, La Trobe University, Heidelberg, VIC Australia; 280 Computer Network Information Center, Chinese Academy of Sciences, Beijing, China; 281 Genome Canada, Ottawa, ON Canada; 282 CNAG-CRG, Centre for Genomic Regulation (CRG), Barcelona Institute of Science and Technology (BIST), Barcelona, Spain; 283 Universitat Pompeu Fabra (UPF), Barcelona, Spain; 284 Buck Institute for Research on Aging, Novato, CA USA; 285 Duke University Medical Center, Durham, NC USA; 286 Department of Human Genetics, Hannover Medical School, Hannover, Germany; 287 Center for Bioinformatics and Functional Genomics, Cedars-Sinai Medical Center, Los Angeles, CA USA; 288 Department of Biomedical Sciences, Cedars-Sinai Medical Center, Los Angeles, CA USA; 289 The Hebrew University Faculty of Medicine, Jerusalem, Israel; 290 Barts Cancer Institute, Barts and the London School of Medicine and Dentistry, Queen Mary University of London, London, UK; 291 Department of Computer Science, Bioinformatics Group, University of Leipzig, Leipzig, Germany; 292 Interdisciplinary Center for Bioinformatics, University of Leipzig, Leipzig, Germany; 293 Transcriptome Bioinformatics, LIFE Research Center for Civilization Diseases, University of Leipzig, Leipzig, Germany; 294 Department of Medical Oncology, Dana-Farber Cancer Institute, Boston, MA USA; 295 Department of Cancer Biology, Dana-Farber Cancer Institute, Boston, MA USA; 296 Harvard Medical School, Boston, MA USA; 297 USC Norris Comprehensive Cancer Center, University of Southern California, Los Angeles, CA USA; 298 Department of Diagnostics and Public Health, University and Hospital Trust of Verona, Verona, Italy; 299 Department of Mathematics, Aarhus University, Aarhus, Denmark; 300 Department of Molecular Medicine (MOMA), Aarhus University Hospital, Aarhus N, Denmark; 301 Instituto Carlos Slim de la Salud, Mexico City, Mexico; 302 Department of Medical Biophysics, University of Toronto, Toronto, ON Canada; 303 Cancer Division, Garvan Institute of Medical Research, Kinghorn Cancer Centre, University of New South Wales (UNSW Sydney), Sydney, NSW Australia; 304 South Western Sydney Clinical School, Faculty of Medicine, University of New South Wales (UNSW Sydney), Liverpool, NSW Australia; 305 West of Scotland Pancreatic Unit, Glasgow Royal Infirmary, Glasgow, UK; 306 Center for Digital Health, Berlin Institute of Health and Charitè - Universitätsmedizin Berlin, Berlin, Germany; 307 Heidelberg Center for Personalized Oncology (DKFZ-HIPO), German Cancer Research Center (DKFZ), Heidelberg, Germany; 308 The Preston Robert Tisch Brain Tumor Center, Duke University Medical Center, Durham, NC USA; 309 Massachusetts General Hospital, Boston, MA USA; 310 National Institute of Biomedical Genomics, Kalyani, West Bengal India; 311 Institute of Clinical Medicine and Institute of Oral Biology, University of Oslo, Oslo, Norway; 312 University of North Carolina at Chapel Hill, Chapel Hill, NC USA; 313 ARC-Net Centre for Applied Research on Cancer, University and Hospital Trust of Verona, Verona, Italy; 314 The Institute of Cancer Research, London, UK; 315 Centre for Computational Biology, Duke-NUS Medical School, Singapore, Singapore; 316 Programme in Cancer and Stem Cell Biology, Duke-NUS Medical School, Singapore, Singapore; 317 Division of Oncology and Pathology, Department of Clinical Sciences Lund, Lund University, Lund, Sweden; 318 Department of Pediatric Oncology, Hematology and Clinical Immunology, Heinrich-Heine-University, Düsseldorf, Germany; 319 Laboratory for Medical Science Mathematics, RIKEN Center for Integrative Medical Sciences, Yokohama, Japan; 320 RIKEN Center for Integrative Medical Sciences, Yokohama, Japan; 321 Department of Internal Medicine/Hematology, Friedrich-Ebert-Hospital, Neumünster, Germany; 322 Departments of Dermatology and Pathology, Yale University, New Haven, CT USA; 323 Centre for Genomic Regulation (CRG), The Barcelona Institute of Science and Technology, Barcelona, Spain; 324 Radcliffe Department of Medicine, University of Oxford, Oxford, UK; 325 Canadian Center for Computational Genomics, McGill University, Montreal, QC Canada; 326 Department of Human Genetics, McGill University, Montreal, QC Canada; 327 Department of Human Genetics, University of California Los Angeles, Los Angeles, CA USA; 328 Department of Pharmacology, University of Toronto, Toronto, ON Canada; 329 Faculty of Medicine and Health Technology, Tampere University and Tays Cancer Center, Tampere University Hospital, Tampere, Finland; 330 Haematology, Leeds Teaching Hospitals NHS Trust, Leeds, UK; 331 Translational Research and Innovation, Centre Léon Bérard, Lyon, France; 332 Fox Chase Cancer Center, Philadelphia, PA USA; 333 International Agency for Research on Cancer, World Health Organization, Lyon, France; 334 Earlham Institute, Norwich, UK; 335 Norwich Medical School, University of East Anglia, Norwich, UK; 336 Department of Molecular Biology, Faculty of Science, Radboud Institute for Molecular Life Sciences, Radboud University, Nijmegen, HB The Netherlands; 337 CRUK Manchester Institute and Centre, Manchester, UK; 338 Department of Radiation Oncology, University of Toronto, Toronto, ON Canada; 339 Division of Cancer Sciences, Manchester Cancer Research Centre, University of Manchester, Manchester, UK; 340 Radiation Medicine Program, Princess Margaret Cancer Centre, Toronto, ON Canada; 341 Department of Pathology, Brigham and Women’s Hospital, Harvard Medical School, Boston, MA USA; 342 Department of Surgery, Division of Thoracic Surgery, The Johns Hopkins University School of Medicine, Baltimore, MD USA; 343 Division of Molecular Pathology, The Netherlands Cancer Institute, Oncode Institute, Amsterdam, CX The Netherlands; 344 Department of Biomolecular Engineering, University of California Santa Cruz, Santa Cruz, CA USA; 345 UC Santa Cruz Genomics Institute, University of California Santa Cruz, Santa Cruz, CA USA; 346 Division of Applied Bioinformatics, German Cancer Research Center (DKFZ), Heidelberg, Germany; 347 German Cancer Consortium (DKTK), German Cancer Research Center (DKFZ), Heidelberg, Germany; 348 National Center for Tumor Diseases (NCT) Heidelberg, Heidelberg, Germany; 349 Center for Biological Sequence Analysis, Department of Bio and Health Informatics, Technical University of Denmark, Lyngby, Denmark; 350 Novo Nordisk Foundation Center for Protein Research, University of Copenhagen, Copenhagen, Denmark; 351 Institute for Molecular Bioscience, University of Queensland, St. Lucia, Brisbane, QLD Australia; 352 Biomedical Engineering, Oregon Health and Science University, Portland, OR USA; 353 Division of Theoretical Bioinformatics, German Cancer Research Center (DKFZ), Heidelberg, Germany; 354 Institute of Pharmacy and Molecular Biotechnology and BioQuant, Heidelberg University, Heidelberg, Germany; 355 Federal Ministry of Education and Research, Berlin, Germany; 356 Melanoma Institute Australia, University of Sydney, Sydney, NSW Australia; 357 Pediatric Hematology and Oncology, University Hospital Muenster, Muenster, Germany; 358 Department of Pathology, Johns Hopkins University School of Medicine, Baltimore, MD USA; 359 McKusick-Nathans Institute of Genetic Medicine, Sidney Kimmel Comprehensive Cancer Center at Johns Hopkins University School of Medicine, Baltimore, MD USA; 360 Foundation Medicine, Inc, Cambridge, MA USA; 361 Department of Biomedical Data Science, Stanford University School of Medicine, Stanford, CA USA; 362 Department of Genetics, Stanford University School of Medicine, Stanford, CA USA; 363 Bakar Computational Health Sciences Institute and Department of Pediatrics, University of California, San Francisco, CA USA; 364 Institute of Clinical Medicine, Faculty of Medicine, University of Oslo, Oslo, Norway; 365 National Cancer Institute, National Institutes of Health, Bethesda, MD USA; 366 Royal Marsden NHS Foundation Trust, London and Sutton, UK; 367 Genome Biology Unit, European Molecular Biology Laboratory (EMBL), Heidelberg, Germany; 368 Department of Oncology, University of Cambridge, Cambridge, UK; 369 Li Ka Shing Centre, Cancer Research UK Cambridge Institute, University of Cambridge, Cambridge, UK; 370 Institut Gustave Roussy, Villejuif, France; 371 Cambridge University Hospitals NHS Foundation Trust, Cambridge, UK; 372 Department of Haematology, University of Cambridge, Cambridge, UK; 373 Anatomia Patológica, Hospital Clinic, Institut d’Investigacions Biomèdiques August Pi i Sunyer (IDIBAPS), University of Barcelona, Barcelona, Spain; 374 Spanish Ministry of Science and Innovation, Madrid, Spain; 375 University of Michigan Comprehensive Cancer Center, Ann Arbor, MI USA; 376 Department for BioMedical Research, University of Bern, Bern, Switzerland; 377 Department of Medical Oncology, Inselspital, University Hospital and University of Bern, Bern, Switzerland; 378 Graduate School for Cellular and Biomedical Sciences, University of Bern, Bern, Switzerland; 379 University of Pavia, Pavia, Italy; 380 University of Alabama at Birmingham, Birmingham, AL USA; 381 UHN Program in BioSpecimen Sciences, Toronto General Hospital, Toronto, ON Canada; 382 Department of Urology, Icahn School of Medicine at Mount Sinai, New York, NY USA; 383 Centre for Law and Genetics, University of Tasmania, Sandy Bay Campus, Hobart, TAS Australia; 384 Faculty of Biosciences, Heidelberg University, Heidelberg, Germany; 385 Department of Biochemistry, Microbiology and Immunology, Faculty of Medicine, University of Ottawa, Ottawa, ON Canada; 386 Division of Anatomic Pathology, Mayo Clinic, Rochester, MN USA; 387 Division of Cancer Epidemiology and Genetics, National Cancer Institute, National Institutes of Health, Bethesda, MD USA; 388 Illawarra Shoalhaven Local Health District L3 Illawarra Cancer Care Centre, Wollongong Hospital, Wollongong, NSW Australia; 389 BioForA, French National Institute for Agriculture, Food, and Environment (INRAE), ONF, Orléans, France; 390 Department of Biostatistics, Bloomberg School of Public Health, Johns Hopkins University, Baltimore, MD USA; 391 University of California San Diego, San Diego, CA USA; 392 Division of Experimental Pathology, Mayo Clinic, Rochester, MN USA; 393 Centre for Cancer Research, The Westmead Institute for Medical Research, University of Sydney, Sydney, NSW Australia; 394 Department of Gynaecological Oncology, Westmead Hospital, Sydney, NSW Australia; 395 PDXen Biosystems Inc, Seoul, South Korea; 396 Korea Advanced Institute of Science and Technology, Daejeon, South Korea; 397 Electronics and Telecommunications Research Institute, Daejeon, South Korea; 398 Institut National du Cancer (INCA), Boulogne-Billancourt, France; 399 Department of Genetics, Informatics Institute, University of Alabama at Birmingham, Birmingham, AL USA; 400 Division of Medical Oncology, National Cancer Centre, Singapore, Singapore; 401 Medical Oncology, University and Hospital Trust of Verona, Verona, Italy; 402 Department of Pediatrics, University Hospital Schleswig-Holstein, Kiel, Germany; 403 Hepatobiliary/Pancreatic Surgical Oncology Program, University Health Network, Toronto, ON Canada; 404 School of Biological Sciences, University of Auckland, Auckland, New Zealand; 405 Department of Surgery, University of Melbourne, Parkville, VIC Australia; 406 The Murdoch Children’s Research Institute, Royal Children’s Hospital, Parkville, VIC Australia; 407 Walter and Eliza Hall Institute, Parkville, VIC Australia; 408 Vancouver Prostate Centre, Vancouver, Canada; 409 Lunenfeld-Tanenbaum Research Institute, Mount Sinai Hospital, Toronto, ON Canada; 410 University of East Anglia, Norwich, UK; 411 Norfolk and Norwich University Hospital NHS Trust, Norwich, UK; 412 Victorian Institute of Forensic Medicine, Southbank, VIC Australia; 413 Department of Biomedical Informatics, Harvard Medical School, Boston, MA USA; 414 Department of Chemistry, Centre for Molecular Science Informatics, University of Cambridge, Cambridge, UK; 415 Ludwig Center at Harvard Medical School, Boston, MA USA; 416 Human Genome Sequencing Center, Baylor College of Medicine, Houston, TX USA; 417 Peter MacCallum Cancer Centre, University of Melbourne, Melbourne, VIC Australia; 418 Physics Division, Optimization and Systems Biology Lab, Massachusetts General Hospital, Boston, MA USA; 419 Department of Medicine, Baylor College of Medicine, Houston, TX USA; 420 University of Cologne, Cologne, Germany; 421 International Genomics Consortium, Phoenix, AZ USA; 422 Genomics Research Program, Ontario Institute for Cancer Research, Toronto, ON Canada; 423 Barking Havering and Redbridge University Hospitals NHS Trust, Romford, UK; 424 Children’s Hospital at Westmead, University of Sydney, Sydney, NSW Australia; 425 Department of Medicine, Section of Endocrinology, University and Hospital Trust of Verona, Verona, Italy; 426 Computational Biology Center, Memorial Sloan Kettering Cancer Center, New York, NY USA; 427 Department of Biology, ETH Zurich, Zürich, Switzerland; 428 Department of Computer Science, ETH Zurich, Zurich, Switzerland; 429 SIB Swiss Institute of Bioinformatics, Lausanne, Switzerland; 430 Weill Cornell Medical College, New York, NY USA; 431 Academic Department of Medical Genetics, University of Cambridge, Addenbrooke’s Hospital, Cambridge, UK; 432 MRC Cancer Unit, University of Cambridge, Cambridge, UK; 433 Departments of Pediatrics and Genetics, University of North Carolina at Chapel Hill, Chapel Hill, NC USA; 434 Seven Bridges Genomics, Charlestown, MA USA; 435 Annai Systems, Inc, Carlsbad, CA USA; 436 Department of Pathology, General Hospital of Treviso, Department of Medicine, University of Padua, Treviso, Italy; 437 Department of Computational Biology, University of Lausanne, Lausanne, Switzerland; 438 Department of Genetic Medicine and Development, University of Geneva Medical School, Geneva, CH Switzerland; 439 Swiss Institute of Bioinformatics, University of Geneva, Geneva, CH Switzerland; 440 The Francis Crick Institute, London, UK; 441 University of Leuven, Leuven, Belgium; 442 Institute of Medical Genetics and Applied Genomics, University of Tübingen, Tübingen, Germany; 443 Computational and Systems Biology, Genome Institute of Singapore, Singapore, Singapore; 444 School of Computing, National University of Singapore, Singapore, Singapore; 445 Big Data Institute, Li Ka Shing Centre, University of Oxford, Oxford, UK; 446 Biomedical Data Science Laboratory, Francis Crick Institute, London, UK; 447 Bioinformatics Group, Department of Computer Science, University College London, London, UK; 448 The Edward S. Rogers Sr. Department of Electrical and Computer Engineering, University of Toronto, Toronto, ON Canada; 449 Breast Cancer Translational Research Laboratory JC Heuson, Institut Jules Bordet, Brussels, Belgium; 450 Department of Oncology, Laboratory for Translational Breast Cancer Research, KU Leuven, Leuven, Belgium; 451 Institute for Research in Biomedicine (IRB Barcelona), The Barcelona Institute of Science and Technology, Barcelona, Spain; 452 Research Program on Biomedical Informatics, Universitat Pompeu Fabra, Barcelona, Spain; 453 Division of Medical Oncology, Princess Margaret Cancer Centre, Toronto, ON Canada; 454 Department of Physiology and Biophysics, Weill Cornell Medicine, New York, NY USA; 455 Institute for Computational Biomedicine, Weill Cornell Medicine, New York, NY USA; 456 Department of Pathology, UPMC Shadyside, Pittsburgh, PA USA; 457 Independent Consultant, Wellesley, USA; 458 Department of Cell and Molecular Biology, Science for Life Laboratory, Uppsala University, Uppsala, Sweden; 459 Department of Medicine and Department of Genetics, Washington University School of Medicine, St. Louis, St. Louis, MO USA; 460 Hefei University of Technology, Anhui, China; 461 Translational Cancer Research Unit, GZA Hospitals St.-Augustinus, Center for Oncological Research, Faculty of Medicine and Health Sciences, University of Antwerp, Antwerp, Belgium; 462 Simon Fraser University, Burnaby, BC Canada; 463 University of Pennsylvania, Philadelphia, PA USA; 464 Faculty of Science and Technology, University of Vic—Central University of Catalonia (UVic-UCC), Vic, Spain; 465 The Wellcome Trust, London, UK; 466 The Hospital for Sick Children, Toronto, ON Canada; 467 Department of Pathology, Queen Elizabeth University Hospital, Glasgow, UK; 468 Department of Genetics and Computational Biology, QIMR Berghofer Medical Research Institute, Brisbane, QLD Australia; 469 Department of Oncology, Centre for Cancer Genetic Epidemiology, University of Cambridge, Cambridge, UK; 470 Department of Public Health and Primary Care, Centre for Cancer Genetic Epidemiology, University of Cambridge, Cambridge, UK; 471 Prostate Cancer Canada, Toronto, ON Canada; 472 University of Cambridge, Cambridge, UK; 473 Department of Laboratory Medicine, Translational Cancer Research, Lund University Cancer Center at Medicon Village, Lund University, Lund, Sweden; 474 Heidelberg University, Heidelberg, Germany; 475 New BIH Digital Health Center, Berlin Institute of Health (BIH) and Charité - Universitätsmedizin Berlin, Berlin, Germany; 476 CIBER Epidemiología y Salud Pública (CIBERESP), Madrid, Spain; 477 Research Group on Statistics, Econometrics and Health (GRECS), UdG, Barcelona, Spain; 478 Quantitative Genomics Laboratories (qGenomics), Barcelona, Spain; 479 Icelandic Cancer Registry, Icelandic Cancer Society, Reykjavik, Iceland; 480 State Key Laboratory of Cancer Biology, and Xijing Hospital of Digestive Diseases, Fourth Military Medical University, Shaanxi, China; 481 Department of Medicine (DIMED), Surgical Pathology Unit, University of Padua, Padua, Italy; 482 Rigshospitalet, Copenhagen, Denmark; 483 Center for Cancer Genomics, National Cancer Institute, National Institutes of Health, Bethesda, MD USA; 484 Department of Biochemistry and Molecular Medicine, University of Montreal, Montreal, QC Canada; 485 Australian Institute of Tropical Health and Medicine, James Cook University, Douglas, QLD Australia; 486 Department of Neuro-Oncology, Istituto Neurologico Besta, Milano, Italy; 487 Bioplatforms Australia, North Ryde, NSW Australia; 488 Department of Pathology (Research), University College London Cancer Institute, London, UK; 489 Department of Surgical Oncology, Princess Margaret Cancer Centre, Toronto, ON Canada; 490 Department of Medical Oncology, Josephine Nefkens Institute and Cancer Genomics Centre, Erasmus Medical Center, Rotterdam, CN The Netherlands; 491 The University of Queensland Thoracic Research Centre, The Prince Charles Hospital, Brisbane, QLD Australia; 492 CIBIO/InBIO - Research Center in Biodiversity and Genetic Resources, Universidade do Porto, Vairão, Portugal; 493 HCA Laboratories, London, UK; 494 University of Liverpool, Liverpool, UK; 495 The Azrieli Faculty of Medicine, Bar-Ilan University, Safed, Israel; 496 Department of Neurosurgery, University of Florida, Gainesville, FL USA; 497 Department of Pathology, Graduate School of Medicine, University of Tokyo, Tokyo, Japan; 498 University of Milano Bicocca, Monza, Italy; 499 BGI-Shenzhen, Shenzhen, China; 500 Department of Pathology, Oslo University Hospital Ulleval, Oslo, Norway; 501 Center for Biomedical Informatics, Harvard Medical School, Boston, MA USA; 502 Department Biochemistry and Molecular Biomedicine, University of Barcelona, Barcelona, Spain; 503 Office of Cancer Genomics, National Cancer Institute, National Institutes of Health, Bethesda, MD USA; 504 Cancer Epigenomics, German Cancer Research Center (DKFZ), Heidelberg, Germany; 505 Department of Cancer Biology, The University of Texas MD Anderson Cancer Center, Houston, TX USA; 506 Department of Surgical Oncology, The University of Texas MD Anderson Cancer Center, Houston, TX USA; 507 Department of Computer Science, Yale University, New Haven, CT USA; 508 Department of Molecular Biophysics and Biochemistry, Yale University, New Haven, CT USA; 509 Program in Computational Biology and Bioinformatics, Yale University, New Haven, CT USA; 510 Center for Cancer Research, Massachusetts General Hospital, Boston, MA USA; 511 Department of Pathology, Massachusetts General Hospital, Boston, MA USA; 512 Department of Pathology, Memorial Sloan Kettering Cancer Center, New York, NY USA; 513 Division of Gastroenterology and Hepatology, Mayo Clinic, Rochester, MN USA; 514 University of Sydney, Sydney, NSW Australia; 515 University of Oxford, Oxford, UK; 516 Department of Surgery, Academic Urology Group, University of Cambridge, Cambridge, UK; 517 Department of Medicine II, University of Würzburg, Wuerzburg, Germany; 518 Sylvester Comprehensive Cancer Center, University of Miami, Miami, FL USA; 519 Institut Hospital del Mar d’Investigacions Mèdiques (IMIM), Barcelona, Spain; 520 Genome Integrity and Structural Biology Laboratory, National Institute of Environmental Health Sciences (NIEHS), Durham, NC USA; 521 St. Thomas’s Hospital, London, UK; 522 Osaka International Cancer Center, Osaka, Japan; 523 Department of Pathology, Skåne University Hospital, Lund University, Lund, Sweden; 524 Department of Medical Oncology, Beatson West of Scotland Cancer Centre, Glasgow, UK; 525 National Human Genome Research Institute, National Institutes of Health, Bethesda, MD USA; 526 Centre for Cancer Research, Victorian Comprehensive Cancer Centre, University of Melbourne, Melbourne, VIC Australia; 527 Department of Medicine, Section of Hematology/Oncology, University of Chicago, Chicago, IL USA; 528 German Center for Infection Research (DZIF), Partner Site Hamburg-Borstel-Lübeck-Riems, Hamburg, Germany; 529 Bioinformatics Research Centre (BiRC), Aarhus University, Aarhus, Denmark; 530 Department of Biotechnology, Ministry of Science and Technology, Government of India, New Delhi, Delhi India; 531 National Cancer Centre Singapore, Singapore, Singapore; 532 Brandeis University, Waltham, MA USA; 533 Department of Urologic Sciences, University of British Columbia, Vancouver, BC Canada; 534 Department of Internal Medicine, Stanford University, Stanford, CA USA; 535 The University of Texas Health Science Center at Houston, Houston, TX USA; 536 Imperial College NHS Trust, Imperial College, London, INY UK; 537 Senckenberg Institute of Pathology, University of Frankfurt Medical School, Frankfurt, Germany; 538 Department of Medicine, Division of Biomedical Informatics, UC San Diego School of Medicine, San Diego, CA USA; 539 Center for Precision Health, School of Biomedical Informatics, The University of Texas Health Science Center, Houston, TX USA; 540 Oxford Nanopore Technologies, New York, NY USA; 541 Institute of Medical Science, University of Tokyo, Tokyo, Japan; 542 Howard Hughes Medical Institute, University of California Santa Cruz, Santa Cruz, CA USA; 543 Wakayama Medical University, Wakayama, Japan; 544 Department of Internal Medicine, Division of Medical Oncology, Lineberger Comprehensive Cancer Center, University of North Carolina at Chapel Hill, Chapel Hill, NC USA; 545 University of Tennessee Health Science Center for Cancer Research, Memphis, TN USA; 546 Department of Histopathology, Salford Royal NHS Foundation Trust, Salford, UK; 547 Faculty of Biology, Medicine and Health, University of Manchester, Manchester, UK; 548 BIOPIC, ICG and College of Life Sciences, Peking University, Beijing, China; 549 Peking-Tsinghua Center for Life Sciences, Peking University, Beijing, China; 550 Children’s Hospital of Philadelphia, Philadelphia, PA USA; 551 Department of Bioinformatics and Computational Biology and Department of Systems Biology, The University of Texas MD Anderson Cancer Center, Houston, TX USA; 552 Karolinska Institute, Stockholm, Sweden; 553 The Donnelly Centre, University of Toronto, Toronto, ON Canada; 554 Department of Medical Genetics, College of Medicine, Hallym University, Chuncheon, South Korea; 555 Department of Experimental and Health Sciences, Institute of Evolutionary Biology (UPF-CSIC), Universitat Pompeu Fabra, Barcelona, Spain; 556 Health Data Science Unit, University Clinics, Heidelberg, Germany; 557 Massachusetts General Hospital Center for Cancer Research, Charlestown, MA USA; 558 Hokkaido University, Sapporo, Japan; 559 Department of Pathology and Clinical Laboratory, National Cancer Center Hospital, Tokyo, Japan; 560 Department of Genetics, University of North Carolina at Chapel Hill, Chapel Hill, NC USA; 561 Computational Biology, Leibniz Institute on Aging - Fritz Lipmann Institute (FLI), Jena, Germany; 562 University of Melbourne Centre for Cancer Research, Melbourne, VIC Australia; 563 University of Nebraska Medical Center, Omaha, NE USA; 564 Syntekabio Inc, Daejeon, South Korea; 565 Department of Pathology, Academic Medical Center, Amsterdam, AZ The Netherlands; 566 China National GeneBank-Shenzhen, Shenzhen, China; 567 Division of Molecular Genetics, German Cancer Research Center (DKFZ), Heidelberg, Germany; 568 Division of Life Science and Applied Genomics Center, Hong Kong University of Science and Technology, Clear Water Bay, Hong Kong, China; 569 Icahn School of Medicine at Mount Sinai, New York, NY USA; 570 Geneplus-Shenzhen, Shenzhen, China; 571 School of Computer Science and Technology, Xi’an Jiaotong University, Xi’an, China; 572 AbbVie, North Chicago, IL USA; 573 Institute of Pathology, Charité – University Medicine Berlin, Berlin, Germany; 574 Centre for Translational and Applied Genomics, British Columbia Cancer Agency, Vancouver, BC Canada; 575 Edinburgh Royal Infirmary, Edinburgh, UK; 576 Berlin Institute for Medical Systems Biology, Max Delbrück Center for Molecular Medicine, Berlin, Germany; 577 Department of Pediatric Immunology, Hematology and Oncology, University Hospital, Heidelberg, Germany; 578 German Cancer Research Center (DKFZ), Heidelberg, Germany; 579 Heidelberg Institute for Stem Cell Technology and Experimental Medicine (HI-STEM), Heidelberg, Germany; 580 Institute for Computational Biomedicine, Weill Cornell Medical College, New York, NY USA; 581 New York Genome Center, New York, NY USA; 582 Department of Urology, James Buchanan Brady Urological Institute, Johns Hopkins University School of Medicine, Baltimore, MD USA; 583 Department of Preventive Medicine, Graduate School of Medicine, The University of Tokyo, Tokyo, Japan; 584 Department of Molecular and Cellular Biology, Baylor College of Medicine, Houston, TX USA; 585 Department of Pathology and Immunology, Baylor College of Medicine, Houston, TX USA; 586 Michael E. DeBakey Veterans Affairs Medical Center, Houston, TX USA; 587 Technical University of Denmark, Lyngby, Denmark; 588 Department of Pathology, College of Medicine, Hanyang University, Seoul, South Korea; 589 Academic Unit of Surgery, School of Medicine, College of Medical, Veterinary and Life Sciences, University of Glasgow, Glasgow Royal Infirmary, Glasgow, UK; 590 Department of Pathology, Asan Medical Center, College of Medicine, Ulsan University, Songpa-gu, Seoul South Korea; 591 Science Writer, Garrett Park, MD USA; 592 International Cancer Genome Consortium (ICGC)/ICGC Accelerating Research in Genomic Oncology (ARGO) Secretariat, Ontario Institute for Cancer Research, Toronto, ON Canada; 593 University of Ljubljana, Ljubljana, Slovenia; 594 Department of Public Health Sciences, University of Chicago, Chicago, IL USA; 595 Research Institute, NorthShore University HealthSystem, Evanston, IL USA; 596 Department for Biomedical Research, University of Bern, Bern, Switzerland; 597 Centre of Genomics and Policy, McGill University and Génome Québec Innovation Centre, Montreal, QC Canada; 598 Carolina Center for Genome Sciences, University of North Carolina at Chapel Hill, Chapel Hill, NC USA; 599 Hopp Children’s Cancer Center (KiTZ), Heidelberg, Germany; 600 Pediatric Glioma Research Group, German Cancer Research Center (DKFZ), Heidelberg, Germany; 601 Cancer Research UK, London, UK; 602 Indivumed GmbH, Hamburg, Germany; 603 Genome Integration Data Center, Syntekabio, Inc, Daejeon, South Korea; 604 University Hospital Zurich, Zurich, Switzerland; 605 Clinical Bioinformatics, Swiss Institute of Bioinformatics, Geneva, Switzerland; 606 Institute for Pathology and Molecular Pathology, University Hospital Zurich, Zurich, Switzerland; 607 Institute of Molecular Life Sciences, University of Zurich, Zurich, Switzerland; 608 MRC Human Genetics Unit, MRC IGMM, University of Edinburgh, Edinburgh, UK; 609 Women’s Cancer Program at the Samuel Oschin Comprehensive Cancer Institute, Cedars-Sinai Medical Center, Los Angeles, CA USA; 610 Department of Biology, Bioinformatics Group, Division of Molecular Biology, Faculty of Science, University of Zagreb, Zagreb, Croatia; 611 Department for Internal Medicine II, University Hospital Schleswig-Holstein, Kiel, Germany; 612 Genetics and Molecular Pathology, SA Pathology, Adelaide, SA Australia; 613 Department of Gastric Surgery, National Cancer Center Hospital, Tokyo, Japan; 614 Department of Bioinformatics, Division of Cancer Genomics, National Cancer Center Research Institute, Tokyo, Japan; 615 A.A. Kharkevich Institute of Information Transmission Problems, Moscow, Russia; 616 Oncology and Immunology, Dmitry Rogachev National Research Center of Pediatric Hematology, Moscow, Russia; 617 Skolkovo Institute of Science and Technology, Moscow, Russia; 618 Department of Surgery, The George Washington University, School of Medicine and Health Science, Washington, DC USA; 619 Endocrine Oncology Branch, Center for Cancer Research, National Cancer Institute, National Institutes of Health, Bethesda, MD USA; 620 Melanoma Institute Australia, Macquarie University, Sydney, NSW Australia; 621 MIT Computer Science and Artificial Intelligence Laboratory, Massachusetts Institute of Technology, Cambridge, MA USA; 622 Tissue Pathology and Diagnostic Oncology, Royal Prince Alfred Hospital, Sydney, NSW Australia; 623 Cholangiocarcinoma Screening and Care Program and Liver Fluke and Cholangiocarcinoma Research Centre, Faculty of Medicine, Khon Kaen University, Khon Kaen, Thailand; 624 Controlled Department and Institution, New York, NY USA; 625 Englander Institute for Precision Medicine, Weill Cornell Medicine, New York, NY USA; 626 National Cancer Center, Gyeonggi, South Korea; 627 Department of Biochemistry, College of Medicine, Ewha Womans University, Seoul, South Korea; 628 Health Sciences Department of Biomedical Informatics, University of California San Diego, La Jolla, CA USA; 629 Research Core Center, National Cancer Centre Korea, Goyang-si, South Korea; 630 Department of Health Sciences and Technology, Sungkyunkwan University School of Medicine, Seoul, South Korea; 631 Samsung Genome Institute, Seoul, South Korea; 632 Breast Oncology Program, Dana-Farber/Brigham and Women’s Cancer Center, Boston, MA USA; 633 Department of Surgery, Memorial Sloan Kettering Cancer Center, New York, NY USA; 634 Division of Breast Surgery, Brigham and Women’s Hospital, Boston, MA USA; 635 Integrative Bioinformatics Support Group, National Institute of Environmental Health Sciences (NIEHS), Durham, NC USA; 636 Department of Clinical Science, University of Bergen, Bergen, Norway; 637 Center For Medical Innovation, Seoul National University Hospital, Seoul, South Korea; 638 Department of Internal Medicine, Seoul National University Hospital, Seoul, South Korea; 639 Institute of Computer Science, Polish Academy of Sciences, Warsawa, Poland; 640 Functional and Structural Genomics, German Cancer Research Center (DKFZ), Heidelberg, Germany; 641 Laboratory of Translational Genomics, Division of Cancer Epidemiology and Genetics, National Cancer Institute, , National Institutes of Health, Bethesda, MD USA; 642 Institute for Medical Informatics Statistics and Epidemiology, University of Leipzig, Leipzig, Germany; 643 Morgan Welch Inflammatory Breast Cancer Research Program and Clinic, The University of Texas MD Anderson Cancer Center, Houston, TX USA; 644 Department of Hematology and Oncology, Georg-Augusts-University of Göttingen, Göttingen, Germany; 645 Institute of Cell Biology (Cancer Research), University of Duisburg-Essen, Essen, Germany; 646 King’s College London and Guy’s and St. Thomas’ NHS Foundation Trust, London, UK; 647 Center for Epigenetics, Van Andel Research Institute, Grand Rapids, MI USA; 648 The University of Queensland Centre for Clinical Research, Royal Brisbane and Women’s Hospital, Herston, QLD Australia; 649 Department of Pediatric Oncology and Hematology, University of Cologne, Cologne, Germany; 650 University of Düsseldorf, Düsseldorf, Germany; 651 Department of Pathology, Institut Jules Bordet, Brussels, Belgium; 652 Institute of Biomedicine, Sahlgrenska Academy at University of Gothenburg, Gothenburg, Sweden; 653 Children’s Medical Research Institute, Sydney, NSW Australia; 654 ILSbio, LLC Biobank, Chestertown, MD USA; 655 Division of Genetics and Genomics, Boston Children’s Hospital, Harvard Medical School, Boston, MA USA; 656 Institute for Bioengineering and Biopharmaceutical Research (IBBR), Hanyang University, Seoul, South Korea; 657 Department of Statistics, University of California Santa Cruz, Santa Cruz, CA USA; 658 National Genotyping Center, Institute of Biomedical Sciences, Academia Sinica, Taipei, Taiwan; 659 Department of Vertebrate Genomics/Otto Warburg Laboratory Gene Regulation and Systems Biology of Cancer, Max Planck Institute for Molecular Genetics, Berlin, Germany; 660 McGill University and Genome Quebec Innovation Centre, Montreal, QC Canada; 661 biobyte solutions GmbH, Heidelberg, Germany; 662 Gynecologic Oncology, NYU Laura and Isaac Perlmutter Cancer Center, New York University, New York, NY USA; 663 Division of Oncology, Stem Cell Biology Section, Washington University School of Medicine, St. Louis, MO USA; 664 Department of Systems Biology, The University of Texas MD Anderson Cancer Center, Houston, TX USA; 665 Harvard University, Cambridge, MA USA; 666 Urologic Oncology Branch, Center for Cancer Research, National Cancer Institute, National Institutes of Health, Bethesda, MD USA; 667 University of Oslo, Oslo, Norway; 668 University of Toronto, Toronto, ON Canada; 669 Peking University, Beijing, China; 670 School of Life Sciences, Peking University, Beijing, China; 671 Leidos Biomedical Research, Inc, McLean, VA USA; 672 Hematology, Hospital Clinic, Institut d’Investigacions Biomèdiques August Pi i Sunyer (IDIBAPS), University of Barcelona, Barcelona, Spain; 673 Second Military Medical University, Shanghai, China; 674 Chinese Cancer Genome Consortium, Shenzhen, China; 675 Department of Medical Oncology, Beijing Hospital, Beijing, China; 676 Laboratory of Molecular Oncology, Key Laboratory of Carcinogenesis and Translational Research (Ministry of Education), Peking University Cancer Hospital and Institute, Beijing, China; 677 School of Medicine/School of Mathematics and Statistics, University of St. Andrews, St, Andrews, Fife UK; 678 Institute for Systems Biology, Seattle, WA USA; 679 Department of Biochemistry and Molecular Biology, Faculty of Medicine, University Institute of Oncology-IUOPA, Oviedo, Spain; 680 Institut Bergonié, Bordeaux, France; 681 Cancer Unit, MRC University of Cambridge, Cambridge, UK; 682 Department of Pathology and Laboratory Medicine, Center for Personalized Medicine, Children’s Hospital Los Angeles, Los Angeles, CA USA; 683 John Curtin School of Medical Research, Canberra, ACT Australia; 684 MVZ Department of Oncology, PraxisClinic am Johannisplatz, Leipzig, Germany; 685 Department of Information Technology, Ghent University, Ghent, Belgium; 686 Department of Plant Biotechnology and Bioinformatics, Ghent University, Ghent, Belgium; 687 Institute for Genomic Medicine, Nationwide Children’s Hospital, Columbus, OH USA; 688 Computational Biology Program, School of Medicine, Oregon Health and Science University, Portland, OR USA; 689 Department of Surgery, Duke University, Durham, NC USA; 690 Institució Catalana de Recerca i Estudis Avançats (ICREA), Barcelona, Spain; 691 Institut Català de Paleontologia Miquel Crusafont, Universitat Autònoma de Barcelona, Barcelona, Spain; 692 University of Glasgow, Glasgow, UK; 693 Institut d’Investigacions Biomèdiques August Pi i Sunyer (IDIBAPS), Barcelona, Spain; 694 Division of Oncology, Washington University School of Medicine, St. Louis, MO USA; 695 Department of Surgery and Cancer, Imperial College, London, INY UK; 696 Applications Department, Oxford Nanopore Technologies, Oxford, UK; 697 Department of Obstetrics, Gynecology and Reproductive Services, University of California San Francisco, San Francisco, CA USA; 698 Department of Biochemistry and Molecular Medicine, University California at Davis, Sacramento, CA USA; 699 STTARR Innovation Facility, Princess Margaret Cancer Centre, Toronto, ON Canada; 700 Discipline of Surgery, Western Sydney University, Penrith, NSW Australia; 701 Yale School of Medicine, Yale University, New Haven, CT USA; 702 Department of Genetics, Lineberger Comprehensive Cancer Center, University of North Carolina at Chapel Hill, Chapel Hill, NC USA; 703 Departments of Neurology and Neurosurgery, Henry Ford Hospital, Detroit, MI USA; 704 Precision Oncology, OHSU Knight Cancer Institute, Oregon Health and Science University, Portland, OR USA; 705 Institute of Pathology, University Medical Center Hamburg-Eppendorf, Hamburg, Germany; 706 Department of Health Sciences, Faculty of Medical Sciences, Kyushu University, Fukuoka, Japan; 707 Heidelberg Academy of Sciences and Humanities, Heidelberg, Germany; 708 Department of Clinical Pathology, University of Melbourne, Melbourne, VIC, Australia; 709 Department of Pathology, Roswell Park Cancer Institute, Buffalo, NY USA; 710 Department of Computer Science, University of Helsinki, Helsinki, Finland; 711 Institute of Biotechnology, University of Helsinki, Helsinki, Finland; 712 Organismal and Evolutionary Biology Research Programme, University of Helsinki, Helsinki, Finland; 713 Department of Obstetrics and Gynecology, Division of Gynecologic Oncology, Washington University School of Medicine, St. Louis, MO USA; 714 Penrose St. Francis Health Services, Colorado Springs, CO USA; 715 Institute of Pathology, Ulm University and University Hospital of Ulm, Ulm, Germany; 716 National Cancer Center, Tokyo, Japan; 717 Genome Institute of Singapore, Singapore, Singapore; 718 32Program in Computational Biology and Bioinformatics, Yale University, New Haven, CT USA; 719 German Cancer Aid, Bonn, Germany; 720 Programme in Cancer and Stem Cell Biology, Centre for Computational Biology, Duke-NUS Medical School, Singapore, Singapore; 721 The Chinese University of Hong Kong, Shatin, NT, Hong Kong China; 722 Fourth Military Medical University, Shaanxi, China; 723 The University of Cambridge School of Clinical Medicine, Cambridge, UK; 724 St. Jude Children’s Research Hospital, Memphis, TN USA; 725 University Health Network, Princess Margaret Cancer Centre, Toronto, ON Canada; 726 Center for Biomolecular Science and Engineering, University of California Santa Cruz, Santa Cruz, CA USA; 727 Department of Medicine, University of Chicago, Chicago, IL USA; 728 Department of Neurology, Mayo Clinic, Rochester, MN USA; 729 Cambridge Oesophagogastric Centre, Cambridge University Hospitals NHS Foundation Trust, Cambridge, UK; 730 Department of Computer Science, Carleton College, Northfield, MN USA; 731 Institute of Cancer Sciences, College of Medical Veterinary and Life Sciences, University of Glasgow, Glasgow, UK; 732 Department of Epidemiology, University of Alabama at Birmingham, Birmingham, AL USA; 733 HudsonAlpha Institute for Biotechnology, Huntsville, AL USA; 734 O’Neal Comprehensive Cancer Center, University of Alabama at Birmingham, Birmingham, AL USA; 735 Department of Pathology, Keio University School of Medicine, Tokyo, Japan; 736 Department of Hepatobiliary and Pancreatic Oncology, National Cancer Center Hospital, Tokyo, Japan; 737 Sage Bionetworks, Seattle, WA USA; 738 Lymphoma Genomic Translational Research Laboratory, National Cancer Centre, Singapore, Singapore; 739 Department of Clinical Pathology, Robert-Bosch-Hospital, Stuttgart, Germany; 740 Department of Cell and Systems Biology, University of Toronto, Toronto, ON Canada; 741 Department of Biosciences and Nutrition, Karolinska Institutet, Stockholm, Sweden; 742 Center for Liver Cancer, Research Institute and Hospital, National Cancer Center, Gyeonggi, South Korea; 743 Division of Hematology-Oncology, Samsung Medical Center, Sungkyunkwan University School of Medicine, Seoul, South Korea; 744 Samsung Advanced Institute for Health Sciences and Technology, Sungkyunkwan University School of Medicine, Seoul, South Korea; 745 Cheonan Industry-Academic Collaboration Foundation, Sangmyung University, Cheonan, South Korea; 746 NYU Langone Medical Center, New York, NY USA; 747 Department of Hematology and Medical Oncology, Cleveland Clinic, Cleveland, OH USA; 748 Department of Radiation Oncology, University of California San Francisco, San Francisco, CA USA; 749 Department of Health Sciences Research, Mayo Clinic, Rochester, MN USA; 750 Helen F. Graham Cancer Center at Christiana Care Health Systems, Newark, DE USA; 751 Heidelberg University Hospital, Heidelberg, Germany; 752 CSRA Incorporated, Fairfax, VA USA; 753 Research Department of Pathology, University College London Cancer Institute, London, UK; 754 Department of Research Oncology, Guy’s Hospital, King’s Health Partners AHSC, King’s College London School of Medicine, London, UK; 755 Faculty of Medicine and Health Sciences, Macquarie University, Sydney, NSW Australia; 756 University Hospital of Minjoz, INSERM UMR 1098, Besançon, France; 757 Spanish National Cancer Research Centre, Madrid, Spain; 758 Center of Digestive Diseases and Liver Transplantation, Fundeni Clinical Institute, Bucharest, Romania; 759 Cureline, Inc, South San Francisco, CA USA; 760 St. Luke’s Cancer Centre, Royal Surrey County Hospital NHS Foundation Trust, Guildford, UK; 761 Cambridge Breast Unit, Addenbrooke’s Hospital, Cambridge University Hospital NHS Foundation Trust and NIHR Cambridge Biomedical Research Centre, Cambridge, UK; 762 East of Scotland Breast Service, Ninewells Hospital, Aberdeen, UK; 763 Department of Genetics, Microbiology and Statistics, University of Barcelona, IRSJD, IBUB, Barcelona, Spain; 764 Department of Obstetrics and Gynecology, Medical College of Wisconsin, Milwaukee, WI USA; 765 Hematology and Medical Oncology, Winship Cancer Institute of Emory University, Atlanta, GA USA; 766 Department of Computer Science, Princeton University, Princeton, NJ USA; 767 Vanderbilt Ingram Cancer Center, Vanderbilt University, Nashville, TN USA; 768 Ohio State University College of Medicine and Arthur G. James Comprehensive Cancer Center, Columbus, OH USA; 769 Department of Surgery, Yokohama City University Graduate School of Medicine, Kanagawa, Japan; 770 Division of Chromatin Networks, German Cancer Research Center (DKFZ) and BioQuant, Heidelberg, Germany; 771 Research Computing Center, University of North Carolina at Chapel Hill, Chapel Hill, NC USA; 772 School of Molecular Biosciences and Center for Reproductive Biology, Washington State University, Pullman, WA USA; 773 Finsen Laboratory and Biotech Research and Innovation Centre (BRIC), University of Copenhagen, Copenhagen, Denmark; 774 Department of Laboratory Medicine and Pathobiology, University of Toronto, Toronto, ON Canada; 775 Department of Pathology, Human Oncology and Pathogenesis Program, Memorial Sloan Kettering Cancer Center, New York, NY USA; 776 University Hospital Giessen, Pediatric Hematology and Oncology, Giessen, Germany; 777 Oncologie Sénologie, ICM Institut Régional du Cancer, Montpellier, France; 778 Institute of Clinical Molecular Biology, Christian-Albrechts-University, Kiel, Germany; 779 Institute of Pathology, University of Wuerzburg, Wuerzburg, Germany; 780 Department of Urology, North Bristol NHS Trust, Bristol, UK; 781 SingHealth, Duke-NUS Institute of Precision Medicine, National Heart Centre Singapore, Singapore, Singapore; 782 Department of Computer Science, University of Toronto, Toronto, ON Canada; 783 Bern Center for Precision Medicine, University Hospital of Bern, University of Bern, Bern, Switzerland; 784 Englander Institute for Precision Medicine, Weill Cornell Medicine and New York Presbyterian Hospital, New York, NY USA; 785 Meyer Cancer Center, Weill Cornell Medicine, New York, NY USA; 786 Pathology and Laboratory, Weill Cornell Medical College, New York, NY USA; 787 Vall d’Hebron Institute of Oncology: VHIO, Barcelona, Spain; 788 General and Hepatobiliary-Biliary Surgery, Pancreas Institute, University and Hospital Trust of Verona, Verona, Italy; 789 National Centre for Biological Sciences, Tata Institute of Fundamental Research, Bangalore, India; 790 Indiana University, Bloomington, IN USA; 791 Department of Pathology, GZA-ZNA Hospitals, Antwerp, Belgium; 792 Analytical Biological Services, Inc, Wilmington, DE USA; 793 Sydney Medical School, University of Sydney, Sydney, NSW Australia; 794 cBio Center, Dana-Farber Cancer Institute, Harvard Medical School, Boston, MA USA; 795 Department of Cell Biology, Harvard Medical School, Boston, MA USA; 796 Advanced Centre for Treatment Research and Education in Cancer, Tata Memorial Centre, Navi Mumbai, Maharashtra India; 797 School of Environmental and Life Sciences, Faculty of Science, The University of Newcastle, Ourimbah, NSW Australia; 798 Department of Dermatology, University Hospital of Essen, Essen, Germany; 799 Bioinformatics and Omics Data Analytics, German Cancer Research Center (DKFZ), Heidelberg, Germany; 800 Department of Urology, Charité Universitätsmedizin Berlin, Berlin, Germany; 801 Martini-Clinic, Prostate Cancer Center, University Medical Center Hamburg-Eppendorf, Hamburg, Germany; 802 Department of General Internal Medicine, University of Kiel, Kiel, Germany; 803 German Cancer Consortium (DKTK), Partner site Berlin, Berlin, Germany; 804 Cancer Research Institute, Beth Israel Deaconess Medical Center, Boston, MA USA; 805 University of Pittsburgh, Pittsburgh, PA USA; 806 Department of Ophthalmology and Ocular Genomics Institute, Massachusetts Eye and Ear, Harvard Medical School, Boston, MA USA; 807 Center for Psychiatric Genetics, NorthShore University HealthSystem, Evanston, IL USA; 808 Van Andel Research Institute, Grand Rapids, MI USA; 809 Laboratory of Molecular Medicine, Human Genome Center, Institute of Medical Science, University of Tokyo, Tokyo, Japan; 810 Japan Agency for Medical Research and Development, Tokyo, Japan; 811 Korea University, Seoul, South Korea; 812 Murtha Cancer Center, Walter Reed National Military Medical Center, Bethesda, MD USA; 813 Human Genetics, University of Kiel, Kiel, Germany; 814 Department of Oncologic Pathology, Dana-Farber Cancer Institute, Harvard Medical School, Boston, MA USA; 815 Oregon Health and Science University, Portland, OR USA; 816 Center for RNA Interference and Noncoding RNA, The University of Texas MD Anderson Cancer Center, Houston, TX USA; 817 Department of Experimental Therapeutics, The University of Texas MD Anderson Cancer Center, Houston, TX USA; 818 Department of Gynecologic Oncology and Reproductive Medicine, The University of Texas MD Anderson Cancer Center, Houston, TX USA; 819 University Hospitals Coventry and Warwickshire NHS Trust, Coventry, UK; 820 Department of Radiation Oncology, Radboud University Nijmegen Medical Centre, Nijmegen, GA The Netherlands; 821 Institute for Genomics and Systems Biology, University of Chicago, Chicago, IL USA; 822 Clinic for Hematology and Oncology, St.-Antonius-Hospital, Eschweiler, Germany; 823 Computational and Systems Biology Program, Memorial Sloan Kettering Cancer Center, New York, NY USA; 824 University of Iceland, Reykjavik, Iceland; 825 Division of Computational Genomics and Systems Genetics, German Cancer Research Center (DKFZ), Heidelberg, Germany; 826 Dundee Cancer Centre, Ninewells Hospital, Dundee, UK; 827 Department for Internal Medicine III, University of Ulm and University Hospital of Ulm, Ulm, Germany; 828 Institut Curie, INSERM Unit 830, Paris, France; 829 Department of Gastroenterology and Hepatology, Yokohama City University Graduate School of Medicine, Kanagawa, Japan; 830 Department of Laboratory Medicine, Radboud University Nijmegen Medical Centre, Nijmegen, GA The Netherlands; 831 Division of Cancer Genome Research, German Cancer Research Center (DKFZ), Heidelberg, Germany; 832 Department of General Surgery, Singapore General Hospital, Singapore, Singapore; 833 Cancer Science Institute of Singapore, National University of Singapore, Singapore, Singapore; 834 Department of Medical and Clinical Genetics, Genome-Scale Biology Research Program, University of Helsinki, Helsinki, Finland; 835 East Anglian Medical Genetics Service, Cambridge University Hospitals NHS Foundation Trust, Cambridge, UK; 836 Irving Institute for Cancer Dynamics, Columbia University, New York, NY USA; 837 Institute of Molecular and Cell Biology, Singapore, Singapore; 838 Laboratory of Cancer Epigenome, Division of Medical Science, National Cancer Centre Singapore, Singapore, Singapore; 839 Universite Lyon, INCa-Synergie, Centre Léon Bérard, Lyon, France; 840 Department of Urology, Mayo Clinic, Rochester, MN USA; 841 Royal National Orthopaedic Hospital - Stanmore, Stanmore, Middlesex UK; 842 Department of Biochemistry, Genetics and Immunology, University of Vigo, Vigo, Spain; 843 Giovanni Paolo II / I.R.C.C.S. Cancer Institute, Bari, BA Italy; 844 Neuroblastoma Genomics, German Cancer Research Center (DKFZ), Heidelberg, Germany; 845 Fondazione Policlinico Universitario Gemelli IRCCS, Rome, Italy, Rome, Italy; 846 University of Verona, Verona, Italy; 847 Centre National de Génotypage, CEA - Institute de Génomique, Evry, France; 848 CAPHRI Research School, Maastricht University, Maastricht, ER The Netherlands; 849 Department of Biopathology, Centre Léon Bérard, Lyon, France; 850 Université Claude Bernard Lyon 1, Villeurbanne, France; 851 Core Research for Evolutional Science and Technology (CREST), JST, Tokyo, Japan; 852 Department of Biological Sciences, Laboratory for Medical Science Mathematics, Graduate School of Science, University of Tokyo, Yokohama, Japan; 853 Department of Medical Science Mathematics, Medical Research Institute, Tokyo Medical and Dental University (TMDU), Tokyo, Japan; 854 Cancer Ageing and Somatic Mutation Programme, Wellcome Sanger Institute, Hinxton, UK; 855 University Hospitals Birmingham NHS Foundation Trust, Birmingham, UK; 856 Centre for Cancer Research and Cell Biology, Queen’s University, Belfast, UK; 857 Breast Medical Oncology, The University of Texas MD Anderson Cancer Center, Houston, TX USA; 858 Department of Surgery, Johns Hopkins University School of Medicine, Baltimore, MD USA; 859 Department of Oncology-Pathology, Science for Life Laboratory, Karolinska Institute, Stockholm, Sweden; 860 School of Cancer Sciences, Faculty of Medicine, University of Southampton, Southampton, UK; 861 Department of Gene Technology, Tallinn University of Technology, Tallinn, Estonia; 862 Genetics and Genome Biology Program, SickKids Research Institute, The Hospital for Sick Children, Toronto, ON Canada; 863 Departments of Neurosurgery and Hematology and Medical Oncology, Winship Cancer Institute and School of Medicine, Emory University, Atlanta, GA USA; 864 Department of Clinical and Molecular Medicine, Faculty of Medicine and Health Sciences, Norwegian University of Science and Technology, Trondheim, Norway; 865 Argmix Consulting, North Vancouver, BC Canada; 866 Department of Information Technology, Ghent University, Interuniversitair Micro-Electronica Centrum (IMEC), Ghent, Belgium; 867 Nuffield Department of Surgical Sciences, John Radcliffe Hospital, University of Oxford, Oxford, UK; 868 Institute of Mathematics and Computer Science, University of Latvia, Riga, LV Latvia; 869 Discipline of Pathology, Sydney Medical School, University of Sydney, Sydney, NSW Australia; 870 Department of Applied Mathematics and Theoretical Physics, Centre for Mathematical Sciences, University of Cambridge, Cambridge, UK; 871 Department of Epidemiology and Biostatistics, Memorial Sloan Kettering Cancer Center, New York, NY USA; 872 Department of Statistics, Columbia University, New York, NY USA; 873 Department of Immunology, Genetics and Pathology, Science for Life Laboratory, Uppsala University, Uppsala, Sweden; 874 School of Electronic and Information Engineering, Xi’an Jiaotong University, Xi’an, China; 875 Department of Histopathology, Cambridge University Hospitals NHS Foundation Trust, Cambridge, UK; 876 Oxford NIHR Biomedical Research Centre, University of Oxford, Oxford, UK; 877 Georgia Regents University Cancer Center, Augusta, GA USA; 878 Wythenshawe Hospital, Manchester, UK; 879 Department of Genetics, Washington University School of Medicine, St.Louis, MO USA; 880 Department of Biological Oceanography, Leibniz Institute of Baltic Sea Research, Rostock, Germany; 881 Wellcome Centre for Human Genetics, University of Oxford, Oxford, UK; 882 Department of Molecular and Human Genetics, Baylor College of Medicine, Houston, TX USA; 883 Thoracic Oncology Laboratory, Mayo Clinic, Rochester, MN USA; 884 Institute for Genomic Medicine, Nationwide Children’s Hospital, Columbus, OH USA; 885 Department of Obstetrics and Gynecology, Division of Gynecologic Oncology, Mayo Clinic, Rochester, MN USA; 886 International Institute for Molecular Oncology, Poznań, Poland; 887 Poznan University of Medical Sciences, Poznań, Poland; 888 Genomics and Proteomics Core Facility High Throughput Sequencing Unit, German Cancer Research Center (DKFZ), Heidelberg, Germany; 889 NCCS-VARI Translational Research Laboratory, National Cancer Centre Singapore, Singapore, Singapore; 890 Edison Family Center for Genome Sciences and Systems Biology, Washington University, St. Louis, MO USA; 891 MRC-University of Glasgow Centre for Virus Research, Glasgow, UK; 892 Department of Medical Informatics and Clinical Epidemiology, Division of Bioinformatics and Computational Biology, OHSU Knight Cancer Institute, Oregon Health and Science University, Portland, OR USA; 893 School of Electronic Information and Communications, Huazhong University of Science and Technology, Wuhan, China; 894 Department of Applied Mathematics and Statistics, Johns Hopkins University, Baltimore, MD USA; 895 Department of Cancer Genome Informatics, Graduate School of Medicine, Osaka University, Osaka, Japan; 896 Institute of Computer Science, Heidelberg University, Heidelberg, Germany; 897 School of Mathematics and Statistics, University of Sydney, Sydney, NSW Australia; 898 Ben May Department for Cancer Research, University of Chicago, Chicago, IL USA; 899 Department of Human Genetics, University of Chicago, Chicago, IL USA; 900 Tri-Institutional PhD Program in Computational Biology and Medicine, Weill Cornell Medicine, New York, NY USA; 901 The First Affiliated Hospital, Xi’an Jiaotong University, Xi’an, China; 902 Department of Medicine and Therapeutics, The Chinese University of Hong Kong, Shatin, NT, Hong Kong China; 903 Department of Biostatistics, The University of Texas MD Anderson Cancer Center, Houston, TX USA; 904 Duke-NUS Medical School, Singapore, Singapore; 905 Department of Surgery, Ruijin Hospital, Shanghai Jiaotong University School of Medicine, Shanghai, China; 906 School of Computing Science, University of Glasgow, Glasgow, UK; 907 Division of Orthopaedic Surgery, Oslo University Hospital, Oslo, Norway; 908 Eastern Clinical School, Monash University, Melbourne, VIC Australia; 909 Epworth HealthCare, Richmond, VIC Australia; 910 Department of Biostatistics and Computational Biology, Dana-Farber Cancer Institute and Harvard Medical School, Boston, MA USA; 911 Department of Biomedical Informatics, College of Medicine, The Ohio State University, Columbus, OH USA; 912 The Ohio State University Comprehensive Cancer Center (OSUCCC – James), Columbus, OH USA; 913 The University of Texas School of Biomedical Informatics (SBMI) at Houston, Houston, TX USA; 914 Department of Biostatistics, University of North Carolina at Chapel Hill, Chapel Hill, NC USA; 915 Department of Biochemistry and Molecular Genetics, Feinberg School of Medicine, Northwestern University, Chicago, IL USA; 916 Faculty of Medicine and Health, University of Sydney, Sydney, NSW Australia; 917 Department of Pathology, Erasmus Medical Center Rotterdam, Rotterdam, GD The Netherlands; 918 Division of Molecular Carcinogenesis, The Netherlands Cancer Institute, Amsterdam, CX The Netherlands; 919 Institute of Molecular Life Sciences and Swiss Institute of Bioinformatics, University of Zurich, Zurich, Switzerland

**Keywords:** Cancer genetics, Mutation

## Abstract

Somatic mutations in cancer genomes are caused by multiple mutational processes, each of which generates a characteristic mutational signature^1^. Here, as part of the Pan-Cancer Analysis of Whole Genomes (PCAWG) Consortium^2^ of the International Cancer Genome Consortium (ICGC) and The Cancer Genome Atlas (TCGA), we characterized mutational signatures using 84,729,690 somatic mutations from 4,645 whole-genome and 19,184 exome sequences that encompass most types of cancer. We identified 49 single-base-substitution, 11 doublet-base-substitution, 4 clustered-base-substitution and 17 small insertion-and-deletion signatures. The substantial size of our dataset, compared with previous analyses^3–15^, enabled the discovery of new signatures, the separation of overlapping signatures and the decomposition of signatures into components that may represent associated—but distinct—DNA damage, repair and/or replication mechanisms. By estimating the contribution of each signature to the mutational catalogues of individual cancer genomes, we revealed associations of signatures to exogenous or endogenous exposures, as well as to defective DNA-maintenance processes. However, many signatures are of unknown cause. This analysis provides a systematic perspective on the repertoire of mutational processes that contribute to the development of human cancer.

## Abstract (web summary)

The characterization of 4,645 whole-genome and 19,184 exome sequences, covering most types of cancer, identifies 81 single-base substitution, doublet-base substitution and small-insertion-and-deletion mutational signatures, providing a systematic overview of the mutational processes that contribute to cancer development.

## Main

Somatic mutations in cancer genomes are caused by mutational processes of both exogenous and endogenous origin that operate during the cell lineage between the fertilized egg and the cancer cell^16^. Each mutational process may involve components of DNA damage or modification, DNA repair and DNA replication (which may be normal or abnormal), and generates a characteristic mutational signature that potentially includes base substitutions, small insertions and deletions (indels), genome rearrangements and chromosome copy-number changes^1^. The mutations in an individual cancer genome may have been generated by multiple mutational processes, and thus incorporate multiple superimposed mutational signatures. Therefore, to systematically characterize the mutational processes that contribute to cancer, mathematical methods have previously been used to decipher mutational signatures from somatic mutation catalogues, estimate the number of mutations that are attributable to each signature in individual samples and annotate each mutation class in each tumour with the probability that it arose from each signature^6,9,17–27^.

Previous studies of multiple types of cancer have identified more than 30 single-base substitution (SBS) signatures, some of known—but many of unknown—aetiologies, some ubiquitous and others rare, some part of normal cell biology and others associated with abnormal exposures or neoplastic progression^3–5,7–15^. Genome rearrangement signatures have also previously been described^11,25,28–30^. However, the analysis of other classes of mutation has been relatively limited^3,11,31–33^.

Mutational signature analysis has predominantly used cancer exome sequences. However, the many-fold-greater numbers of somatic mutations in whole genomes provide substantially increased power for signature decomposition, enabling the better separation of partially correlated signatures and the extraction of signatures that contribute relatively small numbers of mutations. Furthermore, technical artefacts and differences in sequencing technologies and mutation-calling algorithms can themselves generate mutational signatures. Therefore, the uniformly processed and highly curated sets of all classes of somatic mutations from the 2,780 cancer genomes of the PCAWG project^2^, combined with most other suitable cancer genomes (accession code syn11801889, available at https://www.synapse.org/#!Synapse:syn11801889), present a notable opportunity to establish the repertoire of mutational signatures and determine their activities across different types of cancer. The timing of these signatures during the evolution of individual cancers and the repertoire of signatures of structural variation have been explored in other PCAWG analyses^30,34^.

## Mutational signature analysis

The 23,829 samples—which include most types of cancer, and comprise the 2,780 PCAWG whole genomes^2^, 1,865 additional whole genomes and 19,184 exomes—yielded 79,793,266 somatic SBSs, 814,191 doublet-base substitutions (DBSs) and 4,122,233 small indels that were analysed for mutational signatures, about 10-fold-more mutations than any previous study of which we are aware (syn11801889)^6^.

We developed classifications for each type of mutation. For SBSs, the primary classification comprised 96 classes (available at https://cancer.sanger.ac.uk/cosmic/signatures/SBS) constituted by the 6 base substitutions C>A, C>G, C>T, T>A, T>C and T>G (in which the mutated base is represented by the pyrimidine of the base pair), plus the flanking 5′ and 3′ bases. In some analyses, two flanking bases 5′ and 3′ to the mutated base were considered (producing 1,536 classes) or mutations within transcribed genome regions were selected and classified according to whether the mutated pyrimidine fell on the transcribed or untranscribed strand (producing 192 classes). We also derived a classification for DBSs (78 classes; available at https://cancer.sanger.ac.uk/cosmic/signatures/DBS). Indels were classified as deletions or insertions and—when of a single base—as C or T, and according to the length of the mononucleotide repeat tract in which they occurred. Longer indels were classified as occurring at repeats or with overlapping microhomology at deletion boundaries, and according to the size of indel, repeat and microhomology (83 classes; available at https://cancer.sanger.ac.uk/cosmic/signatures/ID).

The PCAWG whole-genome sequences, the additional whole-genome sequences and the exome sequences were each analysed separately (syn11801889)^2^. Signatures were extracted from each type of cancer individually, from all cancer types together, as separate SBS, DBS and indel signatures, and as composite signatures of all three types of mutation (Supplementary Note 2).

We used two methods based on nonnegative matrix factorization (NMF): SigProfiler, an elaborated version of the framework used for the previous ‘Catalogue Of Somatic Mutations In Cancer’ (COSMIC) compendium of mutational signatures (COSMIC v.2, available at https://cancer.sanger.ac.uk/cosmic/signatures_v2)^11,17^, and SignatureAnalyzer, which is based on a Bayesian variant of NMF^9,27,35^. NMF determines the signature profiles and contributions of each signature to each cancer genome as part of its factorization of the input matrix of mutation spectra. However, with many signatures and/or heterogeneous mutation burdens across samples, the mutations observed in a particular sample can be reconstructed in multiple ways—often with small and/or biologically implausible contributions from many signatures. Therefore, each method has developed a separate procedure for estimating the contributions of signatures to each sample (Methods).

We tested SignatureAnalyzer and SigProfiler on 11 sets of synthetic data (including 64,400 synthetic samples), generated from known signature profiles (Methods, Supplementary Note 2). Both methods performed well in re-extracting known signatures from realistically complex data. Extracted signatures that were discordant from the known input usually arose from difficulties in selecting the correct number of signatures. The results confirm that use of NMF-based approaches for extracting mutational signatures is not a purely algorithmic process, but also requires consideration of evidence from experimentally determined mutational signatures and the DNA damage and repair literature, prior evidence of biological plausibility and human-guided sensitivity analysis confirming that extractions from different groupings of tumours yield consistent results. We used these types of evidence and approaches in determining the signature profiles reported here. The findings are consistent with results regarding NMF, and the related areas of probabilistic topic modelling and latent Dirichlet allocation, in multiple problem domains^36,37^. It is widely understood that the choice of the number of latent variables (for our purposes, the number of mutational signatures) is rarely amenable to complete automation.

**Extended Data Fig. 1:**
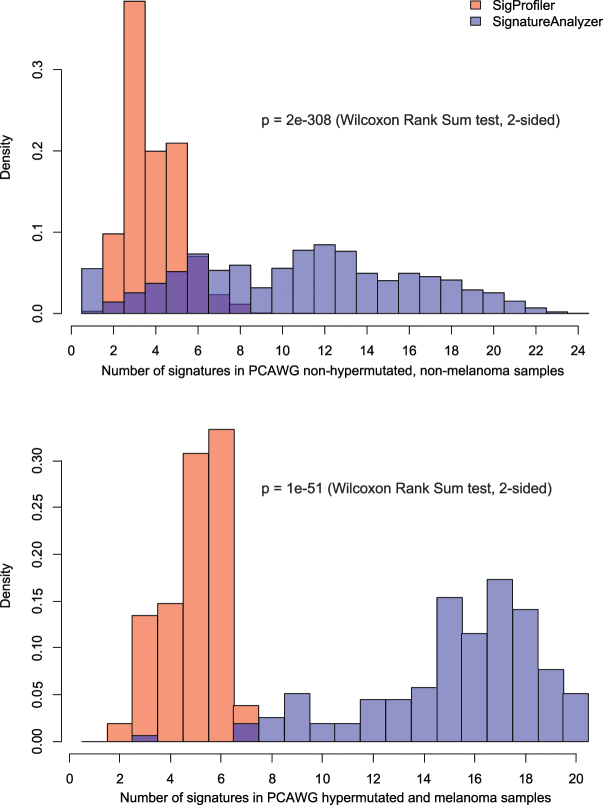
Histogram of the number of signatures attributed in each of 2,780 PCAWG samples by SigProfiler and SignatureAnalyzer. Hypermutated tumours and melanomas (156) are listed at syn11738314.

**Extended Data Fig. 2:**
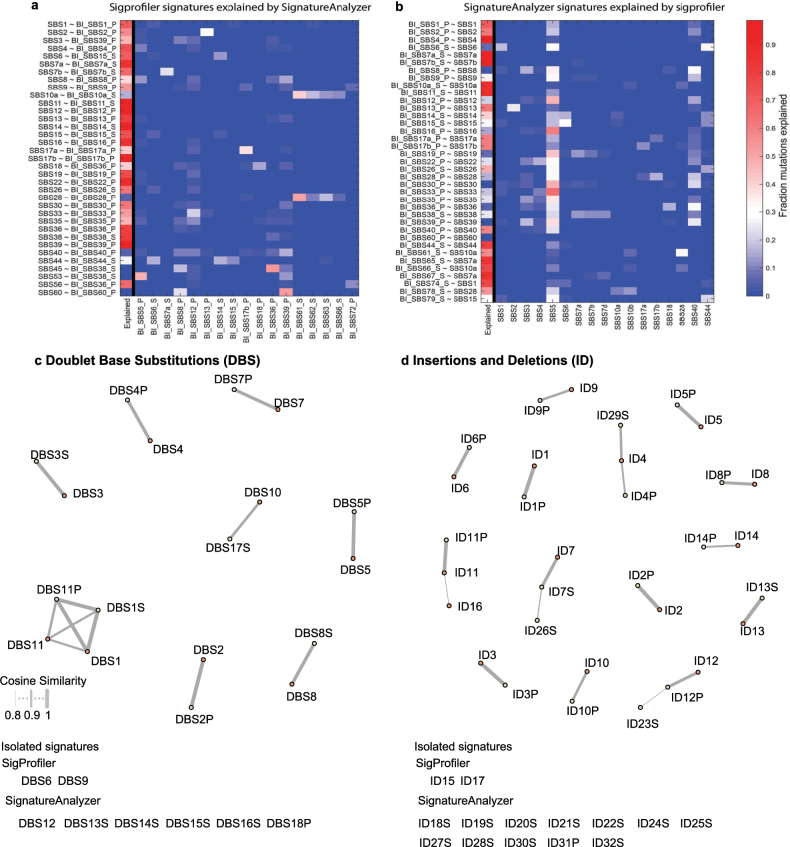
Comparisons between results of SigProfiler and SignatureAnalyzer. **a**, **b**, Comparison of the attributions for corresponding SigProfiler (**a**) and SignatureAnalyzer (**b**) signatures. Each one of the SBS signatures extracted by SigProfiler and SignatureAnalyzer was paired with the signature of highest cosine similarity in the extraction by the other method (if one with >0.85 cosine similarity exists). The first column of the plot corresponds to the fraction of mutations assigned by one method (summed across samples and mutation types) that was also assigned by the other method. The remaining mutations were then redistributed to the other signatures in the extraction, weighted by their relative probabilities of having been generated by each signature and the resulting fraction of mutations was then plotted. Signatures on the *x* axis are shown only if they contribute at least a 0.1 fraction of mutations to at least one signature on the *y* axis. **c**, **d**, Cosine similarities between SigProfiler and SignatureAnalyzer DBS (**c**) and indel (**d**) signatures. Brown nodes represent SigProfiler signatures; green nodes represent SignatureAnalyzer signatures. Matches with cosine similarities > 0.8 are shown as edges; the width of the edge indicates the strength of the similarity. The locations of the nodes have no meaning. Signatures with no matches of >0.8 cosine similarity are shown below. SigProfiler ID15 and ID17 were extracted from data that were not analysed by SignatureAnalyzer. The suffix ‘P’ on a SignatureAnalyzer signature name indicates a signature extracted from non-hypermutated, non-melanoma tumours. The suffix ‘S’ on a SignatureAnalyzer signature name indicates a signature extracted from hypermutated or melanoma tumours.

The results from our SigProfiler and SignatureAnalyzer analyses of cancer data exhibited many similarities, and we assigned the same identifiers to similar signatures extracted using the two methods (syn12016215). However, there were also noteworthy differences. The numbers of SBS signatures found in PCAWG tumours with a low mutation burden (94.4% of cases that contain 47% of mutations) were similar: 31 using SigProfiler and 35 using SignatureAnalyzer. However, the numbers of additional SBS signatures extracted from hypermutated PCAWG samples (5.6% of cases, containing 53% of mutations) were different: 13 using SigProfiler and 25 using SignatureAnalyzer. There were also differences in SBS signature profiles, including among signatures found in cases with a low mutation burden. The latter primarily involved relatively featureless (‘flat’) signatures, which are mathematically challenging to deconvolute. Finally, there were differences in signature attributions to individual samples. SignatureAnalyzer used more signatures to reconstruct the mutational profiles (Extended Data Fig. 1) (syn12169204 and syn12177011) and attributions to flat signatures were different (Extended Data Fig. 2a, b) (syn12169204). The DBS and indel signatures were generally similar between the two methods (Extended Data Fig. 2c, d).

**Extended Data Fig. 3:**
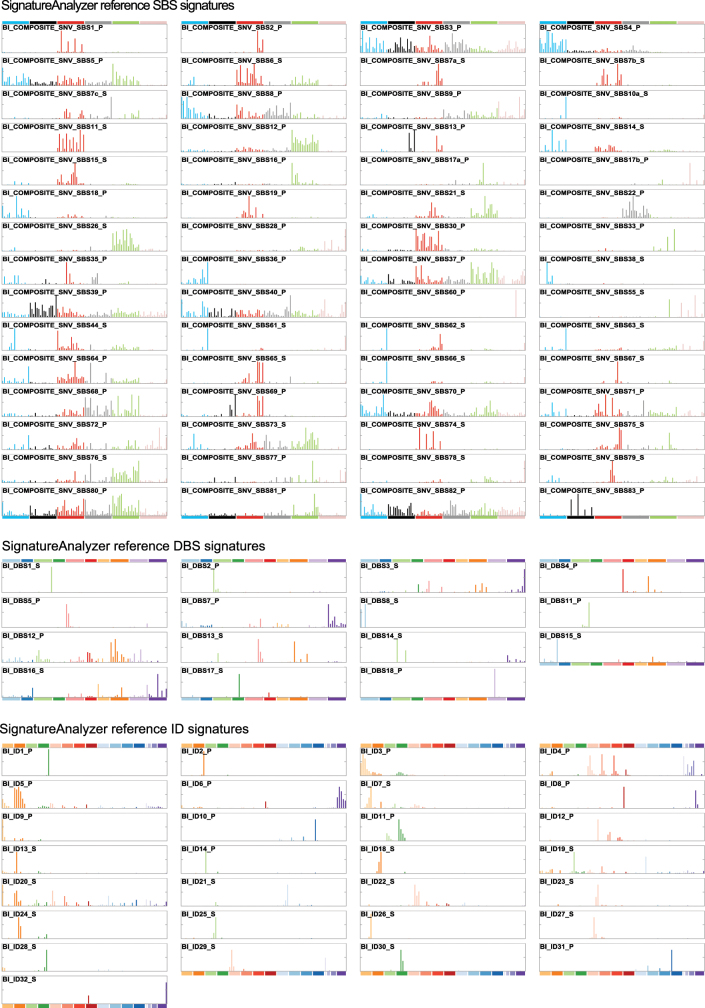
SignatureAnalyzer reference signatures. The classifications of each mutation type (SBS, 96 classes; DBS, 78 classes; and indels, 83 classes) are described in the main text.

**Extended Data Fig. 4:**
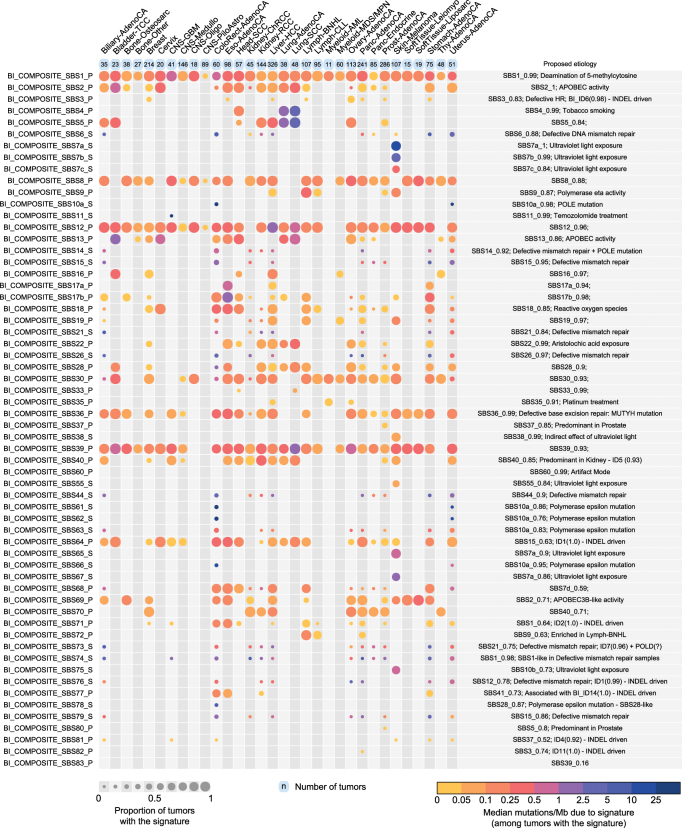
The number of SBS mutations attributed to each mutational signature for each cancer type over the PCAWG tumours by SignatureAnalyzer. Conventions are as in Fig. 3; see this figure for explanation.

The final reference mutational signatures were determined from the PCAWG set, supplemented by additional signatures from the other datasets (COSMIC, available at https://cancer.sanger.ac.uk/cosmic/signatures). Each signature was allocated an identifier consistent with, and extending, the COSMIC v.2 annotation. Some previous signatures split into multiple constituent signatures: these were numbered as in the previous annotation, but with additional letter suffixes (for example, SBS17 was split into SBS17a and SBS17b). DNA sequencing and analysis artefacts also generate mutational signatures. We indicate which signatures are possible artefacts but do not present them below (full information is available at https://cancer.sanger.ac.uk/cosmic/signatures). The results of both SignatureAnalyzer and SigProfiler were used throughout the study. However, for brevity and for continuity with the signature set previously displayed in COSMIC v.2—which has been widely used as a reference—SigProfiler results are outlined here, and SignatureAnalyzer results are provided in Extended Data Figs. 3, 4 and at syn11738307.

## Single-base substitution signatures

**Fig. 1:**
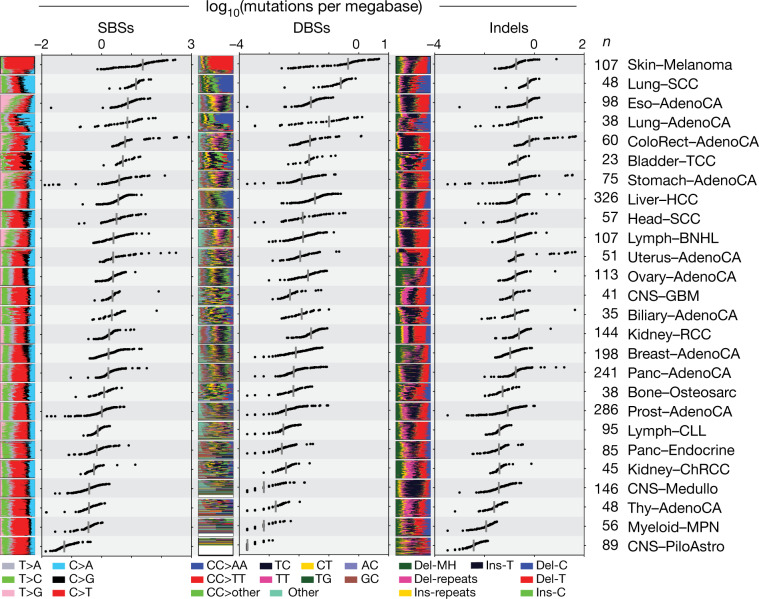
Mutation burdens of SBSs, DBSs and small indels  across PCAWG tumour types. The numbers of cases of each tumour type are shown next to the labels. Each dot represents one tumour. Tumour types are ordered by the median numbers of single-base substitutions. Only tumour types with >20 samples are shown. AdenoCA, adenocarcinoma; BNHL, B-cell non-Hodgkin lymphoma; ChRCC, chromophobe renal cell carcinoma; CLL, chronic lymphocytic leukaemia; CNS, central nervous system; ColoRect, colorectal; Eso, oesophageal; GBM, glioblastoma; HCC, hepatocellular carcinoma; Medullo, medulloblastoma; MH, microhomology; MPN, myeloproliferative neoplasm; Osteosarc, osteosarcoma; Panc, pancreatic; PiloAstro, pilocytic astrocytoma; Prost; prostate; RCC, renal cell carcinoma; SCC, squamous cell carcinoma; TCC, transitional cell carcinoma; Thy, thyroid.

**Fig. 2:**
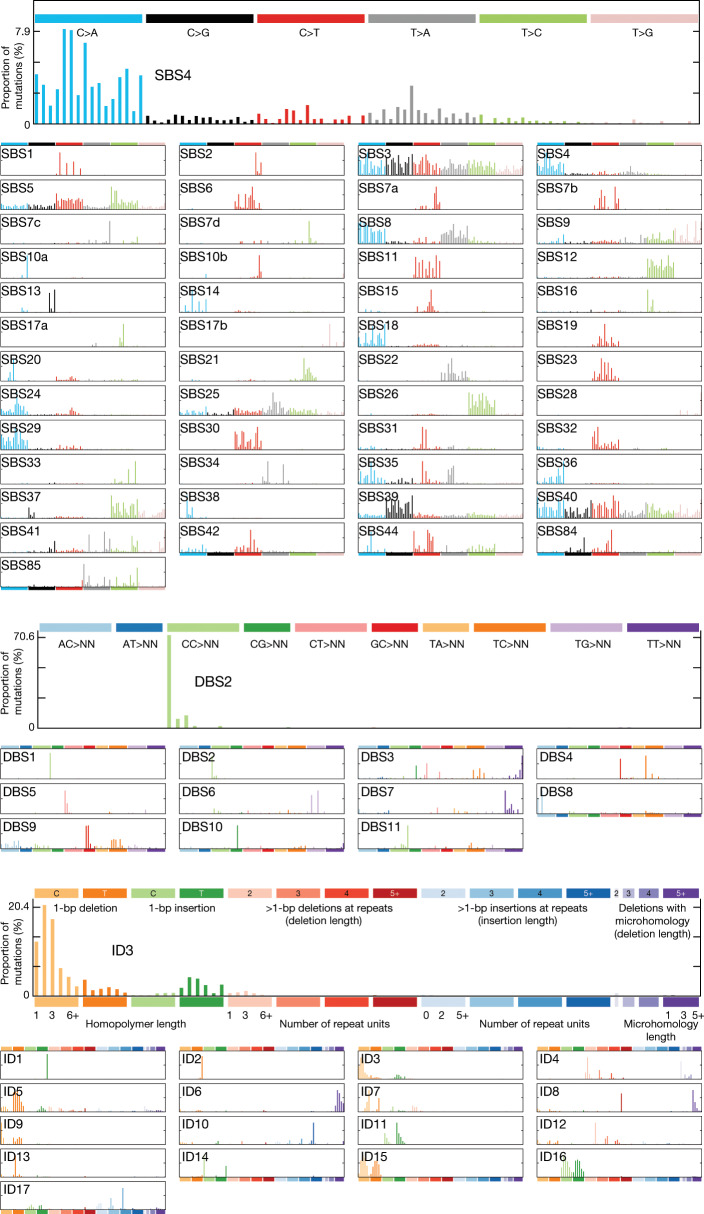
Profiles of SBS, DBS and small indel mutational signatures. The classifications of each mutation type (SBS, 96 classes; DBS, 78 classes; and indels, 83 classes) are described in the main text. Magnified versions of signatures SBS4, DBS2 and ID3 (all of which are associated with tobacco smoking) are shown to illustrate the positions of each mutation subtype on each plot. The plotted data are available in digital form (along with the *x* axis labels) at syn12025148.

There were substantial differences in the numbers of SBSs between samples (ranging from hundreds to millions) and between cancer types^38^ (Fig. 1). In total, 67 SBS mutational signatures were extracted, of which 49 were considered likely to be of biological origin (Fig. 2, Methods; available at https://cancer.sanger.ac.uk/cosmic/signatures/SBS/). Except for signature SBS25, all signatures reported in COSMIC v.2 (ref. ^6^) were confirmed; the median cosine similarity between the newly derived signatures and those on COSMIC v.2 was 0.95, excluding the ‘split’ signatures (discussed below). SBS25 was previously found in cell lines derived from Hodgkin lymphomas treated with chemotherapy, and no primary cancers of this type were available. The newly derived signatures showed much improved separation from each other and more-distinct signature profiles, as compared with COSMIC v.2 signatures (see ‘Better separation compared to COSMIC v.2 signatures’ in Supplementary Note 2 for more information).

**Fig. 3:**
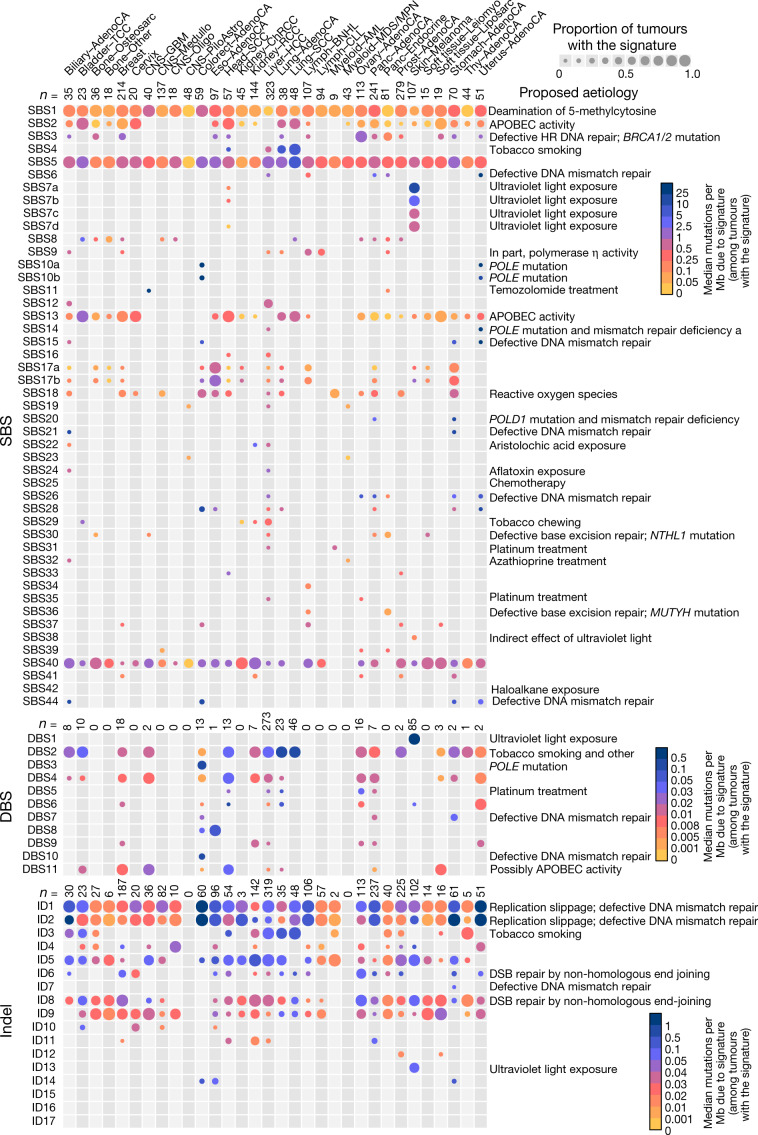
The number of mutations contributed by each mutational signature to the PCAWG tumours. The size of each dot represents the proportion of samples of each tumour type that shows the mutational signature. The colour of each dot represents the median mutation burden of the signature in samples that show the signature. Tumours that had few mutations or that were poorly reconstructed by the signature assignment were excluded. The contributions of composite signatures to the PCAWG cancers, and SBS signatures to the complete set of cancer samples analysed, are shown in Extended Data Figs. 4 and 5, respectively. AML, acute myeloid leukaemia; liposarc, liposarcoma; MDS, myelodysplastic syndrome.

**Extended Data Fig. 5:**
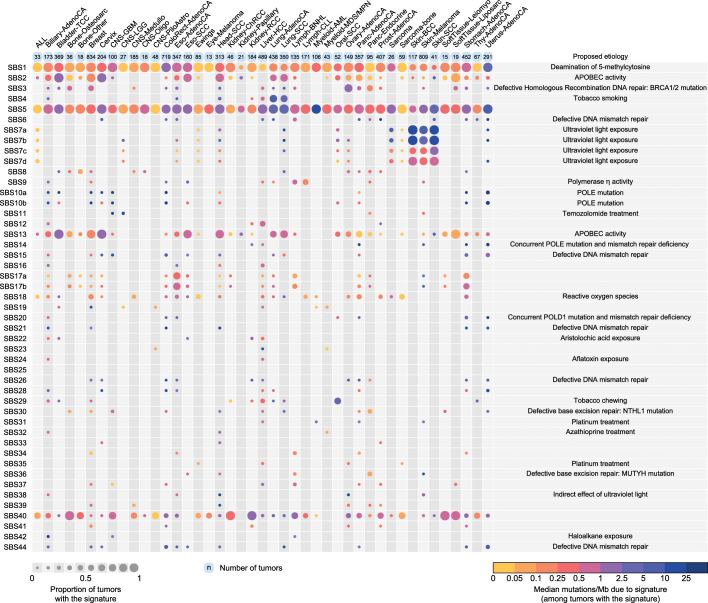
The number of SBS mutations attributed to each mutational signature to each cancer type over the complete set of PCAWG and non-PCAWG cancer samples analysed by SigProfiler. Conventions are as in Fig. 3; see this figure for explanation.

Thirteen of the SBS signatures we extracted (excluding those due to signature splitting) represent newly identified and probably real signatures, not present in COSMIC v.2. Some were rare (SBS31, SBS32, SBS35, SBS36, SBS42 and SBS44). Others were more common, but contributed relatively few mutations and/or were similar to previously discovered signatures (SBS38, SBS39 and SBS40). Notably, SBS40 is a flat signature similar to SBS5. It contributes to multiple types of cancer, but its similarity to SBS5 renders the extent of this contribution uncertain. For some of the newly identified signatures, there were plausible underlying aetiologies (Fig. 3, Extended Data Figs. 4, 5): for SBS31 and SBS35, platinum compound chemotherapy^39^; for SBS32, azathioprine therapy; for SBS36, inactivating germline or somatic mutations in *MUTYH* (which encodes a component of the base excision repair machinery)^40,41^; for SBS38, additional effects of exposure to ultraviolet (UV) light; for SBS42, occupational exposure to haloalkanes^13^; and for SBS44, defective DNA mismatch repair^42^.

Three previously characterized base substitution signatures (SBS7, SBS10 and SBS17) split into multiple constituent signatures (Fig. 2). Signature splitting probably reflects the existence of multiple distinct mutational processes initiated by the same exposure that have closely—but not perfectly—correlated activities. We previously regarded SBS7 as a single signature composed predominantly of C>T at CCN and TCN trinucleotides (the mutated base is underlined) together with many fewer T>N mutations. It was found in malignant melanomas and squamous skin carcinomas, and is probably due to the UV-light-induced formation of pyrimidine dimers, followed by translesion DNA synthesis by error-prone polymerases predominantly inserting A opposite to damaged cytosines. SBS7 has now been decomposed into four constituent signatures. SBS7a and SBS7b (consisting mainly of C>T at TCN and C>T at CCN, respectively) may reflect different pyrimidine-dimer photoproducts. SBS7c and SBS7d (consisting predominantly of T>A at NTT and T>C at NTT, respectively^43^) may be due to low frequencies of the misincorporation of T and G opposite to thymines in pyrimidine dimers. The splitting of SBS10 and SBS17 is described at https://cancer.sanger.ac.uk/cosmic/signatures/SBS/.

Several base substitution signatures showed transcriptional strand bias, which may be attributable to transcription-coupled nucleotide excision repair acting on DNA damage and/or to an excess of DNA damage on untranscribed strands of genes^44^. Both mechanisms result in more mutations of damaged bases on untranscribed than on transcribed strands of genes. Assuming that either mechanism is responsible for the observed transcriptional strand biases, DNA damage to cytosine (SBS7a and SBS7b), guanine (SBS4, SBS8, SBS19, SBS23, SBS24, SBS31, SBS32, SBS35 and SBS42), thymine (SBS7c, SBS7d, SBS21, SBS26 and SBS33) and adenine (SBS5, SBS12, SBS16, SBS22 and SBS25) may underlie these mutational signatures (plots of strand bias are available at https://cancer.sanger.ac.uk/cosmic/signatures/SBS/). The likely DNA-damaging agents are known for SBS4 (tobacco mutagens), SBS7a, SBS7b, SBS7c and SBS7d (UV light), SBS22 (aristolochic acid), SBS24 (aflatoxin), SBS25 (chemotherapy), SBS31 and SBS35 (platinum compounds), SBS32 (azathioprine) and SBS42 (haloalkanes).

Using the SBS classification of 1,536 mutation types, which uses the sequence context two bases 5′ and two bases 3′ to each mutated base, yielded signatures that are largely consistent with those based on substitutions in trinucleotide contexts. Notably, however, two forms of both SBS2 and SBS13 were extracted, one with mainly a pyrimidine and the other with mainly a purine at the −2 base (the second base 5′ to the mutated cytosine). These may represent the activities of the cytidine deaminases APOBEC3A and APOBEC3B, respectively^45^. If so, APOBEC3A accounts for many more mutations than APOBEC3B in cancers with high APOBEC activity. Other signatures showed nonrandom sequence contexts at +2 and −2 positions (for example, SBS17a, SBS17b and SBS9), but sequence context effects were generally much stronger for bases immediately 5′ and 3′ to mutated bases.

**Fig. 4:**
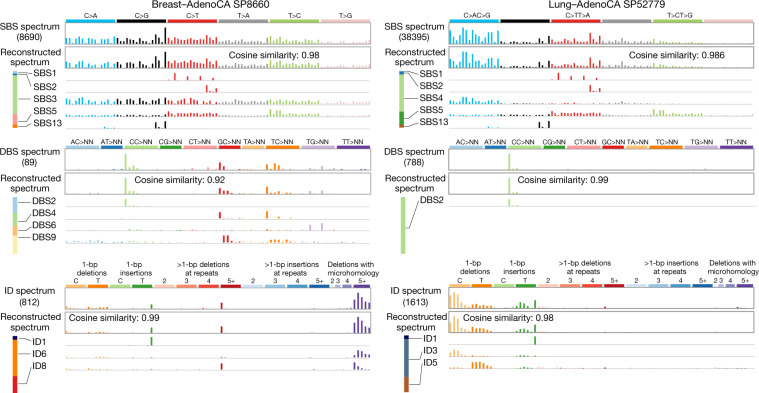
Illustrative examples of mutational spectra of individual cancer samples. The contributory SBS, DBS and small indel mutational signatures in two tumours are shown.

SBS signatures showed substantial variation in the numbers of cancer types and cancer samples in which they were found, and in the mutations attributed per cancer sample (Fig. 3). Almost all individual cancer samples exhibited multiple signatures, with a mode of three in the PCAWG set (syn12169204). The assigned signatures reconstruct well the mutational spectra of the cancer samples (in PCAWG samples, the median cosine similarity was 0.97; 96.3% of samples with cosine similarity >0.90): Fig. 4 shows illustrative examples.

Some mutational processes generate base substitutions that cluster in small genomic regions. The limited numbers of such mutations may result in a failure to detect their signatures using standard methods. We therefore identified clustered mutations in each genome and analysed them separately (Methods). Four main clustered mutational signatures were identified (Fig. 2), as previously reported^4,27,32^. Two, which are found in multiple types of cancer, were similar to SBS2 and SBS13 (which have been attributed to APOBEC enzyme activity) and represent foci of kataegis^3,32,46^. Two further clustered signatures, one characterized by C>T and C>G mutations at (A or G)C(C or T) trinucleotides^47^ and the other T>A and T>C mutations at (A or T)T(A or T), were found in lymphoid neoplasms; they probably represent the direct and indirect consequences of activation-induced cytidine deaminase mutagenesis and translesion DNA synthesis by error-prone polymerases (SBS84 and SBS85, respectively)^27^.

## Doublet-base substitution signatures

Tandem doublet, triplet, quadruplet, quintuplet and sextuplet base substitutions (syn11801938 and syn11726620) were observed at about 1% the prevalence of SBSs. In most cancer genomes, the number of DBSs was considerably higher than would be expected from the random adjacency of SBSs (syn12177057), indicating the existence of commonly occurring, single mutagenic events that cause substitutions at neighbouring bases. There was substantial variation in the number of DBSs, ranging from 0 to 20,818 in a sample. The numbers of DBSs were generally proportional to the numbers of SBSs (Fig. 1), although colorectal adenocarcinomas had fewer than expected, and lung cancers and melanomas had more (Extended Data Table 1). We extracted eleven DBS signatures (Fig. 2, of which three have previously been reported^33,48^.

Signature DBS1 was characterized by CC>TT mutations (Fig. 2), contributed hundreds to tens of thousands of mutations in malignant melanomas with SBS7a and SBS7b (Fig. 3), exhibited transcriptional strand bias consistent with damage to cytosines (syn12177063) and is a known consequence of DNA damage induced by UV light^33,49^. Excluding cancers associated with exposure to UV light also yielded a signature (DBS11) that was characterized predominantly by CC>TT mutations, but only contributing tens of mutations in many samples from multiple types of cancer (Figs. 2, 3). DBS11 was associated with SBS2, which is due to APOBEC activity: APOBEC activity may, therefore, also generate DBS11.

DBS2 was composed predominantly of CC>AA mutations, with smaller numbers of CC>AG and CC>AT mutations, and contributed hundreds to thousands of mutations in lung adenocarcinoma, lung squamous and head and neck squamous carcinomas, which are often caused by tobacco smoking^33^ (Figs. 2, 3). DBS2 showed transcriptional strand bias indicative of guanine damage (syn12177064) and was associated with SBS4, which is caused by exposure to tobacco smoke. It is likely, therefore, that DBS2 can be a consequence of DNA damage by tobacco-smoke mutagens.

A signature similar to DBS2 contributed hundreds of mutations to liver cancers and tens of mutations to other types of cancer without evidence of exposure to tobacco smoke. A pattern resembling DBS2 also dominates DBSs in healthy mouse cells^50^. The nature of the mutational processes that underlie these signatures in human cancers that are unrelated to smoking, and in healthy mice, is unknown. However, in experimental systems, acetaldehyde exposure has been shown to generate a mutational signature characterized primarily by CC>AA mutations, and lower burdens of CC>AG and CC>AT mutations, together with C>A SBSs^48^. Acetaldehyde is an oxidation product of alcohol and a constituent of cigarette smoke. The role of acetaldehyde, and perhaps other aldehydes, in generating DBS2 merits further investigation^51^.

DBS3, DBS7, DBS8 and DBS10 showed hundreds to thousands of mutations in rare colorectal, stomach and oesophageal cancers, some of which showed evidence of defective DNA mismatch repair (DBS7 and DBS10) or polymerase epsilon exonuclease domain mutations (DBS3) that generate hypermutator phenotypes (Figs. 2, 3). DBS5 was found in cancers exposed to platinum chemotherapy, and is associated with SBS31 and SBS35.

## Small insertion-and-deletion signatures

Indels were usually present at about 10% of the frequency of base substitutions (Fig. 1). There was substantial variation between cancer genomes in the number of indels, even when cancers with evidence of defective DNA mismatch repair were excluded. Overall, the numbers of deletions and insertions were similar, but there was variation between cancer types: some cancers showed more deletions and others more insertions of various subtypes (Fig. 1). We extracted 17 indel mutational signatures (Fig. 2).

Indel signature 1 (ID1) was composed predominantly of insertions of thymine and ID2 was composed predominantly of deletions of thymine, both at long (≥5) thymine mononucleotide repeats (Fig. 2). Tens to hundreds of mutations of both signatures were found in most samples of most types of cancer, but were particularly common in colorectal, stomach, endometrial and oesophageal cancers and in diffuse large B cell lymphoma (Fig. 3). Together, ID1 and ID2 accounted for 97% and 45% of indels in hypermutated and non-hypermutated cancer genomes, respectively (Extended Data Table 2). They are probably due to slippage of either the nascent (ID1) or template strand (ID2) during DNA replication of long mononucleotide tracts.

**Extended Data Fig. 6:**
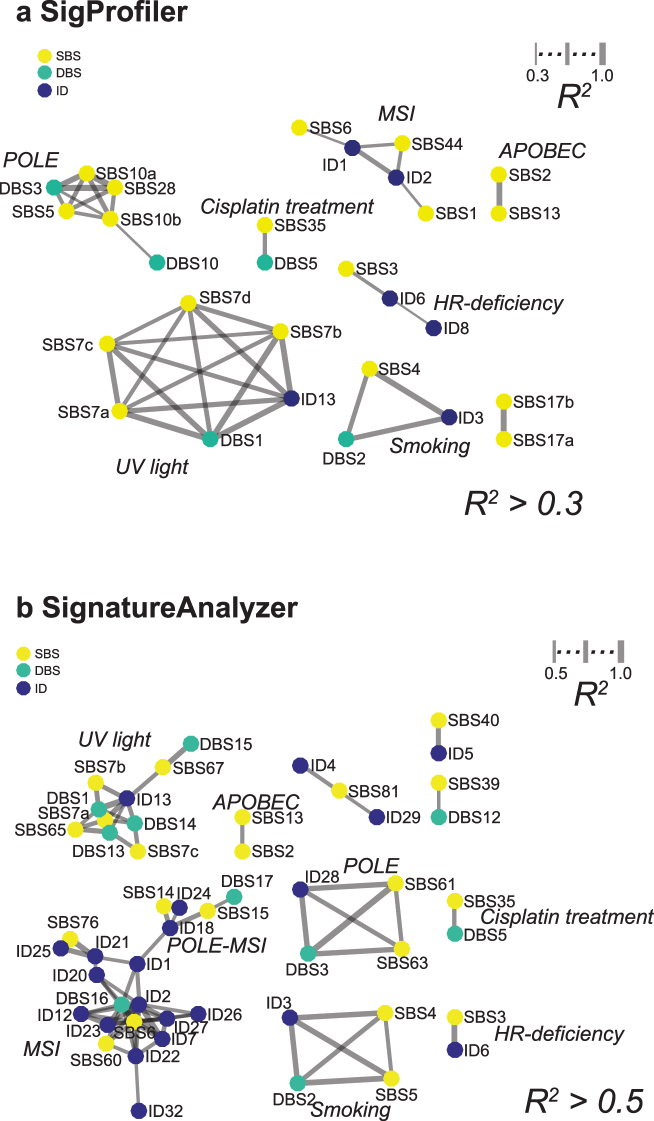
Associations between SBS, DBS and indel signature activities for SigProfiler and SignatureAnalyzer. **a**, **b**, Each node represents an SBS (light green), DBS (dark green) or indel (black) signature. Any two signatures with sample attributions that significantly correlated with *R*^2^ > 0.3 (SigProfiler) (**a**) or > 0.5 (SignatureAnalyzer) (**b**) are connected by edges. Edge widths are proportional to the strength of the correlation. Signatures with no significant correlation to any other signature above the relevant threshold are not shown. Signature locations are fit for display purposes only, and do not indicate similarity.

**Extended Data Fig. 7:**
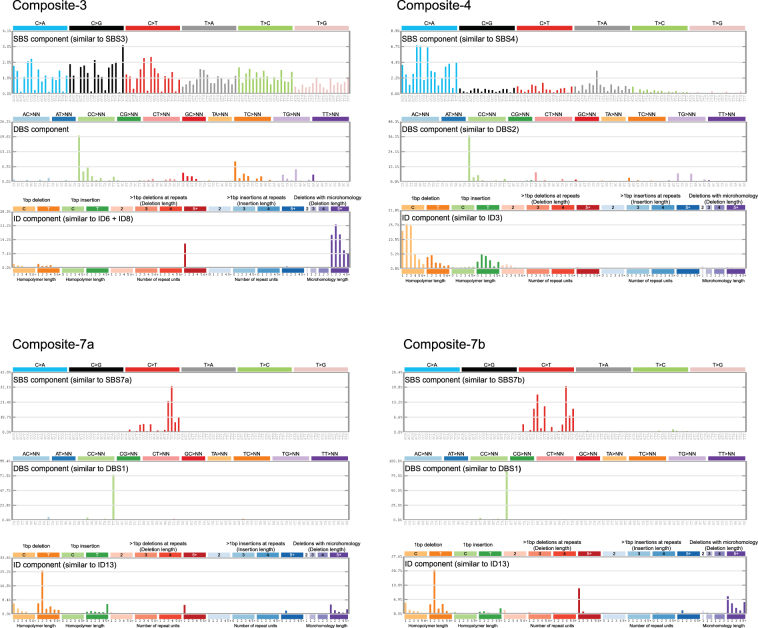
Mutational signatures extracted from the COMPOSITE feature set consisting of the concatenation of SBSs in pentanucleotide context, DBSs and indels. For each of the 4 COMPOSITE mutational signatures shown, the top panel shows the SBS signature in pentanucleotide context (1,536 mutation classes) after being collapsed to 96 SBS mutation classes, the middle panel is the co-extracted DBS signature and the bottom panel is the co-extracted indel signature. There are similarities between the DBS portion of Composite-4 and DBS2, and between the indel portion of Composite-4 and ID3; other similarities are noted in the figure.

ID3 was characterized predominantly by deletions of cytosine at short (≤5-bp long) mononucleotide cytosine repeats and exhibited hundreds of mutations in cancers of the lung, head and neck that are associated with tobacco smoking (Figs. 2, 3). There was transcriptional strand bias of mutations, with more guanine deletions than cytosine deletions on the untranscribed strands of genes, which is compatible with transcription-coupled nucleotide excision repair of damaged guanine (syn12177065 and syn12177066). The numbers of ID3 mutations positively correlated with the numbers of SBS4 and DBS2 mutations, which we have shown are associated with tobacco smoking (Extended Data Figs. 6, 7). Thus, DNA damage by components of tobacco smoke probably underlie ID3.

ID13 was characterized predominantly by deletions of thymine at thymine–thymine dinucleotides and exhibited large numbers of mutations in malignant melanomas of the skin (Figs. 2, 3). The numbers of ID13 mutations correlated with the numbers of SBS7a, SBS7b and DBS1 mutations, which we have attributed to DNA damage induced by UV light (Extended Data Figs. 6, 7). However, deletions of cytosine at cytosine–cytosine dinucleotides did not feature strongly in ID13, which may reflect the predominance of thymine compared to cytosine dimers induced by UV light^52^.

ID6 and ID8 were both characterized predominantly by ≥5-bp deletions (Fig. 2). ID6 exhibited overlapping microhomology at deletion boundaries with a mode of 2 bp (and often longer stretches) and correlated with SBS3, which we have attributed to defective homologous-recombination-based repair (Extended Data Figs. 6, 7). By contrast, ID8 deletions showed shorter or no microhomology at deletion boundaries and did not strongly correlate with SBS3. Both deletion patterns may be characteristic of DNA double-strand-break repair by non-homologous-recombination-based end-joining mechanisms and—if so—this suggests that at least two distinct forms are operative in human cancer^53^.

A small fraction of cancers exhibited very large numbers of ID1 and ID2 mutations (>10,000) (Fig. 3) (shown at https://cancer.sanger.ac.uk/cosmic/signatures/ID). These were usually accompanied by SBS6, SBS14, SBS15, SBS20, SBS21, SBS26 and/or SBS44, which are associated with deficiency in DNA mismatch repair—sometimes combined with POLE or POLD1 proofreading deficiency (SBS14 and SBS20)^35^. Occasional cases with these signatures additionally showed large numbers of indels attributed to ID7 (syn11738668), and rare samples showed large numbers of ID4, ID11, ID14, ID15, ID16 or ID17 mutations but did not show large numbers of ID1 and ID2 mutations or the SBS signatures associated with deficiency in DNA mismatch repair.

## Correlations with age

A positive correlation between age of cancer diagnosis and the number of mutations attributable to a signature suggests that the underlying mutational process has been operative (at a more or less constant rate) throughout the cell lineage from fertilized egg to cancer cell, and thus in the normal cells from which that type of cancer develops^6,54^. Confirming previous reports^6,54^, the numbers of SBS1 and SBS5 mutations correlate with age, and exhibit different rates in different types of tissue (*q* values provided in syn12030687, syn20317940 and syn12217988). SBS40 also correlated with age in multiple types of cancer, although—given its similarity to SBS5—misattribution cannot be excluded. DBS2 and DBS4 correlated with age; consistent with activity in normal cells and, when combined their profiles closely resemble the spectrum of DBS mutations found in normal mouse cells^50^. ID1, ID2, ID5 and ID8 showed correlations with age in multiple tissues. ID1 and ID2 indels are probably due to slippage at poly T repeats during DNA replication and correlated with the numbers of SBS1 substitutions, which have previously been proposed to reflect the number of mitoses a cell has experienced^6^. Thus, SBS1, ID1 and ID2 may all be generated during DNA replication at mitosis. The number of ID5 mutations correlated with the number of SBS40 mutations, and the mutational processes that underlie these two age-correlated signatures may therefore contain common components. ID8, which is predominantly composed of ≥5-bp deletions with no or 1 bp of microhomology at their boundaries, is probably due to DNA double-strand breaks repaired by a non-homologous-end-joining mechanism. The results indicate that multiple mutational processes operate in normal cells.

## Discussion

There are important constraints, limitations and assumptions in the analytic frameworks used here to characterize mutational signatures. Signatures extracted from sample sets in which multiple processes are operative remain mathematical approximations, with profiles that are potentially influenced by the mathematical approach used and other factors. For conceptual and practical simplicity, we assume that a single signature is associated with each mutational process and provide an average reference signature to represent it. However, we do not discount the possibility that further nuances and variations of signature profiles exist. We have estimated the contributions from each signature to the mutation burden in each sample. However, with increasing numbers of signatures and differences of multiple orders of magnitude in mutation burdens between some signatures, prior knowledge has helped to avoid biologically implausible results. Thus, the further development of methods for deciphering and attributing mutational signatures is warranted, ideally supported by signatures derived from experimental systems in which the causes are known. Nevertheless, signatures with many similarities and some differences can be found by different mathematical approaches, and these can be confirmed in several ways, including experimentally elucidated signatures^5,31,39,42,43,54–62^ and tumours dominated by a single signature (syn12016215).

This analysis includes most publicly available exome and whole-genome cancer sequences. Some rare or geographically restricted signatures may not have been captured, signatures conferring limited mutation burdens may have been missed and signatures of therapeutic mutagenic exposures have not been exhaustively explored. Nevertheless, it is likely that a substantial proportion of the naturally occurring mutational signatures found in human cancer have now been described. This comprehensive repertoire provides a foundation for research into the aetiologies of geographical and temporal differences in cancer incidence, the mutational processes that operate in healthy tissues and non-neoplastic disease states, clinical and public health applications of signatures and mechanistic understanding of the mutational processes that underlie carcinogenesis.

## Methods

No statistical methods were used to predetermine sample size. The experiments were not randomized and investigators were not blinded to allocation during experiments and outcome assessment.

These online methods contain an abridged description of the methodology used in the current manuscript; extensive details about the methodology we used are provided in Supplementary Note 2. Importantly, two independently developed computational frameworks (SigProfiler and SignatureAnalyzer) based on NMF were applied separately to the examined sets of mutational catalogues. SigProfiler and SignatureAnalyzer take different approaches for deciphering mutational signatures and for assigning each signature to each sample. By using two methods, we aimed to provide a perspective on the effect that different methodologies can have on the numbers of signatures generated, signature profiles and attributions. In addition to applying SigProfiler and SignatureAnalyzer to cancer data, the tools were also applied to realistic synthetic data with known solutions.

### Analysis of mutational signatures with SigProfiler

**Extended Data Fig. 8:**
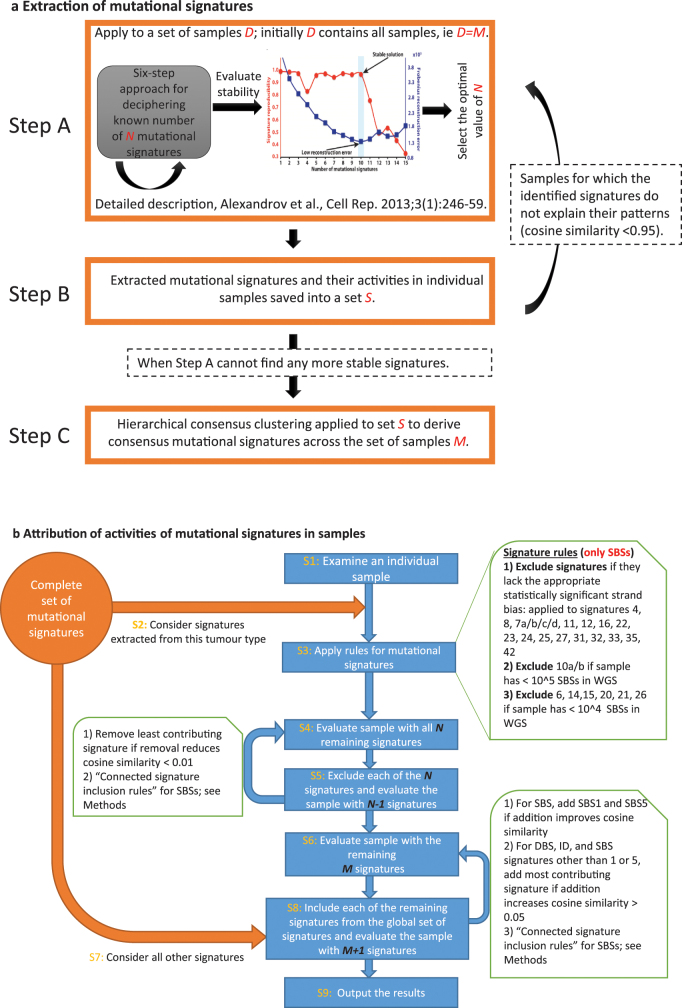
SigProfiler signature extraction and attribution. A full description is provided in Supplementary Note 2. **a**, Procedure for extracting (discovering) mutational signatures. Step A, apply the approach to a set of samples *D*; initially *D* contains all samples (that is, *D = M*). This step has previously been described in detail^17^. Step B, solution evaluation and re-iteration. Extracted mutational signatures and their activities in individual samples are saved into a set (*S*). The activity of any signature that does not increase the cosine similarity of a sample by > 0.01 was removed from the sample (assigned a value of 0). Step A is repeated for all samples for which the identified signatures do not explain their patterns (cosine similarity < 0.95). The algorithm continues to step C when step A cannot find any stable signatures. Step C, clustering of mutational signatures. Hierarchical consensus clustering was applied to the set *S* to derive the consensus mutational signatures across the set of samples *M*. **b**, Attribution of activities of mutational signatures in samples.

SigProfiler incorporates two distinct steps for identification of mutational signatures, based on the previously described methodology^6,11,17^ (Extended Data Fig. 8). The first step (SigProfilerExtraction) encompasses a hierarchical de novo extraction of mutational signatures based on somatic mutations and their immediate sequence context, and the second step (SigProfilerAttribution) focuses on accurately estimating the number of somatic mutations associated with each extracted mutational signature in each sample. SigProfilerExtraction is an extension of a previous framework for the analysis of mutational signatures^11,17^. In brief, for a given set of mutational catalogues, the algorithm deciphers a minimal set of mutational signatures that optimally explains the proportion of each mutation type and estimates the contribution of each signature to each sample. More specifically, for each NMF iteration, SigProfilerExtraction minimizes a generalized Kullback–Leibler divergence constrained for nonnegativity (Supplementary Note 2). The algorithm uses multiple NMF iterations (in most cases 1,024) to identify the matrix of mutational signatures and the matrix of the activities of these signatures, as previously described^17^. The unknown number of signatures is determined by human assessment of the stability and accuracy of solutions for a range of values, as previously described^17^. The framework is applied hierarchically to increase its ability to find mutational signatures that generate few mutations or are present in few samples.

After signatures are discovered by SigProfilerExtraction, SigProfilerAttribution estimates their contributions to individual samples. For each examined sample, the estimation algorithm involves finding the minimum of the Frobenius norm of a constrained function using a nonlinear convex optimization programming solver using the interior-point algorithm^63^. See Supplementary Note 2 and Extended Data Fig. 8b for further details.

### Analysis of mutational signatures with SignatureAnalyzer

SignatureAnalyzer uses a Bayesian variant of NMF that infers the number of signatures through the automatic relevance determination technique and delivers highly interpretable and sparse representations for both signature profiles and attributions that strike a balance between data fitting and model complexity. Further details of the actual implementation of the computational approach have previously been published^9,27,64^. SignatureAnalyzer was applied by using a two-step signature extraction strategy using 1,536 pentanucleotide contexts for SBSs, 83 indel features and 78 DBS features. In addition to the separate extraction of SBS, indel and DBS signatures, we performed a ‘COMPOSITE’ signature extraction based on all 1,697 features (1,536 SBS + 78 DBS + 83 indel). For SBSs, the 1,536 SBS COMPOSITE signatures are preferred; for DBSs and indels, the separately extracted signatures are preferred.

In step 1 of the two-step extraction process, global signature extraction was performed for the samples with a low mutation burden (*n* = 2,624). These excluded hypermutated tumours: those with putative polymerase epsilon (POLE) defects or mismatch repair defects (microsatellite instable tumours), skin tumours (which had intense UV-light mutagenesis) and one tumour with temozolomide (TMZ) exposure. Because the underlying algorithm of SignatureAnalyzer performs a stochastic search, different runs can produce different results. In step 1, we ran SignatureAnalyzer 10 times and selected the solution with the highest posterior probability. In step 2, additional signatures unique to hypermutated samples were extracted (again selecting the highest posterior probability over ten runs) while allowing all signatures found in the samples with low mutation burden, to explain some of the spectra of hypermutated samples. This approach was designed to minimize a well-known ‘signature bleeding’ effect or a bias of hyper- or ultramutated samples on the signature extraction. In addition, this approach provided information about which signatures are unique to the hypermutated samples, which was later used when attributing signatures to samples.

A similar strategy was used for signature attribution: we performed a separate attribution process for low- and hypermutated samples in all COMPOSITE, SBS, DBS and indel signatures. For downstream analyses, we preferred to use the COMPOSITE attributions for SBSs and the separately calculated attributions for DBSs and indels. Signature attribution in samples with a low mutation burden was performed separately in each tumour type (for example, Biliary–AdenoCA, Bladder–TCC, Bone–Osteosarc, and so on). Attribution was also performed separately in the combined microsatellite instable tumours (*n* = 39), POLE (*n* = 9), skin melanoma (*n* = 107) and TMZ-exposed samples (syn11738314). In both groups, signature availability (which signatures were active, or not) was primarily inferred through the automatic relevance determination process applied to the activity matrix *H* only, while fixing the signature matrix *W*. The attribution in samples with a low mutation burden was performed using only signatures found in the step 1 of the signature extraction. Two additional rules were applied in SBS signature attribution to enforce biological plausibility and minimize a signature bleeding: (i) allow SBS4 (smoking signature) only in lung, head and neck cases; and (ii) allow SBS11 (TMZ signature) in a single GBM sample. This was enforced by introducing a binary, signature-by-sample signature indicator matrix *Z* (1, allowed; 0, not allowed), which was multiplied by the *H* matrix in every multiplication update of *H*. No additional rules were applied to indel or DBS signature attributions, except that signatures found in hypermutated samples were not allowed in samples with a low mutation burden.

### Application of SigProfiler and SignatureAnalyzer to synthetic data

Our goal was to evaluate SignatureAnalyzer and SigProfiler on realistic synthetic data to identify any potential limitations of these two methods. SignatureAnalyzer and SigProfiler were tested on 11 sets of synthetic data, encompassing a total of 64,400 synthetic samples, in which known signature profiles were used to generate catalogues of synthetic mutational spectra. We operationally defined ‘realistic’ data as those based on the characteristics of either SignatureAnalyzer’s or SigProfiler’s analysis of the PCAWG genome data. SignatureAnalyzer’s reference signature profiles were based on COMPOSITE signatures, consisting of 1,536 types of strand-agnostic SBSs in pentanucleotide context, 78 types of DBSs and 83 types of small indels, for a total of 1,697 mutation types. SigProfiler’s reference analysis was based on strand-agnostic SBSs in the context of one 5′ and one 3′ base. For each test, we generated two sets of realistic data: SigProfiler-realistic (based on SigProfiler’s reference signatures and attributions) and SignatureAnalyzer-realistic (based on SignatureAnalyzer’s reference signatures and attributions), as well as two other types of data that involved using SignatureAnalyzer profiles with SigProfiler attributions and vice versa. A detailed description of each of the 11 sets of synthetic data and the results from applying SigProfiler and SignatureAnalyzer are provided in Supplementary Note 2.

### Analysis of clustered mutational signatures

Somatic SBSs were considered clustered if they had intermutational distances < 1,000 bp. More specifically, for each sample, an SBS mutational catalogue was generated for substitutions that were <1,000 bp from another substitution. Subsequently, the set of SBS mutational catalogues containing clustered mutations underwent de novo extraction of mutational signatures. Any novel mutational signature (one that was not previously observed in the complete SBS catalogues) was reported as a clustered mutational signature.

### Better separation compared to COSMIC v.2 signatures

As described in the manuscript, all mutational signatures previously reported in COSMIC v.2 were confirmed in the new set of analyses with median cosine similarity of 0.95. However, the separation between the COSMIC v.2 mutational signatures (https://cancer.sanger.ac.uk/cosmic/signatures_v2) is much worse than the separation between the mutational signatures reported here. For example, in COSMIC v.2, signatures 5 and 16 had a cosine similarity of 0.90, making them hard to distinguish from one another. By contrast, in the current analysis, SBS5 and SBS16 have a cosine similarity of 0.65. This allows us to unambiguously assign SBS5 and SBS16 to different samples. In the current analysis, the larger number of samples has allowed the reduction of bleeding between signatures and has given more unique and easily distinguishable signatures. One can evaluate the overall separation of a set of mutational signatures by examining the distribution of cosine similarities between the signatures in the set. The signatures in COSMIC v.2 had a median cosine similarity of 0.238. By contrast, the current signatures have a much lower median cosine similarity of 0.098. This twofold reduction in similarity is highly statistically significant (*P* value 9.1 × 10^−25^) and indicates a better separation between the signatures in the current analysis.

### Correlations of mutational signature activity with age

Before evaluating the association between age and the activity of a mutational signature, all outliers for both age and numbers of mutations attributed to a signature in a cancer type were removed from the data. An outlier was defined as any value outside three standard deviations from the mean value. A robust linear regression model that estimated the slope of the line and whether this slope was significantly different from zero (*F* test; *P* value < 0.05) was performed using the MATLAB function robustfit (https://www.mathworks.com/help/stats/robustfit.html) with default parameters. The *P* values from the *F* tests were corrected using the Benjamini–Hochberg procedure for false discovery rates. Results are available at syn12030687 and syn20317940.

### Reporting summary

Further information on research design is available in the Nature Research Reporting Summary linked to this paper.

## Online content

Any methods, additional references, Nature Research reporting summaries, source data, extended data, supplementary information, acknowledgements, peer review information; details of author contributions and competing interests; and statements of data and code availability are available at 10.1038/s41586-020-1943-3.

## Supplementary information

Supplementary Table This file contains Supplementary Table 1: Summary of datasets used in this paper.

Reporting Summary

Supplementary Information Supplementary Note 1: Members of the TCGA/ICGC Pan-Cancer Analysis of Whole Genomes Network.

Supplementary Information Supplementary Note 2: Additional methods details.

## Figures and Tables

**Extended Data Table 1:**
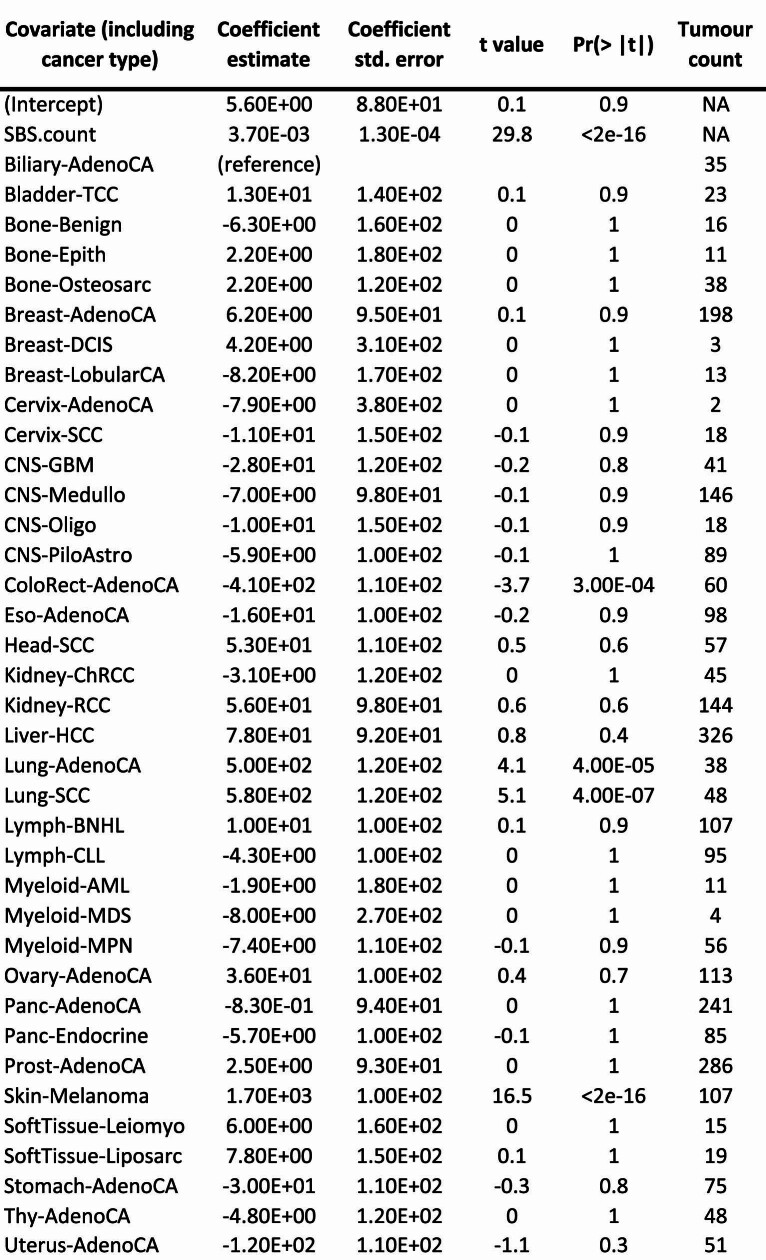
The number of DBSs is proportional to the number of SBSs, with few exceptions

The exceptions are colorectal adenocarcinoma (Colorect–AdenoCA), lung adenocarcinoma (Lung–AdenoCA), lung squamous cell carcinoma (Lung–SCC) and skin–melanoma, as analysed by the following linear regression (computed by an R function call): glm(DBS.count ~ SBS.count + Cancer.Type). This function call fits a model in which the number of DBSs depends linearly on the number of SBSs and on the cancer type. *P* values associated with the coefficients are two-sided.

**Extended Data Table 2:**
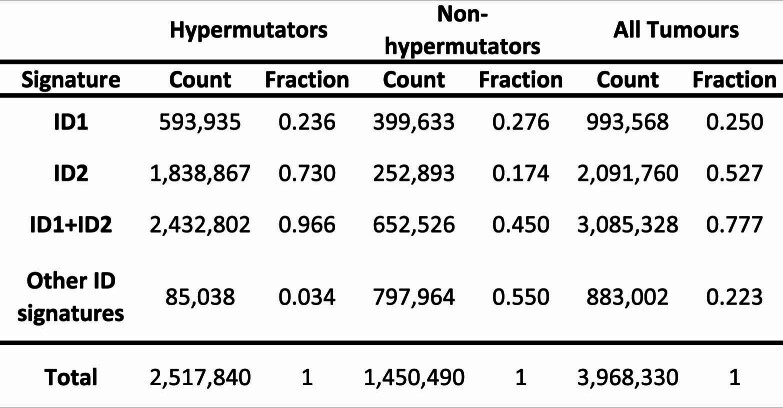
Numbers of insertion and deletion mutations due to ID1, ID2 and all other indel signatures in hypermuted and non-hypermutated tumours

## Data Availability

Somatic and germline variant calls, mutational signatures, subclonal reconstructions, transcript abundance, splice calls and other core data generated by the ICGC and TCGA PCAWG Consortium are described in ref. ^2^, and are available for download at https://dcc.icgc.org/releases/PCAWG. Additional information on accessing the data, including raw read files, can be found at https://docs.icgc.org/pcawg/data/. In accordance with the data access policies of the ICGC and TCGA projects, most molecular, clinical and specimen data are in an open tier that does not require access approval. To access information that could potentially identify participants, such as germline alleles and the underlying sequencing data, researchers will need to apply to the TCGA data access committee via dbGaP (https://dbgap.ncbi.nlm.nih.gov/aa/wga.cgi?page=login) for access to the TCGA portion of the dataset, and to the ICGC data access compliance office (http://icgc.org/daco) for the ICGC portion of the dataset. In addition, to access somatic single nucleotide variants derived from TCGA donors, researchers will also need to obtain dbGaP authorization. For each mutational signature as extracted by SigProfiler, there is a ‘vignette’ that consists of plots and a short textual description at COSMIC (available at https://cancer.sanger.ac.uk/cosmic/signatures/). Beyond the core sequence data generated by the ICGC and TCGA PCAWG Consortium, other derived datasets were generated by the research reported in this paper. These derived datasets are available at Synapse (https://www.synapse.org/#!Synapse:syn11726601/wiki/513478), and are denoted by accession numbers (synXXXXXXXX). All these datasets are mirrored at https://dcc.icgc.org/releases/PCAWG/mutational_signatures/ with full links, filenames, accession numbers and descriptions as detailed in Supplementary Table 1. These datasets include (1) CSV files comprising all catalogues of observed mutational spectra that were used as input to signature extraction (syn11801889), (2) CSV files and plots of signatures extracted by SigProfiler (syn11738306) and SignatureAnalyzer (syn11738307), (3) CSV files with estimates of the numbers of mutations generated by each signature in individual tumours (syn11804065), (4) estimates of the probability that each signature was responsible for each mutational type (for example, CTG>CAG) in individual tumours (syn11804068) and (5) synthetic test input data plus the results of tests of signature extraction (discovery) on the synthetic test data (syn18497223). All derived datasets are open access, and can be downloaded without registration or logging in.

## Code availability

SigProfiler is available both as a MATLAB framework and as a Python package. In both cases, SigProfiler is a fully functional, free and open-source tool distributed under the permissive 2-Clause BSD License. SigProfiler in MATLAB can be downloaded from: https://www.mathworks.com/matlabcentral/fileexchange/38724-sigprofiler. SigProfiler in Python can be downloaded from: https://github.com/AlexandrovLab/SigProfilerExtractor. SignatureAnalyzer code is available at https://github.com/broadinstitute/getzlab-SignatureAnalyzer (github.com). The code used to generate the synthetic data and summarize SignatureAnalyzer and SigProfiler results is open source and freely available as the SynSig package: https://github.com/steverozen/SynSig/tree/v0.2.0 under the GNU General Public License v.3.0. The core computational pipelines used by the PCAWG Consortium for alignment, quality control and variant calling are available to the public at https://dockstore.org/search?search=pcawg under the GNU General Public License v.3.0, which allows for reuse and distribution.
