# Author Correction: The repertoire of mutational signatures in human cancer

**DOI:** 10.1038/s41586-022-05600-5

**Published:** 2023-01-25

**Authors:** Ludmil B. Alexandrov, Jaegil Kim, Nicholas J. Haradhvala, Mi Ni Huang, Alvin Wei Tian Ng, Yang Wu, Arnoud Boot, Kyle R. Covington, Dmitry A. Gordenin, Erik N. Bergstrom, S. M. Ashiqul Islam, Nuria Lopez-Bigas, Leszek J. Klimczak, John R. McPherson, Sandro Morganella, Radhakrishnan Sabarinathan, David A. Wheeler, Ville Mustonen, PCAWG Mutational Signatures Working Group, Ludmil B. Alexandrov, Erik N. Bergstrom, Arnoud Boot, Paul Boutros, Kin Chan, Kyle R. Covington, Akihiro Fujimoto, Gad Getz, Dmitry A. Gordenin, Nicholas J. Haradhvala, Mi Ni Huang, S. M. Ashiqul Islam, Marat Kazanov, Jaegil Kim, Leszek J. Klimczak, Nuria Lopez-Bigas, Michael Lawrence, Iñigo Martincorena, John R. McPherson, Sandro Morganella, Ville Mustonen, Hidewaki Nakagawa, Alvin Wei Tian Ng, Paz Polak, Stephenie Prokopec, Steven A. Roberts, Steven G. Rozen, Radhakrishnan Sabarinathan, Natalie Saini, Tatsuhiro Shibata, Yuichi Shiraishi, Michael R. Stratton, Bin Tean Teh, Ignacio Vázquez-García, David A. Wheeler, Yang Wu, Fouad Yousif, Willie Yu, Gad Getz, Steven G. Rozen, Michael R. Stratton, PCAWG Consortium, Lauri A. Aaltonen, Federico Abascal, Adam Abeshouse, Hiroyuki Aburatani, David J. Adams, Nishant Agrawal, Keun Soo Ahn, Sung-Min Ahn, Hiroshi Aikata, Rehan Akbani, Kadir C. Akdemir, Hikmat Al-Ahmadie, Sultan T. Al-Sedairy, Fatima Al-Shahrour, Malik Alawi, Monique Albert, Kenneth Aldape, Ludmil B. Alexandrov, Adrian Ally, Kathryn Alsop, Eva G. Alvarez, Fernanda Amary, Samirkumar B. Amin, Brice Aminou, Ole Ammerpohl, Matthew J. Anderson, Yeng Ang, Davide Antonello, Pavana Anur, Samuel Aparicio, Elizabeth L. Appelbaum, Yasuhito Arai, Axel Aretz, Koji Arihiro, Shun-ichi Ariizumi, Joshua Armenia, Laurent Arnould, Sylvia Asa, Yassen Assenov, Gurnit Atwal, Sietse Aukema, J. Todd Auman, Miriam R. R. Aure, Philip Awadalla, Marta Aymerich, Gary D. Bader, Adrian Baez-Ortega, Matthew H. Bailey, Peter J. Bailey, Miruna Balasundaram, Saianand Balu, Pratiti Bandopadhayay, Rosamonde E. Banks, Stefano Barbi, Andrew P. Barbour, Jonathan Barenboim, Jill Barnholtz-Sloan, Hugh Barr, Elisabet Barrera, John Bartlett, Javier Bartolome, Claudio Bassi, Oliver F. Bathe, Daniel Baumhoer, Prashant Bavi, Stephen B. Baylin, Wojciech Bazant, Duncan Beardsmore, Timothy A. Beck, Sam Behjati, Andreas Behren, Beifang Niu, Cindy Bell, Sergi Beltran, Christopher Benz, Andrew Berchuck, Anke K. Bergmann, Erik N. Bergstrom, Benjamin P. Berman, Daniel M. Berney, Stephan H. Bernhart, Rameen Beroukhim, Mario Berrios, Samantha Bersani, Johanna Bertl, Miguel Betancourt, Vinayak Bhandari, Shriram G. Bhosle, Andrew V. Biankin, Matthias Bieg, Darell Bigner, Hans Binder, Ewan Birney, Michael Birrer, Nidhan K. Biswas, Bodil Bjerkehagen, Tom Bodenheimer, Lori Boice, Giada Bonizzato, Johann S. De Bono, Arnoud Boot, Moiz S. Bootwalla, Ake Borg, Arndt Borkhardt, Keith A. Boroevich, Ivan Borozan, Christoph Borst, Marcus Bosenberg, Mattia Bosio, Jacqueline Boultwood, Guillaume Bourque, Paul C. Boutros, G. Steven Bova, David T. Bowen, Reanne Bowlby, David D. L. Bowtell, Sandrine Boyault, Rich Boyce, Jeffrey Boyd, Alvis Brazma, Paul Brennan, Daniel S. Brewer, Arie B. Brinkman, Robert G. Bristow, Russell R. Broaddus, Jane E. Brock, Malcolm Brock, Annegien Broeks, Angela N. Brooks, Denise Brooks, Benedikt Brors, Søren Brunak, Timothy J. C. Bruxner, Alicia L. Bruzos, Alex Buchanan, Ivo Buchhalter, Christiane Buchholz, Susan Bullman, Hazel Burke, Birgit Burkhardt, Kathleen H. Burns, John Busanovich, Carlos D. Bustamante, Adam P. Butler, Atul J. Butte, Niall J. Byrne, Anne-Lise Børresen-Dale, Samantha J. Caesar-Johnson, Andy Cafferkey, Declan Cahill, Claudia Calabrese, Carlos Caldas, Fabien Calvo, Niedzica Camacho, Peter J. Campbell, Elias Campo, Cinzia Cantù, Shaolong Cao, Thomas E. Carey, Joana Carlevaro-Fita, Rebecca Carlsen, Ivana Cataldo, Mario Cazzola, Jonathan Cebon, Robert Cerfolio, Dianne E. Chadwick, Dimple Chakravarty, Don Chalmers, Calvin Wing Yiu Chan, Kin Chan, Michelle Chan-Seng-Yue, Vishal S. Chandan, David K. Chang, Stephen J. Chanock, Lorraine A. Chantrill, Aurélien Chateigner, Nilanjan Chatterjee, Kazuaki Chayama, Hsiao-Wei Chen, Jieming Chen, Ken Chen, Yiwen Chen, Zhaohong Chen, Andrew D. Cherniack, Jeremy Chien, Yoke-Eng Chiew, Suet-Feung Chin, Juok Cho, Sunghoon Cho, Jung Kyoon Choi, Wan Choi, Christine Chomienne, Zechen Chong, Su Pin Choo, Angela Chou, Angelika N. Christ, Elizabeth L. Christie, Eric Chuah, Carrie Cibulskis, Kristian Cibulskis, Sara Cingarlini, Peter Clapham, Alexander Claviez, Sean Cleary, Nicole Cloonan, Marek Cmero, Colin C. Collins, Ashton A. Connor, Susanna L. Cooke, Colin S. Cooper, Leslie Cope, Vincenzo Corbo, Matthew G. Cordes, Stephen M. Cordner, Isidro Cortés-Ciriano, Kyle Covington, Prue A. Cowin, Brian Craft, David Craft, Chad J. Creighton, Yupeng Cun, Erin Curley, Ioana Cutcutache, Karolina Czajka, Bogdan Czerniak, Rebecca A. Dagg, Ludmila Danilova, Maria Vittoria Davi, Natalie R. Davidson, Helen Davies, Ian J. Davis, Brandi N. Davis-Dusenbery, Kevin J. Dawson, Francisco M. De La Vega, Ricardo De Paoli-Iseppi, Timothy Defreitas, Angelo P. Dei Tos, Olivier Delaneau, John A. Demchok, Jonas Demeulemeester, German M. Demidov, Deniz Demircioğlu, Nening M. Dennis, Robert E. Denroche, Stefan C. Dentro, Nikita Desai, Vikram Deshpande, Amit G. Deshwar, Christine Desmedt, Jordi Deu-Pons, Noreen Dhalla, Neesha C. Dhani, Priyanka Dhingra, Rajiv Dhir, Anthony DiBiase, Klev Diamanti, Li Ding, Shuai Ding, Huy Q. Dinh, Luc Dirix, HarshaVardhan Doddapaneni, Nilgun Donmez, Michelle T. Dow, Ronny Drapkin, Oliver Drechsel, Ruben M. Drews, Serge Serge, Tim Dudderidge, Ana Dueso-Barroso, Andrew J. Dunford, Michael Dunn, Lewis Jonathan Dursi, Fraser R. Duthie, Ken Dutton-Regester, Jenna Eagles, Douglas F. Easton, Stuart Edmonds, Paul A. Edwards, Sandra E. Edwards, Rosalind A. Eeles, Anna Ehinger, Juergen Eils, Roland Eils, Adel El-Naggar, Matthew Eldridge, Kyle Ellrott, Serap Erkek, Georgia Escaramis, Shadrielle M. G. Espiritu, Xavier Estivill, Dariush Etemadmoghadam, Jorunn E. Eyfjord, Bishoy M. Faltas, Daiming Fan, Yu Fan, William C. Faquin, Claudiu Farcas, Matteo Fassan, Aquila Fatima, Francesco Favero, Nodirjon Fayzullaev, Ina Felau, Sian Fereday, Martin L. Ferguson, Vincent Ferretti, Lars Feuerbach, Matthew A. Field, J. Lynn Fink, Gaetano Finocchiaro, Cyril Fisher, Matthew W. Fittall, Anna Fitzgerald, Rebecca C. Fitzgerald, Adrienne M. Flanagan, Neil E. Fleshner, Paul Flicek, John A. Foekens, Kwun M. Fong, Nuno A. Fonseca, Christopher S. Foster, Natalie S. Fox, Michael Fraser, Scott Frazer, Milana Frenkel-Morgenstern, William Friedman, Joan Frigola, Catrina C. Fronick, Akihiro Fujimoto, Masashi Fujita, Masashi Fukayama, Lucinda A. Fulton, Robert S. Fulton, Mayuko Furuta, P. Andrew Futreal, Anja Füllgrabe, Stacey B. Gabriel, Steven Gallinger, Carlo Gambacorti-Passerini, Jianjiong Gao, Shengjie Gao, Levi Garraway, Øystein Garred, Erik Garrison, Dale W. Garsed, Nils Gehlenborg, Josep L. L. Gelpi, Joshy George, Daniela S. Gerhard, Clarissa Gerhauser, Jeffrey E. Gershenwald, Mark Gerstein, Moritz Gerstung, Gad Getz, Mohammed Ghori, Ronald Ghossein, Nasra H. Giama, Richard A. Gibbs, Bob Gibson, Anthony J. Gill, Pelvender Gill, Dilip D. Giri, Dominik Glodzik, Vincent J. Gnanapragasam, Maria Elisabeth Goebler, Mary J. Goldman, Carmen Gomez, Santiago Gonzalez, Abel Gonzalez-Perez, Dmitry A. Gordenin, James Gossage, Kunihito Gotoh, Ramaswamy Govindan, Dorthe Grabau, Janet S. Graham, Robert C. Grant, Anthony R. Green, Eric Green, Liliana Greger, Nicola Grehan, Sonia Grimaldi, Sean M. Grimmond, Robert L. Grossman, Adam Grundhoff, Gunes Gundem, Qianyun Guo, Manaswi Gupta, Shailja Gupta, Ivo G. Gut, Marta Gut, Jonathan Göke, Gavin Ha, Andrea Haake, David Haan, Siegfried Haas, Kerstin Haase, James E. Haber, Nina Habermann, Faraz Hach, Syed Haider, Natsuko Hama, Freddie C. Hamdy, Anne Hamilton, Mark P. Hamilton, Leng Han, George B. Hanna, Martin Hansmann, Nicholas J. Haradhvala, Olivier Harismendy, Ivon Harliwong, Arif O. Harmanci, Eoghan Harrington, Takanori Hasegawa, David Haussler, Steve Hawkins, Shinya Hayami, Shuto Hayashi, D. Neil Hayes, Stephen J. Hayes, Nicholas K. Hayward, Steven Hazell, Yao He, Allison P. Heath, Simon C. Heath, David Hedley, Apurva M. Hegde, David I. Heiman, Michael C. Heinold, Zachary Heins, Lawrence E. Heisler, Eva Hellstrom-Lindberg, Mohamed Helmy, Seong Gu Heo, Austin J. Hepperla, José María Heredia-Genestar, Carl Herrmann, Peter Hersey, Julian M. Hess, Holmfridur Hilmarsdottir, Jonathan Hinton, Satoshi Hirano, Nobuyoshi Hiraoka, Katherine A. Hoadley, Asger Hobolth, Ermin Hodzic, Jessica I. Hoell, Steve Hoffmann, Oliver Hofmann, Andrea Holbrook, Aliaksei Z. Holik, Michael A. Hollingsworth, Oliver Holmes, Robert A. Holt, Chen Hong, Eun Pyo Hong, Jongwhi H. Hong, Gerrit K. Hooijer, Henrik Hornshøj, Fumie Hosoda, Yong Hou, Volker Hovestadt, William Howat, Alan P. Hoyle, Ralph H. Hruban, Jianhong Hu, Taobo Hu, Xing Hua, Kuan-lin Huang, Mei Huang, Mi Ni Huang, Vincent Huang, Yi Huang, Wolfgang Huber, Thomas J. Hudson, Michael Hummel, Jillian A. Hung, David Huntsman, Ted R. Hupp, Jason Huse, Matthew R. Huska, Barbara Hutter, Carolyn M. Hutter, Daniel Hübschmann, Christine A. Iacobuzio-Donahue, Charles David Imbusch, Marcin Imielinski, Seiya Imoto, William B. Isaacs, Keren Isaev, Shumpei Ishikawa, Murat Iskar, S. M. Ashiqul Islam, Michael Ittmann, Sinisa Ivkovic, Jose M. G. Izarzugaza, Jocelyne Jacquemier, Valerie Jakrot, Nigel B. Jamieson, Gun Ho Jang, Se Jin Jang, Joy C. Jayaseelan, Reyka Jayasinghe, Stuart R. Jefferys, Karine Jegalian, Jennifer L. Jennings, Seung-Hyup Jeon, Lara Jerman, Yuan Ji, Wei Jiao, Peter A. Johansson, Amber L. Johns, Jeremy Johns, Rory Johnson, Todd A. Johnson, Clemency Jolly, Yann Joly, Jon G. Jonasson, Corbin D. Jones, David R. Jones, David T. W. Jones, Nic Jones, Steven J. M. Jones, Jos Jonkers, Young Seok Ju, Hartmut Juhl, Jongsun Jung, Malene Juul, Randi Istrup Juul, Sissel Juul, Natalie Jäger, Rolf Kabbe, Andre Kahles, Abdullah Kahraman, Vera B. Kaiser, Hojabr Kakavand, Sangeetha Kalimuthu, Christof von Kalle, Koo Jeong Kang, Katalin Karaszi, Beth Karlan, Rosa Karlić, Dennis Karsch, Katayoon Kasaian, Karin S. Kassahn, Hitoshi Katai, Mamoru Kato, Hiroto Katoh, Yoshiiku Kawakami, Jonathan D. Kay, Stephen H. Kazakoff, Marat D. Kazanov, Maria Keays, Electron Kebebew, Richard F. Kefford, Manolis Kellis, James G. Kench, Catherine J. Kennedy, Jules N. A. Kerssemakers, David Khoo, Vincent Khoo, Narong Khuntikeo, Ekta Khurana, Helena Kilpinen, Hark Kyun Kim, Hyung-Lae Kim, Hyung-Yong Kim, Hyunghwan Kim, Jaegil Kim, Jihoon Kim, Jong K. Kim, Youngwook Kim, Tari A. King, Wolfram Klapper, Kortine Kleinheinz, Leszek J. Klimczak, Stian Knappskog, Michael Kneba, Bartha M. Knoppers, Youngil Koh, Jan Komorowski, Daisuke Komura, Mitsuhiro Komura, Gu Kong, Marcel Kool, Jan O. Korbel, Viktoriya Korchina, Andrey Korshunov, Michael Koscher, Roelof Koster, Zsofia Kote-Jarai, Antonios Koures, Milena Kovacevic, Barbara Kremeyer, Helene Kretzmer, Markus Kreuz, Savitri Krishnamurthy, Dieter Kube, Kiran Kumar, Pardeep Kumar, Sushant Kumar, Yogesh Kumar, Ritika Kundra, Kirsten Kübler, Ralf Küppers, Jesper Lagergren, Phillip H. Lai, Peter W. Laird, Sunil R. Lakhani, Christopher M. Lalansingh, Emilie Lalonde, Fabien C. Lamaze, Adam Lambert, Eric Lander, Pablo Landgraf, Luca Landoni, Anita Langerød, Andrés Lanzós, Denis Larsimont, Erik Larsson, Mark Lathrop, Loretta M. S. Lau, Chris Lawerenz, Rita T. Lawlor, Michael S. Lawrence, Alexander J. Lazar, Ana Mijalkovic Lazic, Xuan Le, Darlene Lee, Donghoon Lee, Eunjung Alice Lee, Hee Jin Lee, Jake June-Koo Lee, Jeong-Yeon Lee, Juhee Lee, Ming Ta Michael Lee, Henry Lee-Six, Kjong-Van Lehmann, Hans Lehrach, Dido Lenze, Conrad R. Leonard, Daniel A. Leongamornlert, Ignaty Leshchiner, Louis Letourneau, Ivica Letunic, Douglas A. Levine, Lora Lewis, Tim Ley, Chang Li, Constance H. Li, Haiyan Irene Li, Jun Li, Lin Li, Shantao Li, Siliang Li, Xiaobo Li, Xiaotong Li, Xinyue Li, Yilong Li, Han Liang, Sheng-Ben Liang, Peter Lichter, Pei Lin, Ziao Lin, W. M. Linehan, Ole Christian Lingjærde, Dongbing Liu, Eric Minwei Liu, Fei-Fei Fei Liu, Fenglin Liu, Jia Liu, Xingmin Liu, Julie Livingstone, Dimitri Livitz, Naomi Livni, Lucas Lochovsky, Markus Loeffler, Georgina V. Long, Armando Lopez-Guillermo, Shaoke Lou, David N. Louis, Laurence B. Lovat, Yiling Lu, Yong-Jie Lu, Youyong Lu, Claudio Luchini, Ilinca Lungu, Xuemei Luo, Hayley J. Luxton, Andy G. Lynch, Lisa Lype, Cristina López, Carlos López-Otín, Eric Z. Ma, Yussanne Ma, Gaetan MacGrogan, Shona MacRae, Geoff Macintyre, Tobias Madsen, Kazuhiro Maejima, Andrea Mafficini, Dennis T. Maglinte, Arindam Maitra, Partha P. Majumder, Luca Malcovati, Salem Malikic, Giuseppe Malleo, Graham J. Mann, Luisa Mantovani-Löffler, Kathleen Marchal, Giovanni Marchegiani, Elaine R. Mardis, Adam A. Margolin, Maximillian G. Marin, Florian Markowetz, Julia Markowski, Jeffrey Marks, Tomas Marques-Bonet, Marco A. Marra, Luke Marsden, John W. M. Martens, Sancha Martin, Jose I. Martin-Subero, Iñigo Martincorena, Alexander Martinez-Fundichely, Yosef E. Maruvka, R. Jay Mashl, Charlie E. Massie, Thomas J. Matthew, Lucy Matthews, Erik Mayer, Simon Mayes, Michael Mayo, Faridah Mbabaali, Karen McCune, Ultan McDermott, Patrick D. McGillivray, Michael D. McLellan, John D. McPherson, John R. McPherson, Treasa A. McPherson, Samuel R. Meier, Alice Meng, Shaowu Meng, Andrew Menzies, Neil D. Merrett, Sue Merson, Matthew Meyerson, William Meyerson, Piotr A. Mieczkowski, George L. Mihaiescu, Sanja Mijalkovic, Tom Mikkelsen, Michele Milella, Linda Mileshkin, Christopher A. Miller, David K. Miller, Jessica K. Miller, Gordon B. Mills, Ana Milovanovic, Sarah Minner, Marco Miotto, Gisela Mir Arnau, Lisa Mirabello, Chris Mitchell, Thomas J. Mitchell, Satoru Miyano, Naoki Miyoshi, Shinichi Mizuno, Fruzsina Molnár-Gábor, Malcolm J. Moore, Richard A. Moore, Sandro Morganella, Quaid D. Morris, Carl Morrison, Lisle E. Mose, Catherine D. Moser, Ferran Muiños, Loris Mularoni, Andrew J. Mungall, Karen Mungall, Elizabeth A. Musgrove, Ville Mustonen, David Mutch, Francesc Muyas, Donna M. Muzny, Alfonso Muñoz, Jerome Myers, Ola Myklebost, Peter Möller, Genta Nagae, Adnan M. Nagrial, Hardeep K. Nahal-Bose, Hitoshi Nakagama, Hidewaki Nakagawa, Hiromi Nakamura, Toru Nakamura, Kaoru Nakano, Tannistha Nandi, Jyoti Nangalia, Mia Nastic, Arcadi Navarro, Fabio C. P. Navarro, David E. Neal, Gerd Nettekoven, Felicity Newell, Steven J. Newhouse, Yulia Newton, Alvin Wei Tian Ng, Anthony Ng, Jonathan Nicholson, David Nicol, Yongzhan Nie, G. Petur Nielsen, Morten Muhlig Nielsen, Serena Nik-Zainal, Michael S. Noble, Katia Nones, Paul A. Northcott, Faiyaz Notta, Brian D. O’Connor, Peter O’Donnell, Maria O’Donovan, Sarah O’Meara, Brian Patrick O’Neill, J. Robert O’Neill, David Ocana, Angelica Ochoa, Layla Oesper, Christopher Ogden, Hideki Ohdan, Kazuhiro Ohi, Lucila Ohno-Machado, Karin A. Oien, Akinyemi I. Ojesina, Hidenori Ojima, Takuji Okusaka, Larsson Omberg, Choon Kiat Ong, Stephan Ossowski, German Ott, B. F. Francis Ouellette, Christine P’ng, Marta Paczkowska, Salvatore Paiella, Chawalit Pairojkul, Marina Pajic, Qiang Pan-Hammarström, Elli Papaemmanuil, Irene Papatheodorou, Nagarajan Paramasivam, Ji Wan Park, Joong-Won Park, Keunchil Park, Kiejung Park, Peter J. Park, Joel S. Parker, Simon L. Parsons, Harvey Pass, Danielle Pasternack, Alessandro Pastore, Ann-Marie Patch, Iris Pauporté, Antonio Pea, John V. Pearson, Chandra Sekhar Pedamallu, Jakob Skou Pedersen, Paolo Pederzoli, Martin Peifer, Nathan A. Pennell, Charles M. Perou, Marc D. Perry, Gloria M. Petersen, Myron Peto, Nicholas Petrelli, Robert Petryszak, Stefan M. Pfister, Mark Phillips, Oriol Pich, Hilda A. Pickett, Todd D. Pihl, Nischalan Pillay, Sarah Pinder, Mark Pinese, Andreia V. Pinho, Esa Pitkänen, Xavier Pivot, Elena Piñeiro-Yáñez, Laura Planko, Christoph Plass, Paz Polak, Tirso Pons, Irinel Popescu, Olga Potapova, Aparna Prasad, Shaun R. Preston, Manuel Prinz, Antonia L. Pritchard, Stephenie D. Prokopec, Elena Provenzano, Xose S. Puente, Sonia Puig, Montserrat Puiggròs, Sergio Pulido-Tamayo, Gulietta M. Pupo, Colin A. Purdie, Michael C. Quinn, Raquel Rabionet, Janet S. Rader, Bernhard Radlwimmer, Petar Radovic, Benjamin Raeder, Keiran M. Raine, Manasa Ramakrishna, Kamna Ramakrishnan, Suresh Ramalingam, Benjamin J. Raphael, W. Kimryn Rathmell, Tobias Rausch, Guido Reifenberger, Jüri Reimand, Jorge Reis-Filho, Victor Reuter, Iker Reyes-Salazar, Matthew A. Reyna, Sheila M. Reynolds, Esther Rheinbay, Yasser Riazalhosseini, Andrea L. Richardson, Julia Richter, Matthew Ringel, Markus Ringnér, Yasushi Rino, Karsten Rippe, Jeffrey Roach, Lewis R. Roberts, Nicola D. Roberts, Steven A. Roberts, A. Gordon Robertson, Alan J. Robertson, Javier Bartolomé Rodriguez, Bernardo Rodriguez-Martin, F. Germán Rodríguez-González, Michael H. A. Roehrl, Marius Rohde, Hirofumi Rokutan, Gilles Romieu, Ilse Rooman, Tom Roques, Daniel Rosebrock, Mara Rosenberg, Philip C. Rosenstiel, Andreas Rosenwald, Edward W. Rowe, Romina Royo, Steven G. Rozen, Yulia Rubanova, Mark A. Rubin, Carlota Rubio-Perez, Vasilisa A. Rudneva, Borislav C. Rusev, Andrea Ruzzenente, Gunnar Rätsch, Radhakrishnan Sabarinathan, Veronica Y. Sabelnykova, Sara Sadeghi, S. Cenk Sahinalp, Natalie Saini, Mihoko Saito-Adachi, Gordon Saksena, Adriana Salcedo, Roberto Salgado, Leonidas Salichos, Richard Sallari, Charles Saller, Roberto Salvia, Michelle Sam, Jaswinder S. Samra, Francisco Sanchez-Vega, Chris Sander, Grant Sanders, Rajiv Sarin, Iman Sarrafi, Aya Sasaki-Oku, Torill Sauer, Guido Sauter, Robyn P. M. Saw, Maria Scardoni, Christopher J. Scarlett, Aldo Scarpa, Ghislaine Scelo, Dirk Schadendorf, Jacqueline E. Schein, Markus B. Schilhabel, Matthias Schlesner, Thorsten Schlomm, Heather K. Schmidt, Sarah-Jane Schramm, Stefan Schreiber, Nikolaus Schultz, Steven E. Schumacher, Roland F. Schwarz, Richard A. Scolyer, David Scott, Ralph Scully, Raja Seethala, Ayellet V. Segre, Iris Selander, Colin A. Semple, Yasin Senbabaoglu, Subhajit Sengupta, Elisabetta Sereni, Stefano Serra, Dennis C. Sgroi, Mark Shackleton, Nimish C. Shah, Sagedeh Shahabi, Catherine A. Shang, Ping Shang, Ofer Shapira, Troy Shelton, Ciyue Shen, Hui Shen, Rebecca Shepherd, Ruian Shi, Yan Shi, Yu-Jia Shiah, Tatsuhiro Shibata, Juliann Shih, Eigo Shimizu, Kiyo Shimizu, Seung Jun Shin, Yuichi Shiraishi, Tal Shmaya, Ilya Shmulevich, Solomon I. Shorser, Charles Short, Raunak Shrestha, Suyash S. Shringarpure, Craig Shriver, Shimin Shuai, Nikos Sidiropoulos, Reiner Siebert, Anieta M. Sieuwerts, Lina Sieverling, Sabina Signoretti, Katarzyna O. Sikora, Michele Simbolo, Ronald Simon, Janae V. Simons, Jared T. Simpson, Peter T. Simpson, Samuel Singer, Nasa Sinnott-Armstrong, Payal Sipahimalani, Tara J. Skelly, Marcel Smid, Jaclyn Smith, Karen Smith-McCune, Nicholas D. Socci, Heidi J. Sofia, Matthew G. Soloway, Lei Song, Anil K. Sood, Sharmila Sothi, Christos Sotiriou, Cameron M. Soulette, Paul N. Span, Paul T. Spellman, Nicola Sperandio, Andrew J. Spillane, Oliver Spiro, Jonathan Spring, Johan Staaf, Peter F. Stadler, Peter Staib, Stefan G. Stark, Lucy Stebbings, Ólafur Andri Stefánsson, Oliver Stegle, Lincoln D. Stein, Alasdair Stenhouse, Chip Stewart, Stephan Stilgenbauer, Miranda D. Stobbe, Michael R. Stratton, Jonathan R. Stretch, Adam J. Struck, Joshua M. Stuart, Henk G. Stunnenberg, Hong Su, Xiaoping Su, Ren X. Sun, Stephanie Sungalee, Hana Susak, Akihiro Suzuki, Fred Sweep, Monika Szczepanowski, Holger Sültmann, Takashi Yugawa, Angela Tam, David Tamborero, Benita Kiat Tee Tan, Donghui Tan, Patrick Tan, Hiroko Tanaka, Hirokazu Taniguchi, Tomas J. Tanskanen, Maxime Tarabichi, Roy Tarnuzzer, Patrick Tarpey, Morgan L. Taschuk, Kenji Tatsuno, Simon Tavaré, Darrin F. Taylor, Amaro Taylor-Weiner, Jon W. Teague, Bin Tean Teh, Varsha Tembe, Javier Temes, Kevin Thai, Sarah P. Thayer, Nina Thiessen, Gilles Thomas, Sarah Thomas, Alan Thompson, Alastair M. Thompson, John F. F. Thompson, R. Houston Thompson, Heather Thorne, Leigh B. Thorne, Adrian Thorogood, Grace Tiao, Nebojsa Tijanic, Lee E. Timms, Roberto Tirabosco, Marta Tojo, Stefania Tommasi, Christopher W. Toon, Umut H. Toprak, David Torrents, Giampaolo Tortora, Jörg Tost, Yasushi Totoki, David Townend, Nadia Traficante, Isabelle Treilleux, Jean-Rémi Trotta, Lorenz H. P. Trümper, Ming Tsao, Tatsuhiko Tsunoda, Jose M. C. Tubio, Olga Tucker, Richard Turkington, Daniel J. Turner, Andrew Tutt, Masaki Ueno, Naoto T. Ueno, Christopher Umbricht, Husen M. Umer, Timothy J. Underwood, Lara Urban, Tomoko Urushidate, Tetsuo Ushiku, Liis Uusküla-Reimand, Alfonso Valencia, David J. Van Den Berg, Steven Van Laere, Peter Van Loo, Erwin G. Van Meir, Gert G. Van den Eynden, Theodorus Van der Kwast, Naveen Vasudev, Miguel Vazquez, Ravikiran Vedururu, Umadevi Veluvolu, Shankar Vembu, Lieven P. C. Verbeke, Peter Vermeulen, Clare Verrill, Alain Viari, David Vicente, Caterina Vicentini, K. VijayRaghavan, Juris Viksna, Ricardo E. Vilain, Izar Villasante, Anne Vincent-Salomon, Tapio Visakorpi, Douglas Voet, Paresh Vyas, Ignacio Vázquez-García, Nick M. Waddell, Nicola Waddell, Claes Wadelius, Lina Wadi, Rabea Wagener, Jeremiah A. Wala, Jian Wang, Jiayin Wang, Linghua Wang, Qi Wang, Wenyi Wang, Yumeng Wang, Zhining Wang, Paul M. Waring, Hans-Jörg Warnatz, Jonathan Warrell, Anne Y. Warren, Sebastian M. Waszak, David C. Wedge, Dieter Weichenhan, Paul Weinberger, John N. Weinstein, Joachim Weischenfeldt, Daniel J. Weisenberger, Ian Welch, Michael C. Wendl, Johannes Werner, Justin P. Whalley, David A. Wheeler, Hayley C. Whitaker, Dennis Wigle, Matthew D. Wilkerson, Ashley Williams, James S. Wilmott, Gavin W. Wilson, Julie M. Wilson, Richard K. Wilson, Boris Winterhoff, Jeffrey A. Wintersinger, Maciej Wiznerowicz, Stephan Wolf, Bernice H. Wong, Tina Wong, Winghing Wong, Youngchoon Woo, Scott Wood, Bradly G. Wouters, Adam J. Wright, Derek W. Wright, Mark H. Wright, Chin-Lee Wu, Dai-Ying Wu, Guanming Wu, Jianmin Wu, Kui Wu, Yang Wu, Zhenggang Wu, Liu Xi, Tian Xia, Qian Xiang, Xiao Xiao, Rui Xing, Heng Xiong, Qinying Xu, Yanxun Xu, Hong Xue, Shinichi Yachida, Sergei Yakneen, Rui Yamaguchi, Takafumi N. Yamaguchi, Masakazu Yamamoto, Shogo Yamamoto, Hiroki Yamaue, Fan Yang, Huanming Yang, Jean Y. Yang, Liming Yang, Lixing Yang, Shanlin Yang, Tsun-Po Yang, Yang Yang, Xiaotong Yao, Marie-Laure Yaspo, Lucy Yates, Christina Yau, Chen Ye, Kai Ye, Venkata D. Yellapantula, Christopher J. Yoon, Sung-Soo Yoon, Fouad Yousif, Jun Yu, Kaixian Yu, Willie Yu, Yingyan Yu, Ke Yuan, Yuan Yuan, Denis Yuen, Christina K. Yung, Olga Zaikova, Jorge Zamora, Marc Zapatka, Jean C. Zenklusen, Thorsten Zenz, Nikolajs Zeps, Cheng-Zhong Zhang, Fan Zhang, Hailei Zhang, Hongwei Zhang, Hongxin Zhang, Jiashan Zhang, Jing Zhang, Junjun Zhang, Xiuqing Zhang, Xuanping Zhang, Yan Zhang, Zemin Zhang, Zhongming Zhao, Liangtao Zheng, Xiuqing Zheng, Wanding Zhou, Yong Zhou, Bin Zhu, Hongtu Zhu, Jingchun Zhu, Shida Zhu, Lihua Zou, Xueqing Zou, Anna deFazio, Nicholas van As, Carolien H. M. van Deurzen, Marc J. van de Vijver, L. van’t Veer, Christian von Mering

**Affiliations:** 1 Department of Cellular and Molecular Medicine, Department of Bioengineering, Moores Cancer Center, University of California, San Diego, CA USA; 2 Broad Institute of MIT and Harvard, Cambridge, MA USA; 3 Center for Cancer Research, Massachusetts General Hospital, Boston, MA USA; 4 Programme in Cancer & Stem Cell Biology, Duke-NUS Medical School, Singapore, Singapore; 5 Centre for Computational Biology, Duke-NUS Medical School, Singapore, Singapore; 6 Human Genome Sequencing Center, Baylor College of Medicine, Houston, TX USA; 7 Dan L. Duncan Cancer Center, Baylor College of Medicine, Houston, TX USA; 8 Genome Integrity and Structural Biology Laboratory, National Institute of Environmental Health Sciences (NIEHS), Durham, NC USA; 9 Institute for Research in Biomedicine (IRB Barcelona), The Barcelona Institute of Science and Technology, Barcelona, Spain; 10 Research Program on Biomedical Informatics, Universitat Pompeu Fabra, Barcelona, Spain; 11 Institució Catalana de Recerca i Estudis Avançats (ICREA), Barcelona, Spain; 12 Integrative Bioinformatics Support Group, National Institute of Environmental Health Sciences (NIEHS), Durham, NC USA; 13 Wellcome Sanger Institute, Hinxton, UK; 14 National Centre for Biological Sciences, Tata Institute of Fundamental Research, Bangalore, India; 15 Institute for Research in Biomedicine (IRB Barcelona), The Barcelona Institute of Science and Technology, Barcelona, Spain; 16 Department of Molecular and Human Genetics, Baylor College of Medicine, Houston, TX USA; 17 Department of Computer Science, University of Helsinki, Helsinki, Finland; 18 Organismal and Evolutionary Biology Research Programme, University of Helsinki, Helsinki, Finland; 19 Institute of Biotechnology, University of Helsinki, Helsinki, Finland; 21 Department of Pathology, Massachusetts General Hospital, Boston, MA USA; 22 Harvard Medical School, Boston, MA USA; 23 SingHealth, Duke-NUS Institute of Precision Medicine, National Heart Centre Singapore, Singapore, Singapore; 25 Department of Medical Biophysics, University of Toronto, Toronto, Ontario Canada; 26 University of California Los Angeles, Los Angeles, CA USA; 27 Computational Biology Program, Ontario Institute for Cancer Research, Toronto, Ontario Canada; 28 Department of Pharmacology, University of Toronto, Toronto, Ontario Canada; 29 Department of Biochemistry, Microbiology and Immunology, Faculty of Medicine, University of Ottawa, Ottawa, Ontario Canada; 30 RIKEN Center for Integrative Medical Sciences, Yokohama, Japan; 31 A. A. Kharkevich Institute of Information Transmission Problems, Moscow, Russia; 32 Dmitry Rogachev National Research Center of Pediatric Hematology, Oncology and Immunology, Moscow, Russia; 33 Skolkovo Institute of Science and Technology, Moscow, Russia; 34 Laboratory for Medical Science Mathematics, RIKEN Center for Integrative Medical Sciences, Yokohama, Japan; 35 Massachusetts General Hospital, Boston, MA USA; 36 School of Molecular Biosciences, Washington State University, Pullman, WA USA; 37 Center for Reproductive Biology, Washington State University, Pullman, WA USA; 38 Laboratory of Molecular Medicine, Human Genome Center, The Institute of Medical Science, The University of Tokyo, Tokyo, Japan; 39 Division of Cancer Genomics, National Cancer Center Research Institute, Tokyo, Japan; 40 The Institute of Medical Science, The University of Tokyo, Tokyo, Japan; 41 Cancer Science Institute of Singapore, National University of Singapore, Singapore, Singapore; 42 Institute of Molecular and Cell Biology, Singapore, Singapore; 43 Laboratory of Cancer Epigenome, Division of Medical Science, National Cancer Centre Singapore, Singapore, Singapore; 44 Department of Epidemiology and Biostatistics, Memorial Sloan Kettering Cancer Center, New York, NY USA; 45 Department of Statistics, Columbia University, New York, NY USA; 46 Department of Applied Mathematics and Theoretical Physics, Centre for Mathematical Sciences, University of Cambridge, Cambridge, UK; 200 Applied Tumor Genomics Research Program, Research Programs Unit, University of Helsinki, Helsinki, Finland; 201 Wellcome Sanger Institute, Wellcome Genome Campus, Hinxton, UK; 202 Memorial Sloan Kettering Cancer Center, New York, NY USA; 203 Genome Science Division, Research Center for Advanced Science and Technology, University of Tokyo, Tokyo, Japan; 204 Department of Surgery, University of Chicago, Chicago, IL USA; 205 Department of Surgery, Division of Hepatobiliary and Pancreatic Surgery, School of Medicine, Keimyung University Dongsan Medical Center, Daegu, South Korea; 206 Department of Oncology, Gil Medical Center, Gachon University, Incheon, South Korea; 207 Hiroshima University, Hiroshima, Japan; 208 Department of Bioinformatics and Computational Biology, The University of Texas MD Anderson Cancer Center, Houston, TX USA; 209 University of Texas MD Anderson Cancer Center, Houston, TX USA; 210 King Faisal Specialist Hospital and Research Centre, Al Maather, Riyadh, Saudi Arabia; 211 Bioinformatics Unit, Spanish National Cancer Research Centre (CNIO), Madrid, Spain; 212 Bioinformatics Core Facility, University Medical Center Hamburg, Hamburg, Germany; 213 Heinrich Pette Institute, Leibniz Institute for Experimental Virology, Hamburg, Germany; 214 Ontario Tumour Bank, Ontario Institute for Cancer Research, Toronto, ON Canada; 215 Department of Pathology, The University of Texas MD Anderson Cancer Center, Houston, TX USA; 216 Laboratory of Pathology, Center for Cancer Research, National Cancer Institute, Bethesda, MD USA; 217 Department of Cellular and Molecular Medicine and Department of Bioengineering, University of California San Diego, La Jolla, CA USA; 218 UC San Diego Moores Cancer Center, San Diego, CA USA; 219 Canada’s Michael Smith Genome Sciences Centre, BC Cancer, Vancouver, BC Canada; 220 Sir Peter MacCallum Department of Oncology, Peter MacCallum Cancer Centre, University of Melbourne, Melbourne, VIC Australia; 221 Centre for Research in Molecular Medicine and Chronic Diseases (CiMUS), Universidade de Santiago de Compostela, Santiago de Compostela, Spain; 222 Department of Zoology, Genetics and Physical Anthropology, (CiMUS), Universidade de Santiago de Compostela, Santiago de Compostela, Spain; 223 The Biomedical Research Centre (CINBIO), Universidade de Vigo, Vigo, Spain; 224 Royal National Orthopaedic Hospital - Bolsover, London, UK; 225 Department of Genomic Medicine, The University of Texas MD Anderson Cancer Center, Houston, TX USA; 226 Quantitative and Computational Biosciences Graduate Program, Baylor College of Medicine, Houston, TX USA; 227 The Jackson Laboratory for Genomic Medicine, Farmington, CT USA; 228 Genome Informatics Program, Ontario Institute for Cancer Research, Toronto, ON Canada; 229 Institute of Human Genetics, Christian-Albrechts-University, Kiel, Germany; 230 Institute of Human Genetics, Ulm University and Ulm University Medical Center, Ulm, Germany; 231 Queensland Centre for Medical Genomics, Institute for Molecular Bioscience, University of Queensland, St. Lucia, Brisbane, QLD Australia; 232 Salford Royal NHS Foundation Trust, Salford, UK; 233 Department of Surgery, Pancreas Institute, University and Hospital Trust of Verona, Verona, Italy; 234 Molecular and Medical Genetics, OHSU Knight Cancer Institute, Oregon Health and Science University, Portland, OR USA; 235 Department of Molecular Oncology, BC Cancer Research Centre, Vancouver, BC Canada; 236 The McDonnell Genome Institute at Washington University, St. Louis, MO USA; 237 University College London, London, UK; 238 Division of Cancer Genomics, National Cancer Center Research Institute, National Cancer Center, Tokyo, Japan; 239 DLR Project Management Agency, Bonn, Germany; 240 Tokyo Women’s Medical University, Tokyo, Japan; 241 Center for Molecular Oncology, Memorial Sloan Kettering Cancer Center, New York, NY USA; 242 Los Alamos National Laboratory, Los Alamos, NM USA; 243 Department of Pathology, University Health Network, Toronto General Hospital, Toronto, ON Canada; 244 Nottingham University Hospitals NHS Trust, Nottingham, UK; 245 Epigenomics and Cancer Risk Factors, German Cancer Research Center (DKFZ), Heidelberg, Germany; 246 Computational Biology Program, Ontario Institute for Cancer Research, Toronto, ON Canada; 247 Department of Molecular Genetics, University of Toronto, Toronto, ON Canada; 248 Vector Institute, Toronto, ON Canada; 249 Hematopathology Section, Institute of Pathology, Christian-Albrechts-University, Kiel, Germany; 250 Department of Pathology and Laboratory Medicine, School of Medicine, University of North Carolina at Chapel Hill, Chapel Hill, NC USA; 251 Department of Cancer Genetics, Institute for Cancer Research, Oslo University Hospital, The Norwegian Radium Hospital, Oslo, Norway; 252 Pathology, Hospital Clinic, Institut d’Investigacions Biomèdiques August Pi i Sunyer (IDIBAPS), University of Barcelona, Barcelona, Spain; 253 Department of Veterinary Medicine, Transmissible Cancer Group, University of Cambridge, Cambridge, UK; 254 Alvin J. Siteman Cancer Center, Washington University School of Medicine, St. Louis, MO USA; 255 Wolfson Wohl Cancer Research Centre, Institute of Cancer Sciences, University of Glasgow, Glasgow, UK; 256 Lineberger Comprehensive Cancer Center, University of North Carolina at Chapel Hill, Chapel Hill, NC USA; 257 Broad Institute of MIT and Harvard, Cambridge, MA USA; 258 Dana-Farber/Boston Children’s Cancer and Blood Disorders Center, Boston, MA USA; 259 Department of Pediatrics, Harvard Medical School, Boston, MA USA; 260 Leeds Institute of Medical Research @ St. James’s, University of Leeds, St. James’s University Hospital, Leeds, UK; 261 Department of Pathology and Diagnostics, University and Hospital Trust of Verona, Verona, Italy; 262 Department of Surgery, Princess Alexandra Hospital, Brisbane, QLD Australia; 263 Surgical Oncology Group, Diamantina Institute, University of Queensland, Brisbane, QLD Australia; 264 Department of Population and Quantitative Health Sciences, Case Western Reserve University School of Medicine, Cleveland, OH USA; 265 Research Health Analytics and Informatics, University Hospitals Cleveland Medical Center, Cleveland, OH USA; 266 Gloucester Royal Hospital, Gloucester, UK; 267 European Molecular Biology Laboratory, European Bioinformatics Institute (EMBL-EBI), Cambridge, UK; 268 Diagnostic Development, Ontario Institute for Cancer Research, Toronto, ON Canada; 269 Barcelona Supercomputing Center (BSC), Barcelona, Spain; 270 Arnie Charbonneau Cancer Institute, University of Calgary, Calgary, AB Canada; 271 Departments of Surgery and Oncology, University of Calgary, Calgary, AB Canada; 272 Department of Pathology, Oslo University Hospital, The Norwegian Radium Hospital, Oslo, Norway; 273 PanCuRx Translational Research Initiative, Ontario Institute for Cancer Research, Toronto, ON Canada; 274 Department of Oncology, Sidney Kimmel Comprehensive Cancer Center at Johns Hopkins University School of Medicine, Baltimore, MD USA; 275 University Hospital Southampton NHS Foundation Trust, Southampton, UK; 276 Royal Stoke University Hospital, Stoke-on-Trent, UK; 277 Genome Sequence Informatics, Ontario Institute for Cancer Research, Toronto, ON Canada; 278 Human Longevity Inc, San Diego, CA USA; 279 Olivia Newton-John Cancer Research Institute, La Trobe University, Heidelberg, VIC Australia; 280 Computer Network Information Center, Chinese Academy of Sciences, Beijing, China; 281 Genome Canada, Ottawa, ON Canada; 282 CNAG-CRG, Centre for Genomic Regulation (CRG), Barcelona Institute of Science and Technology (BIST), Barcelona, Spain; 283 Universitat Pompeu Fabra (UPF), Barcelona, Spain; 284 Buck Institute for Research on Aging, Novato, CA USA; 285 Duke University Medical Center, Durham, NC USA; 286 Department of Human Genetics, Hannover Medical School, Hannover, Germany; 287 Center for Bioinformatics and Functional Genomics, Cedars-Sinai Medical Center, Los Angeles, CA USA; 288 Department of Biomedical Sciences, Cedars-Sinai Medical Center, Los Angeles, CA USA; 289 The Hebrew University Faculty of Medicine, Jerusalem, Israel; 290 Barts Cancer Institute, Barts and the London School of Medicine and Dentistry, Queen Mary University of London, London, UK; 291 Department of Computer Science, Bioinformatics Group, University of Leipzig, Leipzig, Germany; 292 Interdisciplinary Center for Bioinformatics, University of Leipzig, Leipzig, Germany; 293 Transcriptome Bioinformatics, LIFE Research Center for Civilization Diseases, University of Leipzig, Leipzig, Germany; 294 Department of Medical Oncology, Dana-Farber Cancer Institute, Boston, MA USA; 295 Department of Cancer Biology, Dana-Farber Cancer Institute, Boston, MA USA; 296 Harvard Medical School, Boston, MA USA; 297 USC Norris Comprehensive Cancer Center, University of Southern California, Los Angeles, CA USA; 298 Department of Diagnostics and Public Health, University and Hospital Trust of Verona, Verona, Italy; 299 Department of Mathematics, Aarhus University, Aarhus, Denmark; 300 Department of Molecular Medicine (MOMA), Aarhus University Hospital, Aarhus N, Denmark; 301 Instituto Carlos Slim de la Salud, Mexico City, Mexico; 302 Department of Medical Biophysics, University of Toronto, Toronto, ON Canada; 303 Cancer Division, Garvan Institute of Medical Research, Kinghorn Cancer Centre, University of New South Wales (UNSW Sydney), Sydney, NSW Australia; 304 South Western Sydney Clinical School, Faculty of Medicine, University of New South Wales (UNSW Sydney), Liverpool, NSW Australia; 305 West of Scotland Pancreatic Unit, Glasgow Royal Infirmary, Glasgow, UK; 306 Center for Digital Health, Berlin Institute of Health and Charitè - Universitätsmedizin Berlin, Berlin, Germany; 307 Heidelberg Center for Personalized Oncology (DKFZ-HIPO), German Cancer Research Center (DKFZ), Heidelberg, Germany; 308 The Preston Robert Tisch Brain Tumor Center, Duke University Medical Center, Durham, NC USA; 309 Massachusetts General Hospital, Boston, MA USA; 310 National Institute of Biomedical Genomics, Kalyani, West Bengal India; 311 Institute of Clinical Medicine and Institute of Oral Biology, University of Oslo, Oslo, Norway; 312 University of North Carolina at Chapel Hill, Chapel Hill, NC USA; 313 ARC-Net Centre for Applied Research on Cancer, University and Hospital Trust of Verona, Verona, Italy; 314 The Institute of Cancer Research, London, UK; 315 Centre for Computational Biology, Duke-NUS Medical School, Singapore, Singapore; 316 Programme in Cancer and Stem Cell Biology, Duke-NUS Medical School, Singapore, Singapore; 317 Division of Oncology and Pathology, Department of Clinical Sciences Lund, Lund University, Lund, Sweden; 318 Department of Pediatric Oncology, Hematology and Clinical Immunology, Heinrich-Heine-University, Düsseldorf, Germany; 319 Laboratory for Medical Science Mathematics, RIKEN Center for Integrative Medical Sciences, Yokohama, Japan; 320 RIKEN Center for Integrative Medical Sciences, Yokohama, Japan; 321 Department of Internal Medicine/Hematology, Friedrich-Ebert-Hospital, Neumünster, Germany; 322 Departments of Dermatology and Pathology, Yale University, New Haven, CT USA; 323 Centre for Genomic Regulation (CRG), The Barcelona Institute of Science and Technology, Barcelona, Spain; 324 Radcliffe Department of Medicine, University of Oxford, Oxford, UK; 325 Canadian Center for Computational Genomics, McGill University, Montreal, QC Canada; 326 Department of Human Genetics, McGill University, Montreal, QC Canada; 327 Department of Human Genetics, University of California Los Angeles, Los Angeles, CA USA; 328 Department of Pharmacology, University of Toronto, Toronto, ON Canada; 329 Faculty of Medicine and Health Technology, Tampere University and Tays Cancer Center, Tampere University Hospital, Tampere, Finland; 330 Haematology, Leeds Teaching Hospitals NHS Trust, Leeds, UK; 331 Translational Research and Innovation, Centre Léon Bérard, Lyon, France; 332 Fox Chase Cancer Center, Philadelphia, PA USA; 333 International Agency for Research on Cancer, World Health Organization, Lyon, France; 334 Earlham Institute, Norwich, UK; 335 Norwich Medical School, University of East Anglia, Norwich, UK; 336 Department of Molecular Biology, Faculty of Science, Radboud Institute for Molecular Life Sciences, Radboud University, Nijmegen, HB The Netherlands; 337 CRUK Manchester Institute and Centre, Manchester, UK; 338 Department of Radiation Oncology, University of Toronto, Toronto, ON Canada; 339 Division of Cancer Sciences, Manchester Cancer Research Centre, University of Manchester, Manchester, UK; 340 Radiation Medicine Program, Princess Margaret Cancer Centre, Toronto, ON Canada; 341 Department of Pathology, Brigham and Women’s Hospital, Harvard Medical School, Boston, MA USA; 342 Department of Surgery, Division of Thoracic Surgery, The Johns Hopkins University School of Medicine, Baltimore, MD USA; 343 Division of Molecular Pathology, The Netherlands Cancer Institute, Oncode Institute, Amsterdam, CX The Netherlands; 344 Department of Biomolecular Engineering, University of California Santa Cruz, Santa Cruz, CA USA; 345 UC Santa Cruz Genomics Institute, University of California Santa Cruz, Santa Cruz, CA USA; 346 Division of Applied Bioinformatics, German Cancer Research Center (DKFZ), Heidelberg, Germany; 347 German Cancer Consortium (DKTK), German Cancer Research Center (DKFZ), Heidelberg, Germany; 348 National Center for Tumor Diseases (NCT) Heidelberg, Heidelberg, Germany; 349 Center for Biological Sequence Analysis, Department of Bio and Health Informatics, Technical University of Denmark, Lyngby, Denmark; 350 Novo Nordisk Foundation Center for Protein Research, University of Copenhagen, Copenhagen, Denmark; 351 Institute for Molecular Bioscience, University of Queensland, St. Lucia, Brisbane, QLD Australia; 352 Biomedical Engineering, Oregon Health and Science University, Portland, OR USA; 353 Division of Theoretical Bioinformatics, German Cancer Research Center (DKFZ), Heidelberg, Germany; 354 Institute of Pharmacy and Molecular Biotechnology and BioQuant, Heidelberg University, Heidelberg, Germany; 355 Federal Ministry of Education and Research, Berlin, Germany; 356 Melanoma Institute Australia, University of Sydney, Sydney, NSW Australia; 357 Pediatric Hematology and Oncology, University Hospital Muenster, Muenster, Germany; 358 Department of Pathology, Johns Hopkins University School of Medicine, Baltimore, MD USA; 359 McKusick-Nathans Institute of Genetic Medicine, Sidney Kimmel Comprehensive Cancer Center at Johns Hopkins University School of Medicine, Baltimore, MD USA; 360 Foundation Medicine, Inc, Cambridge, MA USA; 361 Department of Biomedical Data Science, Stanford University School of Medicine, Stanford, CA USA; 362 Department of Genetics, Stanford University School of Medicine, Stanford, CA USA; 363 Bakar Computational Health Sciences Institute and Department of Pediatrics, University of California, San Francisco, CA USA; 364 Institute of Clinical Medicine, Faculty of Medicine, University of Oslo, Oslo, Norway; 365 National Cancer Institute, National Institutes of Health, Bethesda, MD USA; 366 Royal Marsden NHS Foundation Trust, London and Sutton, UK; 367 Genome Biology Unit, European Molecular Biology Laboratory (EMBL), Heidelberg, Germany; 368 Department of Oncology, University of Cambridge, Cambridge, UK; 369 Li Ka Shing Centre, Cancer Research UK Cambridge Institute, University of Cambridge, Cambridge, UK; 370 Institut Gustave Roussy, Villejuif, France; 371 Cambridge University Hospitals NHS Foundation Trust, Cambridge, UK; 372 Department of Haematology, University of Cambridge, Cambridge, UK; 373 Anatomia Patológica, Hospital Clinic, Institut d’Investigacions Biomèdiques August Pi i Sunyer (IDIBAPS), University of Barcelona, Barcelona, Spain; 374 Spanish Ministry of Science and Innovation, Madrid, Spain; 375 University of Michigan Comprehensive Cancer Center, Ann Arbor, MI USA; 376 Department for BioMedical Research, University of Bern, Bern, Switzerland; 377 Department of Medical Oncology, Inselspital, University Hospital and University of Bern, Bern, Switzerland; 378 Graduate School for Cellular and Biomedical Sciences, University of Bern, Bern, Switzerland; 379 University of Pavia, Pavia, Italy; 380 University of Alabama at Birmingham, Birmingham, AL USA; 381 UHN Program in BioSpecimen Sciences, Toronto General Hospital, Toronto, ON Canada; 382 Department of Urology, Icahn School of Medicine at Mount Sinai, New York, NY USA; 383 Centre for Law and Genetics, University of Tasmania, Sandy Bay Campus, Hobart, TAS Australia; 384 Faculty of Biosciences, Heidelberg University, Heidelberg, Germany; 385 Department of Biochemistry, Microbiology and Immunology, Faculty of Medicine, University of Ottawa, Ottawa, ON Canada; 386 Division of Anatomic Pathology, Mayo Clinic, Rochester, MN USA; 387 Division of Cancer Epidemiology and Genetics, National Cancer Institute, National Institutes of Health, Bethesda, MD USA; 388 Illawarra Shoalhaven Local Health District L3 Illawarra Cancer Care Centre, Wollongong Hospital, Wollongong, NSW Australia; 389 BioForA, French National Institute for Agriculture, Food, and Environment (INRAE), ONF, Orléans, France; 390 Department of Biostatistics, Bloomberg School of Public Health, Johns Hopkins University, Baltimore, MD USA; 391 University of California San Diego, San Diego, CA USA; 392 Division of Experimental Pathology, Mayo Clinic, Rochester, MN USA; 393 Centre for Cancer Research, The Westmead Institute for Medical Research, University of Sydney, Sydney, NSW Australia; 394 Department of Gynaecological Oncology, Westmead Hospital, Sydney, NSW Australia; 395 PDXen Biosystems Inc, Seoul, South Korea; 396 Korea Advanced Institute of Science and Technology, Daejeon, South Korea; 397 Electronics and Telecommunications Research Institute, Daejeon, South Korea; 398 Institut National du Cancer (INCA), Boulogne-Billancourt, France; 399 Department of Genetics, Informatics Institute, University of Alabama at Birmingham, Birmingham, AL USA; 400 Division of Medical Oncology, National Cancer Centre, Singapore, Singapore; 401 Medical Oncology, University and Hospital Trust of Verona, Verona, Italy; 402 Department of Pediatrics, University Hospital Schleswig-Holstein, Kiel, Germany; 403 Hepatobiliary/Pancreatic Surgical Oncology Program, University Health Network, Toronto, ON Canada; 404 School of Biological Sciences, University of Auckland, Auckland, New Zealand; 405 Department of Surgery, University of Melbourne, Parkville, VIC Australia; 406 The Murdoch Children’s Research Institute, Royal Children’s Hospital, Parkville, VIC Australia; 407 Walter and Eliza Hall Institute, Parkville, VIC Australia; 408 Vancouver Prostate Centre, Vancouver, Canada; 409 Lunenfeld-Tanenbaum Research Institute, Mount Sinai Hospital, Toronto, ON Canada; 410 University of East Anglia, Norwich, UK; 411 Norfolk and Norwich University Hospital NHS Trust, Norwich, UK; 412 Victorian Institute of Forensic Medicine, Southbank, VIC Australia; 413 Department of Biomedical Informatics, Harvard Medical School, Boston, MA USA; 414 Department of Chemistry, Centre for Molecular Science Informatics, University of Cambridge, Cambridge, UK; 415 Ludwig Center at Harvard Medical School, Boston, MA USA; 416 Human Genome Sequencing Center, Baylor College of Medicine, Houston, TX USA; 417 Peter MacCallum Cancer Centre, University of Melbourne, Melbourne, VIC Australia; 418 Physics Division, Optimization and Systems Biology Lab, Massachusetts General Hospital, Boston, MA USA; 419 Department of Medicine, Baylor College of Medicine, Houston, TX USA; 420 University of Cologne, Cologne, Germany; 421 International Genomics Consortium, Phoenix, AZ USA; 422 Genomics Research Program, Ontario Institute for Cancer Research, Toronto, ON Canada; 423 Barking Havering and Redbridge University Hospitals NHS Trust, Romford, UK; 424 Children’s Hospital at Westmead, University of Sydney, Sydney, NSW Australia; 425 Department of Medicine, Section of Endocrinology, University and Hospital Trust of Verona, Verona, Italy; 426 Computational Biology Center, Memorial Sloan Kettering Cancer Center, New York, NY USA; 427 Department of Biology, ETH Zurich, Zürich, Switzerland; 428 Department of Computer Science, ETH Zurich, Zurich, Switzerland; 429 SIB Swiss Institute of Bioinformatics, Lausanne, Switzerland; 430 Weill Cornell Medical College, New York, NY USA; 431 Academic Department of Medical Genetics, University of Cambridge, Addenbrooke’s Hospital, Cambridge, UK; 432 MRC Cancer Unit, University of Cambridge, Cambridge, UK; 433 Departments of Pediatrics and Genetics, University of North Carolina at Chapel Hill, Chapel Hill, NC USA; 434 Seven Bridges Genomics, Charlestown, MA USA; 435 Annai Systems, Inc, Carlsbad, CA USA; 436 Department of Pathology, General Hospital of Treviso, Department of Medicine, University of Padua, Treviso, Italy; 437 Department of Computational Biology, University of Lausanne, Lausanne, Switzerland; 438 Department of Genetic Medicine and Development, University of Geneva Medical School, Geneva, CH Switzerland; 439 Swiss Institute of Bioinformatics, University of Geneva, Geneva, CH Switzerland; 440 The Francis Crick Institute, London, UK; 441 University of Leuven, Leuven, Belgium; 442 Institute of Medical Genetics and Applied Genomics, University of Tübingen, Tübingen, Germany; 443 Computational and Systems Biology, Genome Institute of Singapore, Singapore, Singapore; 444 School of Computing, National University of Singapore, Singapore, Singapore; 445 Big Data Institute, Li Ka Shing Centre, University of Oxford, Oxford, UK; 446 Biomedical Data Science Laboratory, Francis Crick Institute, London, UK; 447 Bioinformatics Group, Department of Computer Science, University College London, London, UK; 448 The Edward S. Rogers Sr. Department of Electrical and Computer Engineering, University of Toronto, Toronto, ON Canada; 449 Breast Cancer Translational Research Laboratory JC Heuson, Institut Jules Bordet, Brussels, Belgium; 450 Department of Oncology, Laboratory for Translational Breast Cancer Research, KU Leuven, Leuven, Belgium; 451 Institute for Research in Biomedicine (IRB Barcelona), The Barcelona Institute of Science and Technology, Barcelona, Spain; 452 Research Program on Biomedical Informatics, Universitat Pompeu Fabra, Barcelona, Spain; 453 Division of Medical Oncology, Princess Margaret Cancer Centre, Toronto, ON Canada; 454 Department of Physiology and Biophysics, Weill Cornell Medicine, New York, NY USA; 455 Institute for Computational Biomedicine, Weill Cornell Medicine, New York, NY USA; 456 Department of Pathology, UPMC Shadyside, Pittsburgh, PA USA; 457 Independent Consultant, Wellesley, USA; 458 Department of Cell and Molecular Biology, Science for Life Laboratory, Uppsala University, Uppsala, Sweden; 459 Department of Medicine and Department of Genetics, Washington University School of Medicine, St. Louis, St. Louis, MO USA; 460 Hefei University of Technology, Anhui, China; 461 Translational Cancer Research Unit, GZA Hospitals St.-Augustinus, Center for Oncological Research, Faculty of Medicine and Health Sciences, University of Antwerp, Antwerp, Belgium; 462 Simon Fraser University, Burnaby, BC Canada; 463 University of Pennsylvania, Philadelphia, PA USA; 464 Faculty of Science and Technology, University of Vic—Central University of Catalonia (UVic-UCC), Vic, Spain; 465 The Wellcome Trust, London, UK; 466 The Hospital for Sick Children, Toronto, ON Canada; 467 Department of Pathology, Queen Elizabeth University Hospital, Glasgow, UK; 468 Department of Genetics and Computational Biology, QIMR Berghofer Medical Research Institute, Brisbane, QLD Australia; 469 Department of Oncology, Centre for Cancer Genetic Epidemiology, University of Cambridge, Cambridge, UK; 470 Department of Public Health and Primary Care, Centre for Cancer Genetic Epidemiology, University of Cambridge, Cambridge, UK; 471 Prostate Cancer Canada, Toronto, ON Canada; 472 University of Cambridge, Cambridge, UK; 473 Department of Laboratory Medicine, Translational Cancer Research, Lund University Cancer Center at Medicon Village, Lund University, Lund, Sweden; 474 Heidelberg University, Heidelberg, Germany; 475 New BIH Digital Health Center, Berlin Institute of Health (BIH) and Charité - Universitätsmedizin Berlin, Berlin, Germany; 476 CIBER Epidemiología y Salud Pública (CIBERESP), Madrid, Spain; 477 Research Group on Statistics, Econometrics and Health (GRECS), UdG, Barcelona, Spain; 478 Quantitative Genomics Laboratories (qGenomics), Barcelona, Spain; 479 Icelandic Cancer Registry, Icelandic Cancer Society, Reykjavik, Iceland; 480 State Key Laboratory of Cancer Biology, and Xijing Hospital of Digestive Diseases, Fourth Military Medical University, Shaanxi, China; 481 Department of Medicine (DIMED), Surgical Pathology Unit, University of Padua, Padua, Italy; 482 Rigshospitalet, Copenhagen, Denmark; 483 Center for Cancer Genomics, National Cancer Institute, National Institutes of Health, Bethesda, MD USA; 484 Department of Biochemistry and Molecular Medicine, University of Montreal, Montreal, QC Canada; 485 Australian Institute of Tropical Health and Medicine, James Cook University, Douglas, QLD Australia; 486 Department of Neuro-Oncology, Istituto Neurologico Besta, Milano, Italy; 487 Bioplatforms Australia, North Ryde, NSW Australia; 488 Department of Pathology (Research), University College London Cancer Institute, London, UK; 489 Department of Surgical Oncology, Princess Margaret Cancer Centre, Toronto, ON Canada; 490 Department of Medical Oncology, Josephine Nefkens Institute and Cancer Genomics Centre, Erasmus Medical Center, Rotterdam, CN The Netherlands; 491 The University of Queensland Thoracic Research Centre, The Prince Charles Hospital, Brisbane, QLD Australia; 492 CIBIO/InBIO - Research Center in Biodiversity and Genetic Resources, Universidade do Porto, Vairão, Portugal; 493 HCA Laboratories, London, UK; 494 University of Liverpool, Liverpool, UK; 495 The Azrieli Faculty of Medicine, Bar-Ilan University, Safed, Israel; 496 Department of Neurosurgery, University of Florida, Gainesville, FL USA; 497 Department of Pathology, Graduate School of Medicine, University of Tokyo, Tokyo, Japan; 498 University of Milano Bicocca, Monza, Italy; 499 BGI-Shenzhen, Shenzhen, China; 500 Department of Pathology, Oslo University Hospital Ulleval, Oslo, Norway; 501 Center for Biomedical Informatics, Harvard Medical School, Boston, MA USA; 502 Department Biochemistry and Molecular Biomedicine, University of Barcelona, Barcelona, Spain; 503 Office of Cancer Genomics, National Cancer Institute, National Institutes of Health, Bethesda, MD USA; 504 Cancer Epigenomics, German Cancer Research Center (DKFZ), Heidelberg, Germany; 505 Department of Cancer Biology, The University of Texas MD Anderson Cancer Center, Houston, TX USA; 506 Department of Surgical Oncology, The University of Texas MD Anderson Cancer Center, Houston, TX USA; 507 Department of Computer Science, Yale University, New Haven, CT USA; 508 Department of Molecular Biophysics and Biochemistry, Yale University, New Haven, CT USA; 509 Program in Computational Biology and Bioinformatics, Yale University, New Haven, CT USA; 510 Center for Cancer Research, Massachusetts General Hospital, Boston, MA USA; 511 Department of Pathology, Massachusetts General Hospital, Boston, MA USA; 512 Department of Pathology, Memorial Sloan Kettering Cancer Center, New York, NY USA; 513 Division of Gastroenterology and Hepatology, Mayo Clinic, Rochester, MN USA; 514 University of Sydney, Sydney, NSW Australia; 515 University of Oxford, Oxford, UK; 516 Department of Surgery, Academic Urology Group, University of Cambridge, Cambridge, UK; 517 Department of Medicine II, University of Würzburg, Wuerzburg, Germany; 518 Sylvester Comprehensive Cancer Center, University of Miami, Miami, FL USA; 519 Institut Hospital del Mar d’Investigacions Mèdiques (IMIM), Barcelona, Spain; 520 Genome Integrity and Structural Biology Laboratory, National Institute of Environmental Health Sciences (NIEHS), Durham, NC USA; 521 St. Thomas’s Hospital, London, UK; 522 Osaka International Cancer Center, Osaka, Japan; 523 Department of Pathology, Skåne University Hospital, Lund University, Lund, Sweden; 524 Department of Medical Oncology, Beatson West of Scotland Cancer Centre, Glasgow, UK; 525 National Human Genome Research Institute, National Institutes of Health, Bethesda, MD USA; 526 Centre for Cancer Research, Victorian Comprehensive Cancer Centre, University of Melbourne, Melbourne, VIC Australia; 527 Department of Medicine, Section of Hematology/Oncology, University of Chicago, Chicago, IL USA; 528 German Center for Infection Research (DZIF), Partner Site Hamburg-Borstel-Lübeck-Riems, Hamburg, Germany; 529 Bioinformatics Research Centre (BiRC), Aarhus University, Aarhus, Denmark; 530 Department of Biotechnology, Ministry of Science and Technology, Government of India, New Delhi, Delhi India; 531 National Cancer Centre Singapore, Singapore, Singapore; 532 Brandeis University, Waltham, MA USA; 533 Department of Urologic Sciences, University of British Columbia, Vancouver, BC Canada; 534 Department of Internal Medicine, Stanford University, Stanford, CA USA; 535 The University of Texas Health Science Center at Houston, Houston, TX USA; 536 Imperial College NHS Trust, Imperial College, London, INY UK; 537 Senckenberg Institute of Pathology, University of Frankfurt Medical School, Frankfurt, Germany; 538 Department of Medicine, Division of Biomedical Informatics, UC San Diego School of Medicine, San Diego, CA USA; 539 Center for Precision Health, School of Biomedical Informatics, The University of Texas Health Science Center, Houston, TX USA; 540 Oxford Nanopore Technologies, New York, NY USA; 541 Institute of Medical Science, University of Tokyo, Tokyo, Japan; 542 Howard Hughes Medical Institute, University of California Santa Cruz, Santa Cruz, CA USA; 543 Wakayama Medical University, Wakayama, Japan; 544 Department of Internal Medicine, Division of Medical Oncology, Lineberger Comprehensive Cancer Center, University of North Carolina at Chapel Hill, Chapel Hill, NC USA; 545 University of Tennessee Health Science Center for Cancer Research, Memphis, TN USA; 546 Department of Histopathology, Salford Royal NHS Foundation Trust, Salford, UK; 547 Faculty of Biology, Medicine and Health, University of Manchester, Manchester, UK; 548 BIOPIC, ICG and College of Life Sciences, Peking University, Beijing, China; 549 Peking-Tsinghua Center for Life Sciences, Peking University, Beijing, China; 550 Children’s Hospital of Philadelphia, Philadelphia, PA USA; 551 Department of Bioinformatics and Computational Biology and Department of Systems Biology, The University of Texas MD Anderson Cancer Center, Houston, TX USA; 552 Karolinska Institute, Stockholm, Sweden; 553 The Donnelly Centre, University of Toronto, Toronto, ON Canada; 554 Department of Medical Genetics, College of Medicine, Hallym University, Chuncheon, South Korea; 555 Department of Experimental and Health Sciences, Institute of Evolutionary Biology (UPF-CSIC), Universitat Pompeu Fabra, Barcelona, Spain; 556 Health Data Science Unit, University Clinics, Heidelberg, Germany; 557 Massachusetts General Hospital Center for Cancer Research, Charlestown, MA USA; 558 Hokkaido University, Sapporo, Japan; 559 Department of Pathology and Clinical Laboratory, National Cancer Center Hospital, Tokyo, Japan; 560 Department of Genetics, University of North Carolina at Chapel Hill, Chapel Hill, NC USA; 561 Computational Biology, Leibniz Institute on Aging - Fritz Lipmann Institute (FLI), Jena, Germany; 562 University of Melbourne Centre for Cancer Research, Melbourne, VIC Australia; 563 University of Nebraska Medical Center, Omaha, NE USA; 564 Syntekabio Inc, Daejeon, South Korea; 565 Department of Pathology, Academic Medical Center, Amsterdam, AZ The Netherlands; 566 China National GeneBank-Shenzhen, Shenzhen, China; 567 Division of Molecular Genetics, German Cancer Research Center (DKFZ), Heidelberg, Germany; 568 Division of Life Science and Applied Genomics Center, Hong Kong University of Science and Technology, Clear Water Bay, Hong Kong, China; 569 Icahn School of Medicine at Mount Sinai, New York, NY USA; 570 Geneplus-Shenzhen, Shenzhen, China; 571 School of Computer Science and Technology, Xi’an Jiaotong University, Xi’an, China; 572 AbbVie, North Chicago, IL USA; 573 Institute of Pathology, Charité – University Medicine Berlin, Berlin, Germany; 574 Centre for Translational and Applied Genomics, British Columbia Cancer Agency, Vancouver, BC Canada; 575 Edinburgh Royal Infirmary, Edinburgh, UK; 576 Berlin Institute for Medical Systems Biology, Max Delbrück Center for Molecular Medicine, Berlin, Germany; 577 Department of Pediatric Immunology, Hematology and Oncology, University Hospital, Heidelberg, Germany; 578 German Cancer Research Center (DKFZ), Heidelberg, Germany; 579 Heidelberg Institute for Stem Cell Technology and Experimental Medicine (HI-STEM), Heidelberg, Germany; 580 Institute for Computational Biomedicine, Weill Cornell Medical College, New York, NY USA; 581 New York Genome Center, New York, NY USA; 582 Department of Urology, James Buchanan Brady Urological Institute, Johns Hopkins University School of Medicine, Baltimore, MD USA; 583 Department of Preventive Medicine, Graduate School of Medicine, The University of Tokyo, Tokyo, Japan; 584 Department of Molecular and Cellular Biology, Baylor College of Medicine, Houston, TX USA; 585 Department of Pathology and Immunology, Baylor College of Medicine, Houston, TX USA; 586 Michael E. DeBakey Veterans Affairs Medical Center, Houston, TX USA; 587 Technical University of Denmark, Lyngby, Denmark; 588 Department of Pathology, College of Medicine, Hanyang University, Seoul, South Korea; 589 Academic Unit of Surgery, School of Medicine, College of Medical, Veterinary and Life Sciences, University of Glasgow, Glasgow Royal Infirmary, Glasgow, UK; 590 Department of Pathology, Asan Medical Center, College of Medicine, Ulsan University, Songpa-gu, Seoul South Korea; 591 Science Writer, Garrett Park, MD USA; 592 International Cancer Genome Consortium (ICGC)/ICGC Accelerating Research in Genomic Oncology (ARGO) Secretariat, Ontario Institute for Cancer Research, Toronto, ON Canada; 593 University of Ljubljana, Ljubljana, Slovenia; 594 Department of Public Health Sciences, University of Chicago, Chicago, IL USA; 595 Research Institute, NorthShore University HealthSystem, Evanston, IL USA; 596 Department for Biomedical Research, University of Bern, Bern, Switzerland; 597 Centre of Genomics and Policy, McGill University and Génome Québec Innovation Centre, Montreal, QC Canada; 598 Carolina Center for Genome Sciences, University of North Carolina at Chapel Hill, Chapel Hill, NC USA; 599 Hopp Children’s Cancer Center (KiTZ), Heidelberg, Germany; 600 Pediatric Glioma Research Group, German Cancer Research Center (DKFZ), Heidelberg, Germany; 601 Cancer Research UK, London, UK; 602 Indivumed GmbH, Hamburg, Germany; 603 Genome Integration Data Center, Syntekabio, Inc, Daejeon, South Korea; 604 University Hospital Zurich, Zurich, Switzerland; 605 Clinical Bioinformatics, Swiss Institute of Bioinformatics, Geneva, Switzerland; 606 Institute for Pathology and Molecular Pathology, University Hospital Zurich, Zurich, Switzerland; 607 Institute of Molecular Life Sciences, University of Zurich, Zurich, Switzerland; 608 MRC Human Genetics Unit, MRC IGMM, University of Edinburgh, Edinburgh, UK; 609 Women’s Cancer Program at the Samuel Oschin Comprehensive Cancer Institute, Cedars-Sinai Medical Center, Los Angeles, CA USA; 610 Department of Biology, Bioinformatics Group, Division of Molecular Biology, Faculty of Science, University of Zagreb, Zagreb, Croatia; 611 Department for Internal Medicine II, University Hospital Schleswig-Holstein, Kiel, Germany; 612 Genetics and Molecular Pathology, SA Pathology, Adelaide, SA Australia; 613 Department of Gastric Surgery, National Cancer Center Hospital, Tokyo, Japan; 614 Department of Bioinformatics, Division of Cancer Genomics, National Cancer Center Research Institute, Tokyo, Japan; 615 A.A. Kharkevich Institute of Information Transmission Problems, Moscow, Russia; 616 Oncology and Immunology, Dmitry Rogachev National Research Center of Pediatric Hematology, Moscow, Russia; 617 Skolkovo Institute of Science and Technology, Moscow, Russia; 618 Department of Surgery, The George Washington University, School of Medicine and Health Science, Washington, DC USA; 619 Endocrine Oncology Branch, Center for Cancer Research, National Cancer Institute, National Institutes of Health, Bethesda, MD USA; 620 Melanoma Institute Australia, Macquarie University, Sydney, NSW Australia; 621 MIT Computer Science and Artificial Intelligence Laboratory, Massachusetts Institute of Technology, Cambridge, MA USA; 622 Tissue Pathology and Diagnostic Oncology, Royal Prince Alfred Hospital, Sydney, NSW Australia; 623 Cholangiocarcinoma Screening and Care Program and Liver Fluke and Cholangiocarcinoma Research Centre, Faculty of Medicine, Khon Kaen University, Khon Kaen, Thailand; 624 Controlled Department and Institution, New York, NY USA; 625 Englander Institute for Precision Medicine, Weill Cornell Medicine, New York, NY USA; 626 National Cancer Center, Gyeonggi, South Korea; 627 Department of Biochemistry, College of Medicine, Ewha Womans University, Seoul, South Korea; 628 Health Sciences Department of Biomedical Informatics, University of California San Diego, La Jolla, CA USA; 629 Research Core Center, National Cancer Centre Korea, Goyang-si, South Korea; 630 Department of Health Sciences and Technology, Sungkyunkwan University School of Medicine, Seoul, South Korea; 631 Samsung Genome Institute, Seoul, South Korea; 632 Breast Oncology Program, Dana-Farber/Brigham and Women’s Cancer Center, Boston, MA USA; 633 Department of Surgery, Memorial Sloan Kettering Cancer Center, New York, NY USA; 634 Division of Breast Surgery, Brigham and Women’s Hospital, Boston, MA USA; 635 Integrative Bioinformatics Support Group, National Institute of Environmental Health Sciences (NIEHS), Durham, NC USA; 636 Department of Clinical Science, University of Bergen, Bergen, Norway; 637 Center For Medical Innovation, Seoul National University Hospital, Seoul, South Korea; 638 Department of Internal Medicine, Seoul National University Hospital, Seoul, South Korea; 639 Institute of Computer Science, Polish Academy of Sciences, Warsawa, Poland; 640 Functional and Structural Genomics, German Cancer Research Center (DKFZ), Heidelberg, Germany; 641 Laboratory of Translational Genomics, Division of Cancer Epidemiology and Genetics, National Cancer Institute, , National Institutes of Health, Bethesda, MD USA; 642 Institute for Medical Informatics Statistics and Epidemiology, University of Leipzig, Leipzig, Germany; 643 Morgan Welch Inflammatory Breast Cancer Research Program and Clinic, The University of Texas MD Anderson Cancer Center, Houston, TX USA; 644 Department of Hematology and Oncology, Georg-Augusts-University of Göttingen, Göttingen, Germany; 645 Institute of Cell Biology (Cancer Research), University of Duisburg-Essen, Essen, Germany; 646 King’s College London and Guy’s and St. Thomas’ NHS Foundation Trust, London, UK; 647 Center for Epigenetics, Van Andel Research Institute, Grand Rapids, MI USA; 648 The University of Queensland Centre for Clinical Research, Royal Brisbane and Women’s Hospital, Herston, QLD Australia; 649 Department of Pediatric Oncology and Hematology, University of Cologne, Cologne, Germany; 650 University of Düsseldorf, Düsseldorf, Germany; 651 Department of Pathology, Institut Jules Bordet, Brussels, Belgium; 652 Institute of Biomedicine, Sahlgrenska Academy at University of Gothenburg, Gothenburg, Sweden; 653 Children’s Medical Research Institute, Sydney, NSW Australia; 654 ILSbio, LLC Biobank, Chestertown, MD USA; 655 Division of Genetics and Genomics, Boston Children’s Hospital, Harvard Medical School, Boston, MA USA; 656 Institute for Bioengineering and Biopharmaceutical Research (IBBR), Hanyang University, Seoul, South Korea; 657 Department of Statistics, University of California Santa Cruz, Santa Cruz, CA USA; 658 National Genotyping Center, Institute of Biomedical Sciences, Academia Sinica, Taipei, Taiwan; 659 Department of Vertebrate Genomics/Otto Warburg Laboratory Gene Regulation and Systems Biology of Cancer, Max Planck Institute for Molecular Genetics, Berlin, Germany; 660 McGill University and Genome Quebec Innovation Centre, Montreal, QC Canada; 661 biobyte solutions GmbH, Heidelberg, Germany; 662 Gynecologic Oncology, NYU Laura and Isaac Perlmutter Cancer Center, New York University, New York, NY USA; 663 Division of Oncology, Stem Cell Biology Section, Washington University School of Medicine, St. Louis, MO USA; 664 Department of Systems Biology, The University of Texas MD Anderson Cancer Center, Houston, TX USA; 665 Harvard University, Cambridge, MA USA; 666 Urologic Oncology Branch, Center for Cancer Research, National Cancer Institute, National Institutes of Health, Bethesda, MD USA; 667 University of Oslo, Oslo, Norway; 668 University of Toronto, Toronto, ON Canada; 669 Peking University, Beijing, China; 670 School of Life Sciences, Peking University, Beijing, China; 671 Leidos Biomedical Research, Inc, McLean, VA USA; 672 Hematology, Hospital Clinic, Institut d’Investigacions Biomèdiques August Pi i Sunyer (IDIBAPS), University of Barcelona, Barcelona, Spain; 673 Second Military Medical University, Shanghai, China; 674 Chinese Cancer Genome Consortium, Shenzhen, China; 675 Department of Medical Oncology, Beijing Hospital, Beijing, China; 676 Laboratory of Molecular Oncology, Key Laboratory of Carcinogenesis and Translational Research (Ministry of Education), Peking University Cancer Hospital and Institute, Beijing, China; 677 School of Medicine/School of Mathematics and Statistics, University of St. Andrews, St, Andrews, Fife UK; 678 Institute for Systems Biology, Seattle, WA USA; 679 Department of Biochemistry and Molecular Biology, Faculty of Medicine, University Institute of Oncology-IUOPA, Oviedo, Spain; 680 Institut Bergonié, Bordeaux, France; 681 Cancer Unit, MRC University of Cambridge, Cambridge, UK; 682 Department of Pathology and Laboratory Medicine, Center for Personalized Medicine, Children’s Hospital Los Angeles, Los Angeles, CA USA; 683 John Curtin School of Medical Research, Canberra, ACT Australia; 684 MVZ Department of Oncology, PraxisClinic am Johannisplatz, Leipzig, Germany; 685 Department of Information Technology, Ghent University, Ghent, Belgium; 686 Department of Plant Biotechnology and Bioinformatics, Ghent University, Ghent, Belgium; 687 Institute for Genomic Medicine, Nationwide Children’s Hospital, Columbus, OH USA; 688 Computational Biology Program, School of Medicine, Oregon Health and Science University, Portland, OR USA; 689 Department of Surgery, Duke University, Durham, NC USA; 690 Institució Catalana de Recerca i Estudis Avançats (ICREA), Barcelona, Spain; 691 Institut Català de Paleontologia Miquel Crusafont, Universitat Autònoma de Barcelona, Barcelona, Spain; 692 University of Glasgow, Glasgow, UK; 693 Institut d’Investigacions Biomèdiques August Pi i Sunyer (IDIBAPS), Barcelona, Spain; 694 Division of Oncology, Washington University School of Medicine, St. Louis, MO USA; 695 Department of Surgery and Cancer, Imperial College, London, INY UK; 696 Applications Department, Oxford Nanopore Technologies, Oxford, UK; 697 Department of Obstetrics, Gynecology and Reproductive Services, University of California San Francisco, San Francisco, CA USA; 698 Department of Biochemistry and Molecular Medicine, University California at Davis, Sacramento, CA USA; 699 STTARR Innovation Facility, Princess Margaret Cancer Centre, Toronto, ON Canada; 700 Discipline of Surgery, Western Sydney University, Penrith, NSW Australia; 701 Yale School of Medicine, Yale University, New Haven, CT USA; 702 Department of Genetics, Lineberger Comprehensive Cancer Center, University of North Carolina at Chapel Hill, Chapel Hill, NC USA; 703 Departments of Neurology and Neurosurgery, Henry Ford Hospital, Detroit, MI USA; 704 Precision Oncology, OHSU Knight Cancer Institute, Oregon Health and Science University, Portland, OR USA; 705 Institute of Pathology, University Medical Center Hamburg-Eppendorf, Hamburg, Germany; 706 Department of Health Sciences, Faculty of Medical Sciences, Kyushu University, Fukuoka, Japan; 707 Heidelberg Academy of Sciences and Humanities, Heidelberg, Germany; 708 Department of Clinical Pathology, University of Melbourne, Melbourne, VIC, Australia; 709 Department of Pathology, Roswell Park Cancer Institute, Buffalo, NY USA; 710 Department of Computer Science, University of Helsinki, Helsinki, Finland; 711 Institute of Biotechnology, University of Helsinki, Helsinki, Finland; 712 Organismal and Evolutionary Biology Research Programme, University of Helsinki, Helsinki, Finland; 713 Department of Obstetrics and Gynecology, Division of Gynecologic Oncology, Washington University School of Medicine, St. Louis, MO USA; 714 Penrose St. Francis Health Services, Colorado Springs, CO USA; 715 Institute of Pathology, Ulm University and University Hospital of Ulm, Ulm, Germany; 716 National Cancer Center, Tokyo, Japan; 717 Genome Institute of Singapore, Singapore, Singapore; 718 32Program in Computational Biology and Bioinformatics, Yale University, New Haven, CT USA; 719 German Cancer Aid, Bonn, Germany; 720 Programme in Cancer and Stem Cell Biology, Centre for Computational Biology, Duke-NUS Medical School, Singapore, Singapore; 721 The Chinese University of Hong Kong, Shatin, NT, Hong Kong China; 722 Fourth Military Medical University, Shaanxi, China; 723 The University of Cambridge School of Clinical Medicine, Cambridge, UK; 724 St. Jude Children’s Research Hospital, Memphis, TN USA; 725 University Health Network, Princess Margaret Cancer Centre, Toronto, ON Canada; 726 Center for Biomolecular Science and Engineering, University of California Santa Cruz, Santa Cruz, CA USA; 727 Department of Medicine, University of Chicago, Chicago, IL USA; 728 Department of Neurology, Mayo Clinic, Rochester, MN USA; 729 Cambridge Oesophagogastric Centre, Cambridge University Hospitals NHS Foundation Trust, Cambridge, UK; 730 Department of Computer Science, Carleton College, Northfield, MN USA; 731 Institute of Cancer Sciences, College of Medical Veterinary and Life Sciences, University of Glasgow, Glasgow, UK; 732 Department of Epidemiology, University of Alabama at Birmingham, Birmingham, AL USA; 733 HudsonAlpha Institute for Biotechnology, Huntsville, AL USA; 734 O’Neal Comprehensive Cancer Center, University of Alabama at Birmingham, Birmingham, AL USA; 735 Department of Pathology, Keio University School of Medicine, Tokyo, Japan; 736 Department of Hepatobiliary and Pancreatic Oncology, National Cancer Center Hospital, Tokyo, Japan; 737 Sage Bionetworks, Seattle, WA USA; 738 Lymphoma Genomic Translational Research Laboratory, National Cancer Centre, Singapore, Singapore; 739 Department of Clinical Pathology, Robert-Bosch-Hospital, Stuttgart, Germany; 740 Department of Cell and Systems Biology, University of Toronto, Toronto, ON Canada; 741 Department of Biosciences and Nutrition, Karolinska Institutet, Stockholm, Sweden; 742 Center for Liver Cancer, Research Institute and Hospital, National Cancer Center, Gyeonggi, South Korea; 743 Division of Hematology-Oncology, Samsung Medical Center, Sungkyunkwan University School of Medicine, Seoul, South Korea; 744 Samsung Advanced Institute for Health Sciences and Technology, Sungkyunkwan University School of Medicine, Seoul, South Korea; 745 Cheonan Industry-Academic Collaboration Foundation, Sangmyung University, Cheonan, South Korea; 746 NYU Langone Medical Center, New York, NY USA; 747 Department of Hematology and Medical Oncology, Cleveland Clinic, Cleveland, OH USA; 748 Department of Radiation Oncology, University of California San Francisco, San Francisco, CA USA; 749 Department of Health Sciences Research, Mayo Clinic, Rochester, MN USA; 750 Helen F. Graham Cancer Center at Christiana Care Health Systems, Newark, DE USA; 751 Heidelberg University Hospital, Heidelberg, Germany; 752 CSRA Incorporated, Fairfax, VA USA; 753 Research Department of Pathology, University College London Cancer Institute, London, UK; 754 Department of Research Oncology, Guy’s Hospital, King’s Health Partners AHSC, King’s College London School of Medicine, London, UK; 755 Faculty of Medicine and Health Sciences, Macquarie University, Sydney, NSW Australia; 756 University Hospital of Minjoz, INSERM UMR 1098, Besançon, France; 757 Spanish National Cancer Research Centre, Madrid, Spain; 758 Center of Digestive Diseases and Liver Transplantation, Fundeni Clinical Institute, Bucharest, Romania; 759 Cureline, Inc, South San Francisco, CA USA; 760 St. Luke’s Cancer Centre, Royal Surrey County Hospital NHS Foundation Trust, Guildford, UK; 761 Cambridge Breast Unit, Addenbrooke’s Hospital, Cambridge University Hospital NHS Foundation Trust and NIHR Cambridge Biomedical Research Centre, Cambridge, UK; 762 East of Scotland Breast Service, Ninewells Hospital, Aberdeen, UK; 763 Department of Genetics, Microbiology and Statistics, University of Barcelona, IRSJD, IBUB, Barcelona, Spain; 764 Department of Obstetrics and Gynecology, Medical College of Wisconsin, Milwaukee, WI USA; 765 Hematology and Medical Oncology, Winship Cancer Institute of Emory University, Atlanta, GA USA; 766 Department of Computer Science, Princeton University, Princeton, NJ USA; 767 Vanderbilt Ingram Cancer Center, Vanderbilt University, Nashville, TN USA; 768 Ohio State University College of Medicine and Arthur G. James Comprehensive Cancer Center, Columbus, OH USA; 769 Department of Surgery, Yokohama City University Graduate School of Medicine, Kanagawa, Japan; 770 Division of Chromatin Networks, German Cancer Research Center (DKFZ) and BioQuant, Heidelberg, Germany; 771 Research Computing Center, University of North Carolina at Chapel Hill, Chapel Hill, NC USA; 772 School of Molecular Biosciences and Center for Reproductive Biology, Washington State University, Pullman, WA USA; 773 Finsen Laboratory and Biotech Research and Innovation Centre (BRIC), University of Copenhagen, Copenhagen, Denmark; 774 Department of Laboratory Medicine and Pathobiology, University of Toronto, Toronto, ON Canada; 775 Department of Pathology, Human Oncology and Pathogenesis Program, Memorial Sloan Kettering Cancer Center, New York, NY USA; 776 University Hospital Giessen, Pediatric Hematology and Oncology, Giessen, Germany; 777 Oncologie Sénologie, ICM Institut Régional du Cancer, Montpellier, France; 778 Institute of Clinical Molecular Biology, Christian-Albrechts-University, Kiel, Germany; 779 Institute of Pathology, University of Wuerzburg, Wuerzburg, Germany; 780 Department of Urology, North Bristol NHS Trust, Bristol, UK; 781 SingHealth, Duke-NUS Institute of Precision Medicine, National Heart Centre Singapore, Singapore, Singapore; 782 Department of Computer Science, University of Toronto, Toronto, ON Canada; 783 Bern Center for Precision Medicine, University Hospital of Bern, University of Bern, Bern, Switzerland; 784 Englander Institute for Precision Medicine, Weill Cornell Medicine and New York Presbyterian Hospital, New York, NY USA; 785 Meyer Cancer Center, Weill Cornell Medicine, New York, NY USA; 786 Pathology and Laboratory, Weill Cornell Medical College, New York, NY USA; 787 Vall d’Hebron Institute of Oncology: VHIO, Barcelona, Spain; 788 General and Hepatobiliary-Biliary Surgery, Pancreas Institute, University and Hospital Trust of Verona, Verona, Italy; 789 National Centre for Biological Sciences, Tata Institute of Fundamental Research, Bangalore, India; 790 Indiana University, Bloomington, IN USA; 791 Department of Pathology, GZA-ZNA Hospitals, Antwerp, Belgium; 792 Analytical Biological Services, Inc, Wilmington, DE USA; 793 Sydney Medical School, University of Sydney, Sydney, NSW Australia; 794 cBio Center, Dana-Farber Cancer Institute, Harvard Medical School, Boston, MA USA; 795 Department of Cell Biology, Harvard Medical School, Boston, MA USA; 796 Advanced Centre for Treatment Research and Education in Cancer, Tata Memorial Centre, Navi Mumbai, Maharashtra India; 797 School of Environmental and Life Sciences, Faculty of Science, The University of Newcastle, Ourimbah, NSW Australia; 798 Department of Dermatology, University Hospital of Essen, Essen, Germany; 799 Bioinformatics and Omics Data Analytics, German Cancer Research Center (DKFZ), Heidelberg, Germany; 800 Department of Urology, Charité Universitätsmedizin Berlin, Berlin, Germany; 801 Martini-Clinic, Prostate Cancer Center, University Medical Center Hamburg-Eppendorf, Hamburg, Germany; 802 Department of General Internal Medicine, University of Kiel, Kiel, Germany; 803 German Cancer Consortium (DKTK), Partner site Berlin, Berlin, Germany; 804 Cancer Research Institute, Beth Israel Deaconess Medical Center, Boston, MA USA; 805 University of Pittsburgh, Pittsburgh, PA USA; 806 Department of Ophthalmology and Ocular Genomics Institute, Massachusetts Eye and Ear, Harvard Medical School, Boston, MA USA; 807 Center for Psychiatric Genetics, NorthShore University HealthSystem, Evanston, IL USA; 808 Van Andel Research Institute, Grand Rapids, MI USA; 809 Laboratory of Molecular Medicine, Human Genome Center, Institute of Medical Science, University of Tokyo, Tokyo, Japan; 810 Japan Agency for Medical Research and Development, Tokyo, Japan; 811 Korea University, Seoul, South Korea; 812 Murtha Cancer Center, Walter Reed National Military Medical Center, Bethesda, MD USA; 813 Human Genetics, University of Kiel, Kiel, Germany; 814 Department of Oncologic Pathology, Dana-Farber Cancer Institute, Harvard Medical School, Boston, MA USA; 815 Oregon Health and Science University, Portland, OR USA; 816 Center for RNA Interference and Noncoding RNA, The University of Texas MD Anderson Cancer Center, Houston, TX USA; 817 Department of Experimental Therapeutics, The University of Texas MD Anderson Cancer Center, Houston, TX USA; 818 Department of Gynecologic Oncology and Reproductive Medicine, The University of Texas MD Anderson Cancer Center, Houston, TX USA; 819 University Hospitals Coventry and Warwickshire NHS Trust, Coventry, UK; 820 Department of Radiation Oncology, Radboud University Nijmegen Medical Centre, Nijmegen, GA The Netherlands; 821 Institute for Genomics and Systems Biology, University of Chicago, Chicago, IL USA; 822 Clinic for Hematology and Oncology, St.-Antonius-Hospital, Eschweiler, Germany; 823 Computational and Systems Biology Program, Memorial Sloan Kettering Cancer Center, New York, NY USA; 824 University of Iceland, Reykjavik, Iceland; 825 Division of Computational Genomics and Systems Genetics, German Cancer Research Center (DKFZ), Heidelberg, Germany; 826 Dundee Cancer Centre, Ninewells Hospital, Dundee, UK; 827 Department for Internal Medicine III, University of Ulm and University Hospital of Ulm, Ulm, Germany; 828 Institut Curie, INSERM Unit 830, Paris, France; 829 Department of Gastroenterology and Hepatology, Yokohama City University Graduate School of Medicine, Kanagawa, Japan; 830 Department of Laboratory Medicine, Radboud University Nijmegen Medical Centre, Nijmegen, GA The Netherlands; 831 Division of Cancer Genome Research, German Cancer Research Center (DKFZ), Heidelberg, Germany; 832 Department of General Surgery, Singapore General Hospital, Singapore, Singapore; 833 Cancer Science Institute of Singapore, National University of Singapore, Singapore, Singapore; 834 Department of Medical and Clinical Genetics, Genome-Scale Biology Research Program, University of Helsinki, Helsinki, Finland; 835 East Anglian Medical Genetics Service, Cambridge University Hospitals NHS Foundation Trust, Cambridge, UK; 836 Irving Institute for Cancer Dynamics, Columbia University, New York, NY USA; 837 Institute of Molecular and Cell Biology, Singapore, Singapore; 838 Laboratory of Cancer Epigenome, Division of Medical Science, National Cancer Centre Singapore, Singapore, Singapore; 839 Universite Lyon, INCa-Synergie, Centre Léon Bérard, Lyon, France; 840 Department of Urology, Mayo Clinic, Rochester, MN USA; 841 Royal National Orthopaedic Hospital - Stanmore, Stanmore, Middlesex UK; 842 Department of Biochemistry, Genetics and Immunology, University of Vigo, Vigo, Spain; 843 Giovanni Paolo II / I.R.C.C.S. Cancer Institute, Bari, BA Italy; 844 Neuroblastoma Genomics, German Cancer Research Center (DKFZ), Heidelberg, Germany; 845 Fondazione Policlinico Universitario Gemelli IRCCS, Rome, Italy, Rome, Italy; 846 University of Verona, Verona, Italy; 847 Centre National de Génotypage, CEA - Institute de Génomique, Evry, France; 848 CAPHRI Research School, Maastricht University, Maastricht, ER The Netherlands; 849 Department of Biopathology, Centre Léon Bérard, Lyon, France; 850 Université Claude Bernard Lyon 1, Villeurbanne, France; 851 Core Research for Evolutional Science and Technology (CREST), JST, Tokyo, Japan; 852 Department of Biological Sciences, Laboratory for Medical Science Mathematics, Graduate School of Science, University of Tokyo, Yokohama, Japan; 853 Department of Medical Science Mathematics, Medical Research Institute, Tokyo Medical and Dental University (TMDU), Tokyo, Japan; 854 Cancer Ageing and Somatic Mutation Programme, Wellcome Sanger Institute, Hinxton, UK; 855 University Hospitals Birmingham NHS Foundation Trust, Birmingham, UK; 856 Centre for Cancer Research and Cell Biology, Queen’s University, Belfast, UK; 857 Breast Medical Oncology, The University of Texas MD Anderson Cancer Center, Houston, TX USA; 858 Department of Surgery, Johns Hopkins University School of Medicine, Baltimore, MD USA; 859 Department of Oncology-Pathology, Science for Life Laboratory, Karolinska Institute, Stockholm, Sweden; 860 School of Cancer Sciences, Faculty of Medicine, University of Southampton, Southampton, UK; 861 Department of Gene Technology, Tallinn University of Technology, Tallinn, Estonia; 862 Genetics and Genome Biology Program, SickKids Research Institute, The Hospital for Sick Children, Toronto, ON Canada; 863 Departments of Neurosurgery and Hematology and Medical Oncology, Winship Cancer Institute and School of Medicine, Emory University, Atlanta, GA USA; 864 Department of Clinical and Molecular Medicine, Faculty of Medicine and Health Sciences, Norwegian University of Science and Technology, Trondheim, Norway; 865 Argmix Consulting, North Vancouver, BC Canada; 866 Department of Information Technology, Ghent University, Interuniversitair Micro-Electronica Centrum (IMEC), Ghent, Belgium; 867 Nuffield Department of Surgical Sciences, John Radcliffe Hospital, University of Oxford, Oxford, UK; 868 Institute of Mathematics and Computer Science, University of Latvia, Riga, LV Latvia; 869 Discipline of Pathology, Sydney Medical School, University of Sydney, Sydney, NSW Australia; 870 Department of Applied Mathematics and Theoretical Physics, Centre for Mathematical Sciences, University of Cambridge, Cambridge, UK; 871 Department of Epidemiology and Biostatistics, Memorial Sloan Kettering Cancer Center, New York, NY USA; 872 Department of Statistics, Columbia University, New York, NY USA; 873 Department of Immunology, Genetics and Pathology, Science for Life Laboratory, Uppsala University, Uppsala, Sweden; 874 School of Electronic and Information Engineering, Xi’an Jiaotong University, Xi’an, China; 875 Department of Histopathology, Cambridge University Hospitals NHS Foundation Trust, Cambridge, UK; 876 Oxford NIHR Biomedical Research Centre, University of Oxford, Oxford, UK; 877 Georgia Regents University Cancer Center, Augusta, GA USA; 878 Wythenshawe Hospital, Manchester, UK; 879 Department of Genetics, Washington University School of Medicine, St.Louis, MO USA; 880 Department of Biological Oceanography, Leibniz Institute of Baltic Sea Research, Rostock, Germany; 881 Wellcome Centre for Human Genetics, University of Oxford, Oxford, UK; 882 Department of Molecular and Human Genetics, Baylor College of Medicine, Houston, TX USA; 883 Thoracic Oncology Laboratory, Mayo Clinic, Rochester, MN USA; 884 Institute for Genomic Medicine, Nationwide Children’s Hospital, Columbus, OH USA; 885 Department of Obstetrics and Gynecology, Division of Gynecologic Oncology, Mayo Clinic, Rochester, MN USA; 886 International Institute for Molecular Oncology, Poznań, Poland; 887 Poznan University of Medical Sciences, Poznań, Poland; 888 Genomics and Proteomics Core Facility High Throughput Sequencing Unit, German Cancer Research Center (DKFZ), Heidelberg, Germany; 889 NCCS-VARI Translational Research Laboratory, National Cancer Centre Singapore, Singapore, Singapore; 890 Edison Family Center for Genome Sciences and Systems Biology, Washington University, St. Louis, MO USA; 891 MRC-University of Glasgow Centre for Virus Research, Glasgow, UK; 892 Department of Medical Informatics and Clinical Epidemiology, Division of Bioinformatics and Computational Biology, OHSU Knight Cancer Institute, Oregon Health and Science University, Portland, OR USA; 893 School of Electronic Information and Communications, Huazhong University of Science and Technology, Wuhan, China; 894 Department of Applied Mathematics and Statistics, Johns Hopkins University, Baltimore, MD USA; 895 Department of Cancer Genome Informatics, Graduate School of Medicine, Osaka University, Osaka, Japan; 896 Institute of Computer Science, Heidelberg University, Heidelberg, Germany; 897 School of Mathematics and Statistics, University of Sydney, Sydney, NSW Australia; 898 Ben May Department for Cancer Research, University of Chicago, Chicago, IL USA; 899 Department of Human Genetics, University of Chicago, Chicago, IL USA; 900 Tri-Institutional PhD Program in Computational Biology and Medicine, Weill Cornell Medicine, New York, NY USA; 901 The First Affiliated Hospital, Xi’an Jiaotong University, Xi’an, China; 902 Department of Medicine and Therapeutics, The Chinese University of Hong Kong, Shatin, NT, Hong Kong China; 903 Department of Biostatistics, The University of Texas MD Anderson Cancer Center, Houston, TX USA; 904 Duke-NUS Medical School, Singapore, Singapore; 905 Department of Surgery, Ruijin Hospital, Shanghai Jiaotong University School of Medicine, Shanghai, China; 906 School of Computing Science, University of Glasgow, Glasgow, UK; 907 Division of Orthopaedic Surgery, Oslo University Hospital, Oslo, Norway; 908 Eastern Clinical School, Monash University, Melbourne, VIC Australia; 909 Epworth HealthCare, Richmond, VIC Australia; 910 Department of Biostatistics and Computational Biology, Dana-Farber Cancer Institute and Harvard Medical School, Boston, MA USA; 911 Department of Biomedical Informatics, College of Medicine, The Ohio State University, Columbus, OH USA; 912 The Ohio State University Comprehensive Cancer Center (OSUCCC – James), Columbus, OH USA; 913 The University of Texas School of Biomedical Informatics (SBMI) at Houston, Houston, TX USA; 914 Department of Biostatistics, University of North Carolina at Chapel Hill, Chapel Hill, NC USA; 915 Department of Biochemistry and Molecular Genetics, Feinberg School of Medicine, Northwestern University, Chicago, IL USA; 916 Faculty of Medicine and Health, University of Sydney, Sydney, NSW Australia; 917 Department of Pathology, Erasmus Medical Center Rotterdam, Rotterdam, GD The Netherlands; 918 Division of Molecular Carcinogenesis, The Netherlands Cancer Institute, Amsterdam, CX The Netherlands; 919 Institute of Molecular Life Sciences and Swiss Institute of Bioinformatics, University of Zurich, Zurich, Switzerland

**Keywords:** Cancer genetics, Mutation

Correction to: *Nature* 10.1038/s41586-020-1943-3 Published online 5 February 2020

In the published version of this paper, the members of the Pan-Cancer Analysis of Whole Genomes (PCAWG) Consortium were listed in the Supplementary Information; however, these members should have been included in the main paper. The original Article has been corrected to include the members and affiliations of the PCAWG Consortium in the main paper; the corrections have been made to the HTML version of the Article but not the PDF version. Additional minor corrections to affiliations have been made to the PDF and HTML versions of the original Article for consistency of information between the PCAWG list and the main paper.

