# Supplementary material for: The repertoire of mutational signatures in human cancer
Source: Nature. 2020 Feb 5;578(7793):94–101. doi: 10.1038/s41586-020-1943-3 (PMC7054213; doi:10.1038/s41586-020-1943-3)
Supplement: Supplementary file 1 — This file contains Supplementary Table 1: Summary of datasets used in this paper. [file 41586_2020_1943_MOESM1_ESM.pdf]

nature

https://doi.org/10.1038/s41586-020-1943-3

Supplementary information

The repertoire of mutational signatures in human cancer

In the format provided by the authors and unedited

The number of DBSs is proportional to the number of SBSs, with few exceptions

Supplementary Table 1. Summary of datasets used in this paper. Datasets are listed with a Synapse ID (accession number), which refers to data organized on the Synapse platform (https://www.synapse.org/). Every file has been mirrored at the International Cancer Genome Consortium Data Coordination Center (ICGC DCC) at the indicated URL and filename. All data listed in this table is open-access.

<table>
<tr><th>Label</th><th>Synapse ID</th><th>ICGC DCC URL</th><th>ICGC DCC File or folder name</th></tr>
<tr><td colspan="4">Description: This manuscript used mutational signature data from many sources in addition to the somatic mutation calls from the ICGC/TCGA PCAWG consortium; this table details these sources.</td></tr>
<tr><td>Catalogs of mutational spectra</td><td>syn11801889</td><td>https://dcc.icgc.org/releases/PCAWG/mutational_signatures/</td><td>Input_Data_PCAWG7_23K_Spectra_DB</td></tr>
<tr><td colspan="4">Description: This folder contains the main input data for the analyses reported here, primarily the catalogs of observed mutational spectra on which SigProfiler and SignatureAnalyzer were run.</td></tr>
<tr><td>Comparisons of mutational signatures extracted by different methods.</td><td>syn12016215</td><td>https://dcc.icgc.org/releases/PCAWG/mutational_signatures/Signatures/Comparisons</td><td>SBS_signatures_comparisons_2019_07_03.xlsx</td></tr>
<tr><td colspan="4">Description: This table compares signatures extracted by SigProfiler and SignatureAnalyzer and using 96-channel single-base-substitution (SBS) mutation classification and the "COMPOSITE" mutation classification (SBSs in pentanucleotide context plus doublet base substitutions plus indels), and also compares signature to COSMICv2 (https://cancer.sanger.ac.uk/cosmic/signatures_v2).</td></tr>
<tr><td>Reconstruction cosine similarity</td><td>syn12169204</td><td>https://dcc.icgc.org/releases/PCAWG/mutational_signatures/Signatures_in_Samples</td><td>Reconstruction_cosine_similarity.xlsx</td></tr>
<tr><td colspan="4">Description: This table (1) compares cosine similarity between the original and reconstructed spectra and (2) provides the numbers of signatures attributed by SigProfiler and SignatureAnalyzer to each tumour. For SignatureAnalyzer, signatures contributing < 100 mutations were omitted.</td></tr>
<tr><td>SignatureAnalyzer and SigProfiler attributions compared</td><td>syn12177011</td><td>https://dcc.icgc.org/releases/PCAWG/mutational_signatures/Signatures/Comparisons</td><td>All-SBS-sigs-attributions-comparison.pdf</td></tr>
<tr><td colspan="4">Description: These figures compare SignatureAnalyzer and SigProfiler single-base-substitution signature attributions (37 figures).</td></tr>
<tr><td>Mutational signatures extracted</td><td>syn11738307</td><td>https://dcc.icgc.org/releases/PCAWG/mutational_s</td><td>SA_Signatures</td></tr>
</table>

by SignatureAnalyzer		ignatures/Signatures	
**Description:** This folder contains all mutational signatures extracted by SignatureAnalyzer both in digital form and as plots.			
Tallies of occurrences of triplet-base substitutions and above	syn11801938	https://dcc.icgc.org/releases/PCAWG/mutational_signatures/Input_Data_PCAWG7_23K_Spectra_DB	PCAWG7_triplet_quadruplet_etc_base_substitutions_occurence.csv
**Description:** Tallies, for each PCAWG whole-genome tumour, of occurrences of triplet-base substitutions, quadruplet base substitutions, and above.			
Catalogs of mutational spectra for the PCAWG whole-genome tumours	syn11726620	https://dcc.icgc.org/releases/PCAWG/mutational_signatures/Input_Data_PCAWG7_23K_Spectra_DB/Mutation_Catalogs_--_Spectra_of_Individual_Tumours	WGS_PCAWG_2018_02_09.zip
Analysis of DBSs expected by chance adjacency of SBSss	syn12177057	https://dcc.icgc.org/releases/PCAWG/mutational_signatures/Other_Analyses	Expected_DBSs_due_to_adjacent_SBSs.xlsx
**Description:** Analysis of doublet-base substitutions expected by chance adjacency of single-base substitutions.			
Stranded counts for DBS1	syn12177063	https://dcc.icgc.org/releases/PCAWG/mutational_signatures/Other_Analyses/DBS_and_ID_Transcriptional_Strand_Bias	DBS1_stranded_counts.xlsx
**Description:** Input data for transcriptional strand bias analysis of doublet base substitution signature DBS1.			
Stranded counts for DBS2	syn12177064	https://dcc.icgc.org/releases/PCAWG/mutational_signatures/Other_Analyses/DBS_and_ID_Transcriptional_Strand_Bias	DBS2_stranded_counts.xlsx
**Description:** Input data for transcriptional strand bias analysis of doublet base substitution signature DBS2.			
Stranded counts for ID3	syn12177065	https://dcc.icgc.org/releases/PCAWG/mutational_signatures/Other_Analyses/DBS_and_ID_Transcriptional_Strand_Bias	ID3_stranded_counts.xlsx

**Description:** Input data for transcriptional strand bias analysis of indel signature ID3.			
Examples of tumours with ID3 strand bias	syn12177066	https://dcc.icgc.org/releases/PCAWG/mutational_signatures/Other_Analyses/DBS_and_ID_Transcriptional_Strand_Bias	ID3_strand_bias_examples.pdf
**Description:** Plots of examples of tumours with transcriptional strand bias of indel signature ID3.			
Indel signature attributions	syn11738668	https://dcc.icgc.org/releases/PCAWG/mutational_signatures/Signatures_in_Samples/SP_Signatures_in_Samples	PCAWG_SigProfiler_ID_signatures_in_samples.csv
**Description:** SigProfiler attributions of indel signatures to each PCAWG whole genome tumour.			
Correlation of signature activity with age: SigProfiler	syn12030687	https://dcc.icgc.org/releases/PCAWG/mutational_signatures/Signatures_in_Samples/SP_Signatures_in_Samples	SigProfiler_age_correlation.xlsx
**Description:** Analysis of correlation of signature activity with age based on SigProfiler signatures and attributions.			
Correlation of signature activity with age: SignatureAnalyzer	syn20317940	https://dcc.icgc.org/releases/PCAWG/mutational_signatures/Signatures_in_Samples/SA_Signatures_in_Samples	SignatureAnalyzer_age_correlation.xlsx
**Description:** Analysis of correlation of signature activity with age based on SignatureAnalyzer signatures and attributions.			
Input for age correlation analysis	syn12217988	https://dcc.icgc.org/releases/PCAWG/mutational_signatures/Signatures_in_Samples/SP_Signatures_in_Samples	PCAWG7_age_information.xlsx
**Description:** Input age for age correlation analysis.			
Tumour classification for SignatureAnalyzer	syn11738314	https://dcc.icgc.org/releases/PCAWG/mutational_signatures/Signatures/SA_Signatures	SA_sample_classification.012718.txt
**Description:** Classification of PCAWG whole-genome tumours for analysis by SignatureAnalzyer, which analyzed hyper-mutated tumours separately.			
Collection of all signatures extracted	syn11738306	https://dcc.icgc.org/releases/PCAWG/mutational_s	SP_Signatures

by SigProfiler		ignatures/Signatures/	
**Description:** All reference and alternative signatures extracted by SigProfiler in digital form and with plots.			
Local signatures from PCAWG WGS data	syn12025142	https://dcc.icgc.org/releases/PCAWG/mutational_signatures/Signatures/SP_Signatures/SP_other_signatures	Signatures_from_PCAWG_WGS_Date
**Description:** "Local signatures" refers to signatures extracted from individual cancer types; these were extracted by SigProfiler.			
Top level folder for all attributions	syn11804065	https://dcc.icgc.org/releases/PCAWG/mutational_signatures/	Signatures_in_Samples
**Description:** Contains all attributions (i.e. assignments of signatures to individual tumours) for both SignatureAnalyzer and SigProfiler.			
Local extraction from melanoma in digital form	syn11853305	https://dcc.icgc.org/releases/PCAWG/mutational_signatures/Signatures/SP_Signatures/SP_other_signatures/Signatures_from_PCAWG_WGS_Date/PCAWG_WGS_Local_Signatures/SBS	sigProfiler_SBS_PCAWG_WGS_Skin-Melanoma_local_signatures.csv
**Description:** Results of SigProfiler single-base-substitution signature extraction from the PCAWG whole-genome skin melanomas in digital form.			
Plot of local extraction from melanoma	syn11853532	https://dcc.icgc.org/releases/PCAWG/mutational_signatures/Signatures/SP_Signatures/SP_other_signatures/Signatures_from_PCAWG_WGS_Date/PCAWG_WGS_Local_Signatures/SBS	sigProfiler_SBS_PCAWG_WGS_Skin-Melanoma_local_signatures.pdf
**Description:** Plots of the results of SigProfiler single-base-substitution signature extraction from the PCAWG whole-genome skin melanomas.			
Non-PCAWG whole-genome signatures	syn20710496	https://dcc.icgc.org/releases/PCAWG/mutational_signatures/Signatures/SP_Signatures/SP_other_signatures/	Non_PCAWG_WGS_Signatures
**Description:** Contains global and local (per-cancer-type) signature extracted from non-PCAWG whole-genome data by SigProfiler.			
Transcriptional	syn12026195	https://dcc.icgc.org/relea	Sigprofiler_Transcriptiona

strand bias signatures		ses/PCAWG/mutational_signatures/Signatures/SP_Signatures/SigProfiler_reference_signatures	l_Strand_Bias_Signatures
**Description:** Contains transcriptional strand bias single-base-substitution signatures as extracted by SigProfiler.			
SigProfiler signature assignment rules	syn12177009	https://dcc.icgc.org/releases/PCAWG/mutational_signatures/Code/SigProfiler_Code	SigProfiler_signature_assignment_rules.xlsx
**Descriptions:** Rules used by SigProfiler during signature assignment; please refer to https://github.com/AlexandrovLab/SigProfilerExtractor.			
Results of tests on synthetic data	syn18497223	https://dcc.icgc.org/releases/PCAWG/mutational_signatures/	Tests_on_Synthetic_Spectra
**Description:** Synthetic input data and the result of tests of signature extraction (discovery) by SigProfiler and SignatureAnalyzer on synthetic data sets with known mutational signatures			
Attributions of signatures to mutational classes	syn11804068	https://dcc.icgc.org/releases/PCAWG/mutational_signatures/	Attributions_to_Each_Mutational_Class
**Description:** Estimates of the probability that each signature was responsible for each mutational type (e.g. CTG > CAG) in individual tumours.			
