# Supplementary material for: The repertoire of mutational signatures in human cancer
Source: Nature. 2020 Feb 5;578(7793):94–101. doi: 10.1038/s41586-020-1943-3 (PMC7054213; doi:10.1038/s41586-020-1943-3)
Supplement: Supplementary file 4 — Supplementary Note 2: Additional methods details. [file 41586_2020_1943_MOESM4_ESM.pdf]

Supplementary Note 2

Additional Methods Details for

Alexandrov, Kim, Haradhvala, Huang, et al.

"The Repertoire of Mutational Signatures in Human Cancer", 2020

Principles and strategy of mutational signature analysis adopted in this report

Conceptual principles.

- Multiple mutational processes generate the somatic mutations present in each individual human cancer.
- Each mutational process generates a particular pattern of somatic mutations known as a mutational signature.
- Each mutational process may incorporate a component of DNA damage/modification, DNA repair and DNA replication, each of which may be part of normal or abnormal cell biology. Differences in any of the three components may result in a different mutational signature, thus, by definition, constituting a distinct mutational process.
- Multiple mutational processes operating continuously or intermittently during the cell lineage from the fertilised egg to the cancer cell may contribute to the aggregate set of mutations found in the cancer cell. Thus, the catalogue of somatic mutations from a single cancer sample often includes mutations of many different mutational signatures.

Aims of the study.

- To decipher the mutational signatures present in essentially the full set of whole genome and exome sequenced human cancers from which data is currently available and subsequently to estimate the contributions of each signature to each cancer genome.

Approach used.

- Several mathematical approaches have been used to deconvolute/extract the mutational signatures present in a set of mutational catalogues[1-11]. They are all based on the premise that different mutational processes (and thus their signatures) contribute to different extents to different samples within the set.
- Two independently developed methods based on NMF (SigProfiler and SignatureAnalyzer) were applied separately to the sets of mutational catalogues. By using two methods we aimed to provide perspective on the impact different methodologies can have on numbers of signatures generated, signature profiles and attributions. The two methods are described in detail below and the code for both is available as decribed in the Code Availability Statement. Results from the two methods have been compared (syn12177011, syn12016215).
- Briefly, SigProfiler employs an elaboration of previously presented approaches for signature extraction and for attribution of mutation counts to mutational signatures in individual tumours[2,12-14].
- Briefly, SignatureAnalyzer employs a Bayesian variant of NMF[3,4,11]. This method enables inferences for the number of signatures through the automatic relevance determination technique and delivers highly interpretable and sparse representations for both signature profiles and attributions at a balance between data fitting and model complexity.

- The methods that SigProfiler and SignatureAnalyzer use for determining the number of extracted signatures are presented in the detailed descriptions of each of these methods, below.
- Both methods assume that the spectra of individual tumours can be represented as linear combinations of signatures. Thus, if the combination of two simultaneously operating mutational processes were to create a signature profile that is not a linear combination of the two, both SigProfiler and SignatureAnalyzer would extract this as a separate signature. We believe this is the case for SBS20, which appears to be due to the simultaneous operation of *POLD1* mutation and mismatch repair deficiency.

Role of NMF in extraction and attribution of mutational signatures.

- NMF is the approximate representation of a nonnegative matrix V, in this case the observed mutational spectra (or profiles) of a set of tumours, as the product of two usually smaller nonnegative matrices, W and H, which are the signatures and the attributions respectively.
- In our experience, however, calculating a single NMF is rarely sufficient to allow confident extraction and attribution of signatures that reflect the underlying biological mutational processes. There are two main reasons for this:
    - The profiles of extracted signatures can vary substantially depending on the tumour samples present in V. For example, this may be especially evident when some tumours in V have high numbers of mutations (e.g., samples due to UV exposure or DNA mismatch repair deficiency), while others have low numbers. In situations such as this, signatures due to highly mutagenic processes sometimes capture mutations from other processes and also "bleed" into other signatures.
    - With multiple potentially similar signatures operating, there are multiple possible and reasonably accurate reconstruction solutions for each tumour, often with many small and/or biologically implausible contributions.
- To address these challenges two key additional analytic features have been incorporated into our analyses:
    - Both SigProfiler and SignatureAnalyzer carried out multiple signature extractions on different subsets of tumours, and indeed, each signature extraction by SigProfiler entails at least 256 and, in most cases, 1024 NMFs with different random initial conditions. We describe below how we selected representative mutational signature profiles.
    - Both SigProfiler and SignatureAnalyzer developed a process of attributing signature activities to tumours that is separate from the process of extracting (discovering) the signatures.
- The use of multiple extractions to support confidence in results:
    - SignatureAnalyzer, carried out the main extraction procedure on (1) the majority of the PCAWG tumours excluding certain highly mutated tumours and (2) the melanomas, microsatellite-instable tumours, and a single temozolomide-exposed tumour (syn11738314).
    - SigProfiler extracted signatures as follows (see also syn11738306):

        - Separate extraction of SBS, DBS, and ID signatures from all PCAWG whole-genomes together (the main source of the reference mutational signature).
        - Separate extraction of SBS, DBS, and ID signatures from PCAWG whole-genomes with each tumour type examined by itself.
        - Extraction of SBS signatures from all non-PCAWG whole-genomes together.
        - Extraction of SBS signatures from non-PCAWG whole-genomes with each tumour type examined by itself.
        - Separate extraction of SBS, DBS and ID signatures from all TCGA exomes together.
        - Separate extraction of SBS signatures from TCGA exomes with each tumour type examined by itself.
        - Separate extraction of SBS signatures from all non-TCGA exomes together.
        - Separate extraction of SBS signatures from non-TCGA exomes with each tumour type examined by itself.
        - Extraction of COMPOSITE signatures from all PCAWG whole-genomes together.

    This allowed the extraction of signatures that were not present in the PCAWG tumours (e.g., SBS42, which has been attributed to haloalkane exposure and seen only in whole exome sequencing data). It also served as an important validation, as extraction of similar signatures from single tumour types and other sample sets supports the correctness of the signature extracted from the PCAWG samples (syn12016215).
    - Signature extraction from each tumour type (or from some other subset of cancers) separately has the advantages of:
        - Usually including fewer (and different) mutational signatures in each tumour type sample set than in the set of all cancers together and thus fewer (and different) opportunities for inter-signature interference.
        - Allowing multiple independent opportunities for extraction of a signature that is present in multiple tumour types, and thus of obtaining validation/confirmation of the signature's existence and profile.
        - Allowing extraction of a signature that may (for a number of reasons) fail to be extracted in analysis of all tumour types together.
        - Providing primary evidence for the existence of the signature in each tumour type.
        - Allowing separation of highly mutated cancer types/samples from cancer types/samples with low mutation burdens.
    - Signature extraction from multiple tumour types together has the advantages of:
        - Usually including more samples with a particular signature than in each individual cancer type and thus being better powered to separate a signature from other partially correlated signatures and/or from signatures with similar profiles.

        - Providing a single profile for a signature rather than the multiple slightly different profiles which emerge from extraction of each tumour type separately.
- The profiles of the mutational signatures extracted from cancer are highly variable. They range from some that have contributions from mutations of all subtypes in the mutation classification ("flat" or "featureless" signatures, e.g., SBS5 and SBS40) to others that are essentially defined by mutations at only one (or a small number) of the mutation subtypes (e.g., signatures SBS2, SBS13, SBS10a and SBS10b). There appears to be less concordance between the results of SigProfiler and SignatureAnalyzer for flat signatures than for signatures with distinct features indicating that generally, these may be more difficult to accurately extract and distinguish from each other. However, there is experimental support for the existence of SBS5 and SBS3[15,16].
- We represented each signature as a single reference. This selection of a single reference signature does not exclude the possibility that signature profiles may show nuances and further complexity and may vary in different contexts (e.g., in different tissues). The rationale for selecting a single reference signature was the view that this would be a level of granularity useful to most researchers. For those with specialised interests in particular mutational processes and their components, we also provided the signatures extracted from individual tumour types, comprising PCAWG and non-PCAWG genomes and exomes (syn12025142).
- Attribution of signatures to cancer samples:
    - The reference signatures from SigProfiler and SignatureAnalyzer were used to estimate the number of mutations due to each signature in each tumour (syn11804065).
    - SigProfiler and SignatureAnalyzer differ in their approaches for attributing signatures. However, both incorporate a set of rules based on prior knowledge and biological plausibility, and incorporate techniques to encourage sparsity in the number of signatures attributed to a given tumour.
    - Sparsity (limiting the numbers of signatures and limiting the numbers of signatures attributed to each cancer sample) is an important concept and feature of both SigProfiler and SignatureAnalyzer (both in signature extraction and attribution). Our prior beliefs are that *(i)* there is a limited set of significantly contributing mutational processes (and hence a limited set of mutational signatures) operating to generate somatic mutations across all cancers and *(ii)* that a limited set of mutational processes contribute to individual cancer genomes (as opposed to all mutational signatures contributing to all samples). Our aim in discovering mutational signatures is to reflect the underlying biological processes and to attribute them appropriately. It is not a mathematical exercise in which the main objective and priority is to minimize the difference between $W \times H$ and the original spectra in V. Indeed, if the latter was the main aim, for 96 mutation classes a set of 96 signatures each constituted entirely of mutations in just one class (and therefore ignoring sparsity), will always provide error free reconstruction but will provide absolutely no information about underlying mutational processes.

Presentation of the results of signature extraction and attribution from SigProfiler and SignatureAnalyzer.

- The results (signatures and attributions) of the two methods have been presented separately. We have done this in preference to combining them. We have handled the two outputs in this way because we believe that this provides a simpler conceptual and technical basis on which the research community can understand the results, can employ the methods in future and can compare results with those shown in this paper. We also do not have a basis for believing that a combined/averaged/overlapping single result set is a better representation of the natural truth than either of the two result sets individually and do not have a well-founded and simple technical approach for combining them. We have, however, provided comparisons of the outputs.
- For brevity and for continuity with previous publications, the results from SigProfiler, a further elaborated version of previously described approaches[2,12-14] that generated the 30 signatures previously shown in COSMICv2[17], are shown in the main manuscript, and the results from SignatureAnalyzer in supplementary data (syn11738307).
- Nomenclature of signatures is based on and extends the nomenclature previously used in COSMIC (COSMICv2, https://cancer.sanger.ac.uk/cosmic/signatures_v2)[17].
- Both methods analysed each mutation type (SBSs, DBSs and IDs) separately and also together as a composite signature. In future, however, SigProfiler will usually use the separately extracted single base substitution, indel and doublet base substitution signatures as its standard. This generally facilitates portability, and comparison of signature profiles with those from a variety of sample sets including targeted sequences, exomes etc.
- SBS signatures reported in Supplementary Data include possible artefacts (https://cancer.sanger.ac.uk/cosmic/signatures/SBS/ and see below).

Quality control: annotating signatures as likely real or a possible artefact

- Sequencing artefacts and differences in analysis pipelines can also generate mutational signatures. We have annotated which signatures are likely real or "possible artefact".
- There are multiple reasons for believing a signature reflects a biological mutational signature rather than an artefact.
    - The input data supporting the signature seem correct: key mutational features of the putative signature look real in a mapped-read browser such as Integrative Genomics Viewer (IGV, https://software.broadinstitute.org/software/igv/), or characteristic mutations are experimentally confirmed in the tumour and normal samples. Inspection in a mapped read browser is especially important in checking for possible problems in potentially new signatures arising in datasets other than the highly scrutinized and checked PCAWG and TCGA sets. Features associated with experimental, mapping, or other computational artefacts include strong preference for the first read, very low variant allele fractions, variants in regions of low germ-line sequencing coverage, variants found near indels in low-complexity regions, variants from a signature only found in one sequencing centre etc.

- The 96-mutation profile and additional features (e.g., strand asymmetry, association with replication timing), are known to result from a particular process in experimental systems. Examples: UV, polymerase epsilon proofreading deficiency, aristolochic acid and cisplatin exposure.
- The putative signature is broadly consistent with previous biochemical knowledge of mutational processes (e.g., preference for G adducts in aflatoxin).
- The putative signature dominates the spectra of some tumours (column J of syn12016215).
- The putative mutational signature is consistently deciphered from multiple independent datasets; this indicates that the signatures is either a common sequencing artefact or something real.
- The putative signature correlates with known or suspected mutational exposures, endogenous processes, or repair defects, especially if some of those exposures/processes/repair defects result in overwhelming mutational spectra. Examples: melanoma / fair skin / UV exposure, POLE mutations, MMR deficiency and APOBEC germ line variants.
- The putative signature correlates with other clinical characteristics, such as age at diagnosis (examples SBS1 and SBS5) or tobacco smoking (SBS4).
- The mutational signature exhibits a strong transcriptional strand bias; it is hard to imagine an artefact with transcriptional strand bias.
- The putative signature shows association with other genomic features, such as microindels in homopolymers, replication strand, replication timing, or nucleosome occupancy.

Cancer sample sets on which different analyses have been conducted.

- Because PCAWG genomes are of high quality with respect to the calling of all mutation types, all our analyses (all types of signature extraction and all types of signature attribution) have been conducted on the 2,780 PCAWG genomes.
- SigProfiler also extracted SBS signatures from the non-PCAWG whole genomes, TCGA exomes, and non-TCGA exomes and attributed SBS signatures to them.
- ID signatures have been extracted and attributed to PCAWG genomes and to a subset of TCGA exomes with large numbers of indels (the latter SigProfiler only). We have not done this for indels in non-PCAWG whole genome sequences and non-TCGA exomes *(i)* because of the unknown and variable accuracy and standardisation of indel mutation calls from different groups generating the data, *(ii)* because in some cases no indel calls were provided by the data generator and *(iii)* because for exomes in most cases there would be very few mutations.
- DBS signatures have been extracted and attributed to PCAWG genomes and TCGA exomes only. We have not done this for the other categories of samples because of the unknown and variable quality of the mutation calls, the possibility that filters introduced for quality control might deliberately exclude doublet mutations, and the small numbers of doublet mutations in exomes.
- Consistent with the above, composite mutational signatures have only been extracted and attributed for PCAWG genomes.

- See also syn11738306.

Splitting of mutational signatures.

- Certain previously existing single signatures have split into multiple constituent signatures in this analysis. This is likely due to the existence of multiple, partially correlated mutational processes with the same initiating factor (for example, UV exposure) but subsequent differences in underlying mechanisms which differ in intensity in different tissues or other contexts. A previous example of this for which we have allocated different signature numbers is the split of the usually co-occurring but independently varying consequences of APOBEC mutagenesis into signatures SBS2 and SBS13 (https://cancer.sanger.ac.uk/cosmic/signatures/SBS/).
- Depending on the extent of correlation of the two signatures, and the available dataset/statistical power such signatures may manifest as a single signature, overlapping partially separated signatures or as two separate signatures.
- We are aware that splitting of signatures can also be a mathematical artefact. However, we have used multiple extractions to confirm and validate signature splits and applied the principle of sparsity to limit artefactual splits (https://cancer.sanger.ac.uk/cosmic/signatures/SBS/).
- In extraction of UV-related signatures we observed the following:
    - Partial splitting for "local analysis" of PCAWG melanoma in isolation (syn11853305; SBS7c & SBS7d are mixed).
    - Partial splitting for "local analysis" of non-PCAWG WGS melanomas (syn11853532; SBS7c is almost completely separated but still mixed with SBS7d).
    - Complete separation of SBS7a,b,c,d in the global analysis PCAWG WGS melanomas (https://cancer.sanger.ac.uk/cosmic/signatures/SBS/)
    - Complete separation of SBS7a,b,c,d in the global analysis of non-PCAWG WGS melanomas (syn20710496).
    - Also, as an additional note, there are 157,012 T>A mutations at TTT (essentially SBS7c) and 128,411 T>C mutations at GTT (essentially SBS7d) across all of PCAWG. Thus, SBS7c and SBS7d are needed to explain these mutations.
    - Although SBS7a,b,c,d are correlated, the mutations due to each of these signatures are present in varying proportions across tumours, and this is the reason that SigProfiler and SignatureAnalyzer separate them.

Better separation compared to COSMICv2 signatures

As described in the manuscript, all mutational signatures previously reported on COSMIC were confirmed in the new set of analyses with median cosine similarity of 0.95. However, the separation between the COSMICv2 mutational signatures

(https://cancer.sanger.ac.uk/cosmic/signatures_v2) is much worse compared to the separation between the PCAWG mutational signatures. One can easily discern this by visual examination of signature profiles. For example, in COSMICv2, signatures 5 and 16 have a cosine similarity of 0.90, thus making them hard to distinguish from one another. In contrast, in the current PCAWG analysis, SBS5 and SBS16 have a cosine similarity of 0.65. This allows unambiguously assigning SBS5 and SBS16 to different samples. In the PCAWG analysis, the larger number of samples has allowed reducing the bleeding between signatures and has given more unique and easily distinguishable signatures. One can evaluate the overall separation of a set of mutational signatures by examining the distribution of cosine similarities between the signatures in the set. The COSMICv2 signatures have a median cosine similarity between the signatures in COSMICv2 of 0.238. In contrast, the PCAWG signatures have a much lower median cosine similarity between the signatures in PCAWG of 0.098. This 2-fold reduction in similarity is highly statistically significant (p-value: 9.1×10^{-25}) and indicates a better separation between the signatures in the current PCAWG analysis.

Correlations of mutational signature activity with age

Prior to evaluating the association between age and the activity of a mutational signatures, all outliers for both age and numbers of mutations attributed to a signature in a cancer type were removed from the data. Outlier was defined as any value outside three standard deviations from the mean value. A robust linear regression model that estimates the slope of the line and whether this slope is significantly different from zero (F-test; p-value<0.05) was performed using the MATLAB function robustfit (https://www.mathworks.com/help/stats/robustfit.html) with default parameters. The p-values yielded from the F-tests were corrected using the Benjamini-Hochberg procedure for false discovery rate. Results are at syn12030687 and syn20317940.

SigProfiler overview

SigProfiler incorporates two distinct steps for identification of mutational signatures based on the previously described methodology[2,12-14]. The first step, SigProfilerExtraction, encompasses a hierarchical *de novo* extraction of mutational signatures based on somatic mutations and their immediate sequence context, while the second step, SigProfilerAttribution, focuses on accurately estimating the number of somatic mutations associated with each extracted mutational signature in each sample.

SigProfilerExtraction

(Note: This phase is termed "SigProfiler" in the MATLAB code and "SigProfilerExtractor" in the Python code, and we refer to them collectively as SigProfiler below and elsewhere). The hierarchical *de novo* extraction approach is an extension of our previous framework for analysis of mutational signatures (Extended Data Figure 8a)[2,13]. Briefly, for a given set of mutational catalogues, the previously developed algorithm was hierarchically applied to an input matrix $M \in \mathbb{R}_{+}^{K\times G}$ of non-negative integers with dimension $K \times G$, where K is the number of mutation types and G is the number of samples. This previously described algorithm deciphers a minimal set of mutational signatures that optimally explains the proportion of each mutation type and estimates the contribution of each signature to each sample. The algorithm uses multiple NMFs to identify the matrix of mutational signatures, $P \in \mathbb{R}_{+}^{K\times N}$, and the matrix of the

activities of these signatures, $E \in \mathbb{R}_{+}^{N \times G}$, as previously described[2]. The unknown number of signatures, *N*, is determined by semi-automated assessment of the stability and accuracy of solutions for a range of values for *N*, (Extended Data Figure 8a). Briefly, for each value of *N*, SigProfiler carries out a decomposition with multiple bootstrapped NMFs. For a given *N*, the multiple solutions are clustered using a modified version of k-means clustering (see Ref. [2] for details). The clustering is used to evaluate the stability of decomposition, i.e., whether solutions from different initial conditions converge to similar signatures, and the mean reconstruction error, i.e., the ability of each solution to explain the original data. The python version of SigProfiler proposes a value of *N* with a low mean reconstruction error and high stability. Both the MATLAB and python versions present solutions for the full range of *N*s for human review for possible failure to split or over splitting of signatures in light of considerations presented in this Supplementary Note under the headings "*Role of NMF in extraction and attribution of mutational signatures*" and "*Quality control: annotating signatures as likely real or a possible artefact*" and in light of consistency of signatures extracted from different sets of tumours.

The identification of *M* and *P* is done by minimizing the generalized Kullback-Leibler divergence:

$$\min_{P \in \mathbb{R}_{+}^{(K,N)} E \in \mathbb{R}_{+}^{(N,G)}} \sum_{ij} (M_{ij} log \frac{M_{ij}}{\widehat{M}_{ij}} - M_{ij} + \widehat{M}_{ij}),$$

where $\widehat{M} \in \mathbb{R}_{+}^{K \times G}$ is the unnormalized approximation of M, *i.e.*, $\widehat{M} = P \times E$. The framework is applied hierarchically to increase its ability to find mutational signatures generating few mutations or present in few samples. In detail, after application to a matrix *M* containing the original samples, the accuracy of reconstructing the mutational spectrum of each sample with the extracted mutational signatures is evaluated. Samples that are well-reconstructed are removed, after which the framework is applied to the remaining sub-matrix of *M*.

Transcriptional strand bias associated with mutational signatures was assessed by applying SigProfilerExtraction to catalogues of in-transcript mutations that capture strand information (192 mutations classes, syn12026195). These 192-class signatures were collapsed to strand-invariant 96-class signatures and compared to the signatures extracted from the 96-class data, revealing very high cosine similarities (median 0.90, column F in syn12016215).

SigProfilerAttribution (single sample attribution)
(Note: This phase is termed SigProfilerSingleSample in both the MATLAB and Python code). After signatures are discovered by SigProfilerExtraction, another procedure, SigProfilerAttribution, estimates their contributions to individual samples. For each examined sample, $C \in \mathbb{R}_{+}^{K \times 1}$, the estimation algorithm involves finding the minimum of the Frobenius norm of a constrained function (see below for constraints) for a set of vectors $S_{i=1..q} \in \mathrm{Q}$, where Q is a (not necessarily proper) subset of the set of mutational signatures, P, ie, $\mathrm{Q} \subseteq \mathrm{P}$.

$$min\left\|\vec{C}-\sum_{r=1}^{q}\left(\overrightarrow{S_r}\times E_r\right)\right\|_F^2 \quad (1)$$

In equation (1), $\vec{C}$ and each $\overrightarrow{S_r}$ are vectors of *K* nonnegative components reflecting, respectively, the mutational spectrum of a sample and the *r-th* reference mutational signature. All mutational signatures, $\overrightarrow{S_r}$, were identified in the SigProfilerExtraction step. Each E_r is unknown scalar reflecting the number of mutations contributed by signature $\overrightarrow{S_r}$ in the mutational spectrum $\vec{C}$. The minimization of equation (1) is always performed under two additional constraints: (i) $E_r \geq 0$ and (ii) $\|\vec{C}\|_1 \geq E_r$; The constrained minimization of equation (1) is performed using a nonlinear convex optimization programming solver using the interior-point algorithm[18].

SigProfilerAttribution follows a multistep process, wherein equation (1) is minimized multiple times with additional constraints (Extended Data Figure 8b).

In the first phase, the subset Q contains all signatures that were found by *SigProfilerExtraction* in the same cancer type as the examined sample. Furthermore, signatures violating biologically meaningful constraints based on transcriptional strand bias and/or total number of somatic mutations are excluded from the set Q (syn12177009). Further, any $\overrightarrow{S_r} \times E_r$ for which the cosine similarity between $\hat{C}$ and $\vec{C}$ is ≤ 0.01 are sequentially removed, where $\hat{C} = \sum_{r=1}^{q}\left(\overrightarrow{S_r} \times E_r\right)$. Let T be the final set of signatures attributed to the sample at the end of the first phase.

In the second phase, equation (1) is minimized by sequentially allowing each signature, $S_r \in P\backslash Q$,to be added provided that it increases the cosine similarity between $\hat{C}$ and $\vec{C}$ by >0.05. During this second phase, several additional biological conditions are enforced: *(i)* signatures SBS1 and SBS5 are allowed in all samples, *(ii)* if one connected SBS signature is found in a sample than another one is also allowed in the sample (e.g., if SBS17a is found in a sample then SBS17b is allowed in the sample).

SignatureAnalyzer overview

SignatureAnalyzer employs a Bayesian variant of NMF that infers the number of signatures through the automatic relevance determination technique and delivers highly interpretable and sparse representations for both signature profiles and attributions that strike a balance between data fitting and model complexity. Please see references [3,4,11] for more details.

SignatureAnalyzer signature extraction

In 2,780 PCAWG samples, we applied a two-step signature extraction strategy using 1536 penta-nucleotide contexts for SBSs, 83 ID features, and 78 DBS features. In addition to separate extraction of SBS, ID, and DBS signatures, we performed a "COMPOSITE" signature extraction based on all 1697 features (1536 SBS + 78 DBS + 83 ID). For SBSs, the 1536 SBS COMPOSITE signatures are preferred, and for DBSs and IDs, the separately extracted signatures are preferred.

In step 1 of the two-step extraction process, global signature extraction was performed for the low mutation burden samples (n = 2,624). These excluded hyper-mutated tumours: those with putative polymerase epsilon (POLE) defects or mismatch repair defects (microsatellite instable tumours - MSI), skin tumours (which had intense UV mutagenesis), and one tumour with temozolomide (TMZ) exposure. Because SignatureAnalyzer's underlying algorithm performs a stochastic search, different runs can produce different results. In step 1 we ran SignatureAnalyzer 10 times and selected the solution with the highest posterior probability. In step 2, additional signatures unique to hyper-mutated samples were extracted (again selecting the highest posterior probability over 10 runs), while allowing all signatures found in the low mutation burden-samples to explain some of the spectra of hyper-mutated samples. This approach was designed to minimize a well-known "signature bleeding" effect or a bias of hyper- or ultra-mutated samples on the signature extraction. In addition, this approach provided information about which signatures are unique to the hyper-mutated samples which is later used when attributing signatures to samples.

SignatureAnalyzer signature attribution

A similar strategy was used for signature attribution; we performed a separate attribution process for low- and hyper-mutated samples in all COMPOSITE, SBS, DBS, and ID signatures. For downstream analyses, we preferred to use the COMPOSITE attributions for SBSs and the separately calculated attributions for DBSs and IDs. Signature attribution in low-mutation burden samples was performed separately in each tumour type (e.g., Biliary-AdenoCA, Bladder-TCC, Bone-Osteosarc, etc.). Attribution was also performed separately in the combined MSI (n=39), POLE (n=9), skin melanoma (n=107), and TMZ-exposed samples (syn11738314). In both groups, signature availability (i.e., which signatures were active or not) was primarily inferred through the automatic relevance determination process applied to the activity matrix H only, while fixing the signature matrix, *W*. The attribution in low-mutation burden samples was performed using only signatures found in the step 1 of the signature extraction. Two additional rules were applied in SBS signature attribution to enforce biological plausibility and minimize a signature bleeding: *(i)* allow signature SBS4 (smoking signature) only in lung and head and neck cases; *(ii)* allow signature SBS11 (TMZ signature) in a single GBM sample. This was enforced by introducing a binary, signature-by-sample, signature indicator matrix Z (1 - allowed and 0 - not allowed), which was multiplied by the *H* matrix in every multiplication update of *H*. No additional rules were applied to ID or DBS signature attributions, except that signatures found in hyper-mutated samples were not allowed in low-mutation burden samples.

Tests on Synthetic Data

Our goal was to evaluate SignatureAnalyzer (SA) and SigProfiler (SP) on realistic synthetic data. We operationally defined "realistic" as corresponding to either SA's or SP's analysis of the PCAWG genome data. SA's reference signature profiles were based on "COMPOSITE" signatures, consisting of 1536 strand-agnostic single base substitutions (SBSs) in pentanucleotide context, 78 doublet base substitutions and 83 types of small insertions and deletions, for a total of 1,697 mutation types. SP's reference analysis was based on strand-agnostic single base substitutions in the context of one 5' and one 3' base; we term this "SBS96" data. For each test, we generated

two sets of "realistic" data: *SP-realistic*, based on SP's reference signatures and attributions, and *SA-realistic*, based on SA's reference signatures and attributions, as well as two other types of data that involved using SA profiles with SP attributions and vice versa.

Generating synthetic data – overview. For tests (i) through (x) below, Synthetic data for sets of synthetic tumours of a given cancer type, *t*, were generated based on three parameters that were in turn based on the observed statistics for each signature, *s*, in cancer type *t*:

π, the proportion of tumours of cancer type *t* with signature *s*

μ, the mean of $\log_{10}$ of the number of *s* mutations across those tumours of type *t* that have signature *s*

σ, the standard deviation of $\log_{10}$ of the numbers of *s* mutations across those *t* tumours that have *s.*

To generate synthetic data,

(i) the proportion of tumours affected by *s* was drawn from the binomial distribution based on π,

(ii) the number of mutations due to *s* in an affected tumour was drawn from a normal distribution based on μ and σ.

The code used to generate the synthetic data and summarize SignatureAnalyzer and SigProfiler results is open-source and freely available as the SynSig package: https://github.com/steverozen/SynSig/tree/v0.2.0. The results are at syn18497223.

Description of each suite of synthetic data sets

***i.* Synthetic pancreatic adenocarcinoma (1,000 spectra).**

***ii.* 2,700 synthetic whole-genome mutational spectra – 300 spectra from each of 9 cancer types.** These spectra consist of 300 synthetic spectra from each of the following cancer types: bladder transitional cell carcinoma, oesophageal adenocarcinoma, breast adenocarcinoma, lung squamous cell carcinoma, renal cell carcinoma, ovarian adenocarcinoma, osteosarcoma, cervical adenocarcinoma, and stomach adenocarcinoma.

***iii.* Mutational spectra generated from combinations of flat, relatively featureless mutational signatures -- version 1,** 1000 synthetic tumours comprised of 500 synthetic Kidney-RCCs (high prevalence and mutation load from SBS5 and SBS40 signatures) and 500 synthetic ovarian adenocarcinomas (high prevalence of and mutation load from SBS3). This data set embodies tumours with high prevalence of the main flat signatures, SBS3, SBS5, and SBS40, in a realistic context.

***iv.* Mutational spectra generated from combinations of flat, relatively featureless mutational signatures -- version 2,** 1000 synthetic spectra all constructed entirely from SBS3, SBS5, and SBS40, using mutational loads modelled on kidney-RCC (SBS5 and SBS40) and ovarian adenocarcinoma (SBS3). Most synthetic spectra have contributions from all three signatures.

***v.* Mutational spectra generated from signatures with overlapping and potentially interfering profiles - version 1.** 500 synthetic bladder transitional cell carcinomas (high in SBS2 and SBS13) and 500 synthetic skin melanomas (high in SBS7a,b,c,d). The potential interference is between SBS2 (mainly C > T) and SBS7a,b (mainly C > T).

***vi.* Mutational spectra generated from signatures with overlapping and potentially interfering profiles - version 2.** 1000 synthetic tumours composed from SBS2 and SBS7a,b. Mutational load distributions were drawn from bladder transitional cell carcinoma (SBS2) and skin melanoma (SBS7a,b). Most spectra contain both signatures. The potential interference is between SBS2 (mainly C > T) and SBS7a,b (mainly C > T).

vii. Mutational spectra generated from combinations of signatures conferring high and low mutation burdens. Based on 500 synthetic non-hypermutated tumours (parameters for SBS1 and SBS5 estimated from colorectal and uterine adenocarcinomas) and 500 hyper-mutated tumours (parameters for SBS26 and SBS44 estimated from hypermutated colorectal and uterine adenocarcinomas). High and low mutation burden tumours are segregated for SignatureAnalyzer (which analyses low mutation burden tumours first, then high-burden tumours). SigProfiler analyses all tumours together.

viii. A set of 30 random 96-feature mutational signature profiles and a set of 30 random 1697-feature signature profiles (mimicking COMPOSITE signatures, which have 1697 mutation types). Each of these are used in two types of exposures, one with more (mean ~15.6) signatures per tumour and one with fewer (mean ~4) signatures per tumour.

ix. 2,700 whole-exome mutational spectra consisting of 300 synthetic spectra from each of 9 different cancer types. This test data set was generated from ***test ii*** by reducing the number of mutations of each type by 0.013 (approximately ratio of mutation counts between whole exome and whole genome mutational spectra).

Summary of findings: Both SA and SP extracted substantially fewer signatures in this test than in ***test ii***. In particular:

SA: SA extracted only 18 signatures from the SA-realistic whole-exome data in this suite, compared to the 40 signatures it extracted from the corresponding whole-genome synthetic data in ***test ii*** and compared to the 39 ground-truth signatures in the synthetic spectra. The average cosine similarity between ground-truth and extracted signatures for the synthetic exome data was 0.863, compared to 0.968 for the signatures extracted from the whole-genome spectra in ***test ii***.

SP: SP extracted only 8 signatures from the SP-realistic whole-exome data in this suite, compared to the 19 it extracted from the whole-genome data in ***test ii*** and the 21 ground-truth signatures in the synthetic spectra. The average cosine similarity between ground-truth and extracted signatures for the synthetic exome data was 0.825, compared to 0.965 for the signatures extracted from the whole-genome spectra in ***test ii***.

x. 1,350 synthetic whole-genome mutational spectra: 150 spectra from each of 9 cancer types. This test data set consisted of every other tumour from ***test ii***.

Summary of findings: On the SA-realistic synthetic data, SA extracted fewer signatures in this data set than in ***test ii***, and in fact the number of signatures extracted was closer to the ground truth and the cosine similarities were there higher. SA over-split in the corresponding set of 2,700 tumours, and we speculate that SA's tendency to over-split signatures is partly dependent on the number of input spectra, with the result that extraction on 1,350 led to less over-splitting. SP extracted fewer signatures on this data set than on ***test ii***. In particular:

SA: SA extracted 38 signatures from the SA-realistic data in this suite, compared to the 40 signatures it extracted from the 2,700 whole-genome spectra in ***test ii*** and compared to the 39 ground-truth signatures. The average cosine similarity between ground-truth and extracted signatures for 1,350 genomes was 0.979 compared to 0.968 for the signatures extracted from the 2,700 whole-genome spectra in ***test ii***.

SP: SP extracted 16 signatures from the SP-realistic data in this suite, compared to the 19 signatures it extracted from the 2,700 whole-genome spectra in ***test ii*** and the 21 ground-truth signatures. The average cosine similarity between ground-truth and extracted signatures for the 1,350 spectra was 0.939 compared to 0.965 for the signatures extracted from the 2,700 spectra in ***test ii***.

xi. Extraction of signatures from exome subsets of PCAWG mutational spectra. Our objective was to further test whether availability of mutations from whole-genome mutational spectra, as opposed to whole-exome spectra, enabled us to extract larger numbers of more accurate mutational signature profiles. In this test, we extracted signatures from mutational spectra that were based on only the exome regions of the actual PCAWG tumours (rather than on the purely synthetic data in ***test ix***). We next summarize our findings for each of the SBS, DBS, and ID mutational signatures.

xi-1 SBS signatures. SignatureAnalyzer on COMPOSITE mutational classes (1536 SBS in pentanucleotide context plus DBS and ID) extracted 12 mutational signature profiles from the whole-exome data, none of which strongly resembled any of the 58 signatures it extracted from the whole-genome data. However, some signatures were unions or splits of the signatures extracted from the whole genome data. For example, WI was a union of the APOBEC signatures BI_COMPOSITE_SBS2_P and BI_COMPOSITE_SBS13_P. More broadly, somewhat recognizable SBS portions of the signatures were combined with the DBS and ID portions of the signatures in

difficult-to-interpret combinations. We believe that SBS mutation counts were too low when spread across 1536 mutational classes to support robust mutational signature extraction.

SigProfiler on 96 SBS mutational classes extracted 17 mutational signature profiles from the exome data, compared to 48 that it extracted from the whole-genome data. The median cosine similarity of the exome-extracted signature profiles to the mutational signature profiles extracted from the whole genome data was 0.94. An outlier was SBS-E-2, which was a union of SBS2 and SBS13 (which tend to co-occur).

xi-2 DBS signatures. SignatureAnalyzer extracted 2 DBS signatures from the whole-exome data, compared to 15 DBS signatures that it extracted from the full whole genome data. One exome-extracted signature was essentially identical to BI_DBS1 (consisting almost entirely of CC > TT mutations), and one somewhat similar to BI_DBS2 (mostly CC > AA) but with many other mutational classes in addition.

SigProfiler extracted 3 DBS signatures from the whole-exome data, compared to the 11 DBS signatures that it extracted from the whole genome data. The exome-extracted signatures were good approximations of DBS1, DBS2, and DBS10 (cosine similarities 1, 0.93, and 0.98).

xi-3 ID signatures. SignatureAnalyzer extracted 4 ID signatures from the whole-exome data, compared to 29 ID signatures extracted from the whole-genome data. It extracted close approximations of BI_ID1_P and BI_ID2_P with cosine similarities 0.97 and 0.94. These are insertions (signature W.3) and deletions (signature W.1) of T:A in poly T:A. SignatureAnalyzer extracted 2 additional signatures. One of these (W.4) was a version of BI_ID4_P with several mutational classes absent. The other (W.2) appeared to be a union of many of the remaining ID signatures.

SigProfiler extracted 6 ID signatures from the whole-exome data, compared to the 17 ID signatures that it extracted from the whole genome data. Signatures ID-E-1, ID-E-2, ID-E-3, and ID-E-4 were good approximations of ID1, ID2, ID3, and ID4, respectively. An additional signature, ID-E-5, was approximately a union of ID6 and ID8. The remaining signature, ID-E-6 was a partial version (deletions in C homopolymers only) of ID7.

Detailed Summary of Results (including links to input synthetic data sets and the signature profiles extracted)*;* syn18497223 provides a table with the number of signatures extracted by SigProfiler and SignatureAnalyzer for each synthetic data set and the cosine similarities to the input ground-truth signatures. See above for overall interpretation of the results.

References

1 Alexandrov, L. B. *et al.* Signatures of mutational processes in human cancer. *Nature* **500**, 415-421 (2013).

2 Alexandrov, L. B., Nik-Zainal, S., Wedge, D. C., Campbell, P. J. & Stratton, M. R. Deciphering signatures of mutational processes operative in human cancer. *Cell Rep* **3**, 246-259 (2013).

3 Tan, V. Y. & Févotte, C. Automatic relevance determination in nonnegative matrix factorization with the/spl beta/-divergence. *IEEE Transactions on Pattern Analysis and Machine Intelligence* **35**, 1592-1605 (2013).

4 Kim, J. *et al.* Somatic ERCC2 mutations are associated with a distinct genomic signature in urothelial tumors. *Nat Genet* **48**, 600-606, doi:10.1038/ng.3557 (2016).

5 Fischer, A., Illingworth, C. J., Campbell, P. J. & Mustonen, V. EMu: probabilistic inference of mutational processes and their localization in the cancer genome. *Genome biology* **14**, R39, doi:10.1186/gb-2013-14-4-r39 (2013).

6 Roberts, N. D. *Patterns of somatic genome rearrangement in human cancer*, University of Cambridge, (2018).

7 Gehring, J. S., Fischer, B., Lawrence, M. & Huber, W. SomaticSignatures: inferring mutational signatures from single-nucleotide variants. *Bioinformatics* **31**, 3673-3675 (2015).

8 Rosales, R. A., Drummond, R. D., Valieris, R., Dias-Neto, E. & da Silva, I. T. signeR: an empirical Bayesian approach to mutational signature discovery. *Bioinformatics* **33**, 8-16 (2016).

9 Gori, K. & Baez-Ortega, A. sigfit: flexible Bayesian inference of mutational signatures. *bioRxiv*, 372896 (2018).

10 Blokzijl, F., Janssen, R., van Boxtel, R. & Cuppen, E. MutationalPatterns: comprehensive genome-wide analysis of mutational processes. *Genome medicine* **10**, 33 (2018).

11 Kasar, S. *et al.* Whole-genome sequencing reveals activation-induced cytidine deaminase signatures during indolent chronic lymphocytic leukaemia evolution. *Nat Commun* **6**, 8866, doi:10.1038/ncomms9866 (2015).

12 Alexandrov, L. B. *et al.* Clock-like mutational processes in human somatic cells. *Nat Genet* **47**, 1402-1407, doi:10.1038/ng.3441 (2015).

13 Nik-Zainal, S. *et al.* Landscape of somatic mutations in 560 breast cancer whole-genome sequences. *Nature* **534**, 47-54, doi:10.1038/nature17676 (2016).

14 WTSI Mutational Signature Framework, http://www.mathworks.com/matlabcentral/fileexchange/38724 (2013).

15 Blokzijl, F. *et al.* Tissue-specific mutation accumulation in human adult stem cells during life. *Nature* **538**, 260-264, doi:10.1038/nature19768 (2016).

16 Zámborszky, J. *et al.* Loss of BRCA1 or BRCA2 markedly increases the rate of base substitution mutagenesis and has distinct effects on genomic deletions. *Oncogene* **36**, 746 (2017).

17 Wellcome Trust Sanger Institute. *COSMIC, Catalog of Somatic Mutations in Cancer - Signatures of Mutational Processes in Human Cancer*, http://cancer.sanger.ac.uk/cosmic/signatures (2017).

18 Byrd, R. H., Hribar, M. E. & Nocedal, J. An interior point algorithm for large-scale nonlinear programming. *SIAM Journal on Optimization* **9**, 877-900 (1999).
